# Risk of Bias Assessment of Diagnostic Accuracy Studies Using QUADAS 2 by Large Language Models

**DOI:** 10.3390/diagnostics15121451

**Published:** 2025-06-06

**Authors:** Daniel-Corneliu Leucuța, Andrada Elena Urda-Cîmpean, Dan Istrate, Tudor Drugan

**Affiliations:** Department of Medical Informatics and Biostatistics, Iuliu Hațieganu University of Medicine and Pharmacy, 400349 Cluj-Napoca, Romania; dleucuta@umfcluj.ro (D.-C.L.); distrate@umfcluj.ro (D.I.); tdrugan@umfcluj.ro (T.D.)

**Keywords:** diagnostic accuracy, large language models, artificial intelligence, risk of bias, evidence-based medicine

## Abstract

**Background/Objectives:** Diagnostic accuracy studies are essential for the evaluation of the performance of medical tests. The risk of bias (RoB) for these studies is commonly assessed using the Quality Assessment of Diagnostic Accuracy Studies (QUADAS) tool. This study aimed to assess the capabilities and reasoning accuracy of large language models (LLMs) in evaluating the RoB in diagnostic accuracy studies, using QUADAS 2, compared to human experts. **Methods**: Four LLMs were used for the AI assessment: ChatGPT 4o model, X.AI Grok 3 model, Gemini 2.0 flash model, and DeepSeek V3 model. Ten recent open-access diagnostic accuracy studies were selected. Each article was independently assessed by human experts and by LLMs using QUADAS 2. **Results**: Out of 110 signaling questions assessments (11 questions for each of the 10 articles) by the four AI models, and the mean percentage of correct assessments of all the models was 72.95%. The most accurate model was Grok 3, followed by ChatGPT 4o, DeepSeek V3, and Gemini 2.0 Flash, with accuracies ranging from 74.45% to 67.27%. When analyzed by domain, the most accurate responses were for “flow and timing”, followed by “index test”, and then similarly for “patient selection” and “reference standard”. An extensive list of reasoning errors was documented. **Conclusions**: This study demonstrates that LLMs can achieve a moderate level of accuracy in evaluating the RoB in diagnostic accuracy studies. However, they are not yet a substitute for expert clinical and methodological judgment. LLMs may serve as complementary tools in systematic reviews, with compulsory human supervision.

## 1. Introduction

Diagnostic accuracy studies are essential in assessing the performance of medical tests. They inform clinical decision-making during diagnostic time and guide treatment strategies. To ensure the validity of these studies, methodological tools for assessing the risk of bias (RoB) have been developed in the framework of evidence-based medicine. One widely used tool is the Quality Assessment of Diagnostic Accuracy Studies (QUADAS), first published in 2003 [1] as an instrument to evaluate the methodological quality of diagnostic accuracy studies included in systematic reviews. In 2011, a second version, QUADAS-2, was introduced [2] to improve clarity, flexibility, and usability. On 30 April 2025, the third version of the tool was presented in the Methods for Evaluating Models, Tests and Biomarkers Conference [3], and a corresponding paper was submitted for publication.

Artificial intelligence (AI) is a term used to describe computational systems capable of performing tasks that typically require human intelligence, such as reasoning, learning, and language understanding [4,5]. Among the most sophisticated AI models are large language models (LLMs), which are deep learning architectures trained on enormous corpora of text to generate and comprehend language in a human-like manner [6,7]. These models use transformer-based architectures and a self-attention mechanism to detect linguistic patterns, semantic relationships, and contextual meaning in language [7,8]. There are many general-purpose LLMs, such as ChatGPT from OpenAI [9], Grok from X.AI [10], Gemini from Google [11], and DeepSeek [12]. LLMs are increasingly used in the biomedical field, including for single-document summarization of medical findings [13]. However, they were found to generate factually inconsistent summaries, overly persuasive or uncertain assertions, and contribute to misinformation [13]. These models often fail to prioritize the most informative information and perform less accurately when summarizing longer texts [13]. Nevertheless, LLMs have demonstrated the ability to outperform medical experts in clinical text summarization [14], and they show promising capabilities in understanding the context of evidence-based medicine [15]. Despite these advances, limitations remain, such as factual discrepancies, entity recognition problems, and domain inaccuracies.

To our knowledge, no previous study has evaluated the capacity of artificial intelligence models to assess the methodological quality of diagnostic accuracy studies. A critical milestone in the development of LLMs will be their ability to engage in self-learning. For this to be effective for medical diagnosis, it is essential that they acquire the ability to critically appraise scientific literature. Therefore, the aim of the study was to assess the capability of LLMs to evaluate the risk of bias in diagnostic accuracy studies, using QUADAS2, in comparison to human experts. The specific objectives were to evaluate the accuracy of the LLMs’ responses to QUADAS-2 signaling questions, identify the best-performing models, and characterize common reasoning errors made by LLMs.

## 2. Materials and Methods

### 2.1. Article Selection

Ten diagnostic accuracy articles were selected from Pubmed, using the following search strategy: (“diagnostic accuracy” [Title/Abstract]) AND (diabetes [Title/Abstract]), with the most recent papers first. A filter for free full-text articles was applied. The search strategy was performed on 9 May 2025. Only original articles on diagnostic accuracy assessment were included. Reviews, systematic reviews, editorials, and protocols were excluded. The articles were selected from various medical fields to ensure diversity. In addition, two articles unrelated to diagnostic accuracy were included to test whether the LLMs would recognize the inapplicability of QUADAS-2 in these cases and flag it.

### 2.2. Risk of Bias Evaluation

The selected articles were evaluated using QUADAS-2 tool [2]. This instrument evaluates risk of bias and applicability concerns across four domains: patient selection, index test, reference standard, and flow and timing. This study focused exclusively on the risk of bias evaluation. Each domain includes signaling questions to guide judgments with yes, no, or unclear answers. Based on these, the overall risk of bias for the domain is categorized as low, high, or unclear.

### 2.3. Human Risk of Bias Assessment

The human assessment was performed by two authors who independently assessed the quality of the articles and resolved their discrepancies by discussion and consensus.

### 2.4. Artificial Risk of Bias Intelligence Assessment

Four artificial intelligence generative large language models were used with their public web-based interfaces, for the AI assessment: ChatGPT 4o model, X.AI Grok 3 model, Gemini 2.0 flash model, and the DeepSeek V3 model. For all the models, the prompt was the same: “I will provide a scientific article, and I want you to use the QUADAS 2 assessment tool to assess the risk of bias for this article. Please wait for me to ask the signaling questions for each domain, and please provide the answer as yes/no/unclear/not applicable, while for the risk of bias, provide the answer as low, high, unclear, without any comments. After this answer, please provide the rationale for your answers”. Each article was uploaded as a PDF file, sourced from either the publisher’s site or PubMed Central. For each domain of QUADAS 2, the signaling questions and the risk of bias were then prompted to elicit the answers from the LLMs. Identical prompts were used for each domain across all models. A new session was initiated for each article to prevent carryover of contextual information.

### 2.5. Data Extraction

The responses for each signaling question, and overall risk of bias both from LLMs, and human experts, were recorded in a comparative table.

### 2.6. Verification of the Correctness of Artificial Intelligence Assessments

For each signaling question, two human experts established the correct answer by consensus. A new session was initiated for each article to prevent carryover of contextual information. In case there were reasoning errors, the errors were documented. All errors were grouped by domain and signaling questions and presented qualitatively.

### 2.7. Statistical Analysis

Categorical data were presented as counts and percentages. An assessment made by an LLM was considered correct only if it matched the human expert’s answer and included a proper argument. Identical answers to the human expert answer with an incorrect argument behind the answer were not considered correct.

## 3. Results

### 3.1. Study Characteristics

Ten diagnostic accuracy articles were selected from PubMed, and their full text was retrieved. There were two articles in cardiology: coronary artery disease [15] and carotid atherosclerosis [16]; two in the gastroenterology field: liver diseases [17,18]; one in neurology: diabetic neuropathy [19]; one in rheumatology: knee osteoarthritis [20]; one in sleep medicine: obstructive sleep apnea [21]; one in vascular surgery: peripheral artery disease [22]; and two in ophthalmology [23,24].

### 3.2. Risk of Bias Assessment of Individual Studies

The responses to the signaling questions and the assessment of the domain-specific risk of bias of human experts and of LLMs are presented in Table 1. In Supplementary Table S1, the complete responses of each LLM, as well as the reasoning errors, are presented.

### 3.3. Quantitative Accuracy Assessment of LLMs Evaluations Compared to Human Experts

**Figure 1:**
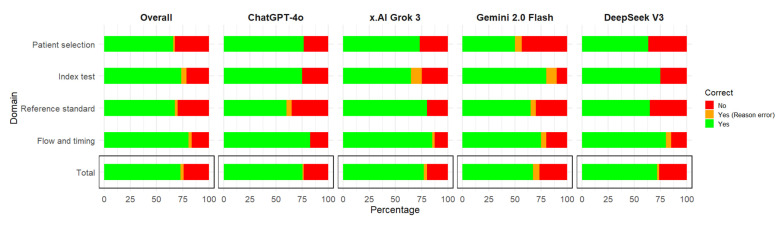
Correct responses for signaling questions by the domain of the QUADAS-2 risk of bias tool by large language models.

Out of 110 signaling questions assessments (11 questions for each of the 10 articles) by the four AI models, the mean percentage of correct assessments (where an assessment was considered correct if both the answer and the reasoning for the argument were correct) of all the models was 72.95%. The most accurate model was Grok 3, followed by ChatGPT 4o, DeepSeek V3, and Gemini 2.0 flash (Table 2, Figure 1), ranging from 74.45% to 67.27%. The overall answers that were correct but the reasoning was incorrect was 2.95%; the LLM with the highest percentage of these situations was Gemini 2.0 flash (6.36%) (Figure 1).

When assessed by domain, the most accurate responses were for flow and timing, followed by index test, and then similarly for patient selection and reference standard (Table 3, Figure 1), ranging from 80.63% to 63.75%. The best models for each domain were ChatGPT 4o and Grok 3 for patient selection; Gemini 2.0 flash for index test; Grok 3 for reference standard; ChatGPT 4o, Grok 3, and DeepSeek for flow and timing. The domain with the highest percentage of answers that were correct but the reasoning was incorrect was the index test (5%), followed by flow and timing (3.12%) (Figure 1).

**Figure 2:**
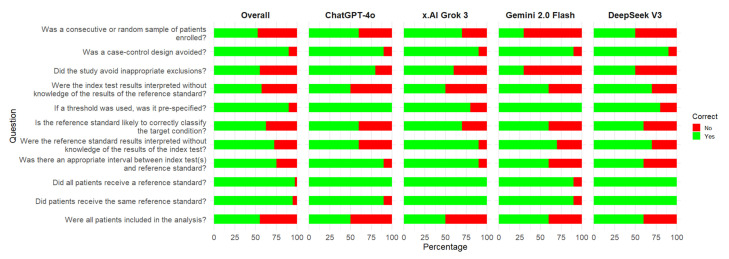
Correct responses for signaling questions of the QUADAS-2 risk of bias tool by large language models. An assessment was considered correct if both the answer and the reasoning for the argument were correct.

Concerning individual signaling questions, the most problematic ones for the LLMs were as follows (Figure 2): Was a consecutive or random sample of patients enrolled? Did the study avoid inappropriate exclusions? Were the index test results interpreted without knowledge of the results of the reference standard? Were all the patients included in the analysis? Is the reference standard likely to correctly classify the target condition?

### 3.4. Assessment of Reasoning Errors of LLMs

For each article and each signaling question, we qualitatively evaluated the reasoning errors made by the LLMs. We then synthesized these errors by QUADAS-2 domain.

#### 3.4.1. Patient Selection Reasoning Errors

Concerning the patient selection domain of QUADAS-2, the reasoning errors were observed by the following signaling question:

Was a consecutive or random sample of patients enrolled?

1. Misunderstanding of consecutive sampling in case-control or subgroup design.
  LLMs incorrectly concluded that consecutive sampling did not occur when authors declare a case-control design. However, consecutive sampling can be applied within case and control groups. Furthermore, there is a specific signaling question regarding the case-control design. Within the same framework, in one case, an LLM misinterpreted that having a target percentage of enrollment per group is incompatible with consecutive sampling. As stated before, within groups, consecutive sampling can be implemented. Consecutive sampling can and should be evaluated separately within each group (case-control or other design).
  Incorrectly assuming that using inclusion and exclusion criteria invalidates the possibility of having consecutive sampling. The selection criteria are essential in research methodology. One cannot include a participant who has exclusion criteria, just to make a sample “consecutive”.
2. Inference errors concerning consecutive sampling based on the author’s explicit reporting.
  Incorrectly reporting that consecutive sampling occurred, when the authors did not explicitly state it.
  Assuming the absence of explicitly stated sampling methods as evidence of their non-use.
  Missing a clearly reported use of consecutive sampling by the authors.
3. Ambiguity in authors’ descriptions of sampling methods. Choosing one of the authors’ statements as proof for the study design in case of ambiguity. In one case, one article stated that the sample was “convenience” and later that it was “consecutive”. These two are obviously contradicting each other. The LLM chose only one of them as an argument for the study design. The correct rationale would have been to consider that the design cannot be correctly interpreted based on this ambiguity.
4. Misinterpreting representativeness. In some cases, the LLM considered that the sample is not representative, since the authors stated they included patients with a suspicion of a certain diagnosis. For diagnostic tests, the population represented by those with a suspicion of a certain diagnosis is very common and has great utility for clinicians.

Was a case-control design avoided?

1. Incorrectly deciding that the design was case-control, focusing only on the authors’ declarations about the design of the study, instead of carefully assessing how the participants were selected. In one case, the authors declared the design as case-control. Later, it became clear that all the participants were representing one sample. After each participant was assessed regarding the presence of the disease, the participants were categorized as having or being without the disease. However, this does not represent a case-control design, where the source of the two samples should be separate.

Did the study avoid inappropriate exclusions?

1. Incorrect labeling of justified clinical exclusions is considered inappropriate. There were cases where the LLMs would consider that a specific exclusion is inappropriate, without presenting a medical argument. Upon clinical assessment, there were reasonable arguments in favor of the exclusion. Keeping those participants might have induced bias in the study. Thus, the exclusion was in fact appropriate.
2. Misinterpretation of missing information about exclusions.
  In some cases, the LLM could state there were no exclusions. But the absence of any statement of exclusions does not necessarily mean that the authors did not actually use exclusions, but failed to report them.
  Misunderstanding exclusions at the patient selection moment, and those after selection for the questions within the flow and timing domain. Exclusions can be made during patient selection. After the patients are selected, further exclusions can be made, which can be identified in the flow and timing domain with the following question: Were all the patients included in the analysis? In some cases, the LLM considered an exclusion made after the use of the index test (clearly after patient selection), as a patient selection bias instead of a flow and timing bias.
  Failing to recognize that the exclusion of unclear situations can induce bias. In some cases, the LLMs did not consider that the exclusion of patients due to poor-quality images would induce bias. In real-world settings, poor-quality images can occur. Their exclusion would artificially inflate the accuracy of diagnostic tests that do not have to deal with technical difficulties.

#### 3.4.2. Index Test Domain Reasoning Errors

Concerning the index test domain of QUADAS-2, the following reasoning errors were observed, by signaling question:

Were the index test results interpreted without knowledge of the results of the reference standard?

1. Misinterpreting the objectivity of the index test. In some cases, the LLM did not observe that certain tests are clearly objective (e.g., biomarkers, serum evaluations), and they cannot be influenced by the reference standard results.
2. Misinterpreting of test sequencing regarding the standard test. In some cases, the LLM did not observe that the standard test was carried out after the index test, thus making it impossible for the index test results to be influenced by the standard test.
3. Inversing the direction of blinding. In some cases, the LLM acknowledged that the observer who performed the standard test was blinded to the index test results. The LLM then incorrectly concluded that this implies that the one who performed the index test was blinded to the standard too.

If a threshold was used, was it pre-specified?

1. Misunderstanding of data-driven thresholds as prespecified. In some cases, the LLM considered the use of tertiles, or several operational thresholds (high specificity, high sensitivity, maximum gain points), as prespecified thresholds. In reality, these thresholds are calculated after the data are collected, not when writing the study protocol.

#### 3.4.3. Reference Standard Domain Reasoning Errors

Concerning the reference standard domain of QUADAS-2, the following reasoning errors were observed, by the following signaling question:

Is the reference standard likely to correctly classify the target condition?

1. Confusing frequent use of diagnostic tests as arguments for their validity. In some cases, the LLM would use as arguments for high diagnostic accuracy the fact that the test is widely used, or frequently applied, or a preferred method in clinical settings.
2. Confusing guideline-supported methods as arguments for a gold standard. In some cases, the LLM indicated that a test can be considered a gold standard because it is supported by a guideline. Some tests are less invasive, and this is why they are supported by guidelines, but they are not necessarily the gold standard for specific diagnostics.
3. Not knowing what a gold standard is in specific cases. In some cases, the LLM was not aware that a clinical test without imaging methods cannot accurately make a diagnosis.
4. Confusing high reliability with validity. In some cases, the LLM argued for the use of a test because of the high intra-rater agreement. High agreement, although desirable, does not imply high accuracy.
5. Influenced by the use of the word “standardized”. In some cases, the LLM considered the fact that the authors used the word standardized is an argument for a good standard test. The context was “standardized clinical history”. This word is not enough to guarantee high accuracy, and a standard test.
6. Failed to recognize that moderate agreement is problematic. In some cases, the LLM did not observe that the agreement between raters was moderate. While high agreement does not guarantee high accuracy, low agreement impacts accuracy.

Were the reference standard results interpreted without knowledge of the results of the index test?

1. Inversing the direction of blinding. In some cases, the LLM acknowledged that the observer who performed the index test was blinded to the reference test results. The LLM then incorrectly concluded that this implies that the one who performed the reference test was blinded to the index test.
2. Misunderstanding of the logic of time and the sequence of the tests regarding the standard test. In some cases, the LLM did not observe that the standard test was carried out before the index test (in some retrospective studies), thus making it impossible for the standard test results to be influenced by the index test.
3. Misunderstanding of blinding contexts. In some cases, the LLM identified that the observer lacked blinding for the study aim, and it interpreted that blinding was not employed for the reference standard. The absence of blinding for the aim is a different type of bias, one that is not concerning the test.
4. Correct answers with incorrect arguments. In some cases, the LLM gave correct answers, but used incorrect logic for the arguments.

#### 3.4.4. Flow and Timing Domain Reasoning Errors

Concerning the flow and timing domain of QUADAS-2, the reasoning errors were observed, by the following signaling question:

Was there an appropriate interval between index test (s) and reference standard?

1. Misinterpretation of time intervals due to the presence of specific wording. In some cases, the LLM observed that the authors stated that the design was cross-sectional, and used this as an argument for an appropriate interval between the tests. But, even in cross-sectional designs, the time between the two tests can be large enough to allow the disease to progress, and thus the tests to measure different things. The correct assessment should be based on specific information that clearly indicates the time, ignoring the reported design. In some cases of cohort studies, the LLM considered that the expression “collected during the same NHANES examination cycle or cohort (2017–2020)” implies that the timing between the index and standard test is short enough.
2. Failing to acknowledge the absence of reporting of timing between tests. In some cases, the LLM did not observe the absence of explicit reporting of timing between tests. Then, it assigned a low risk of bias instead of assigning an unclear risk of bias.
3. Incorrect assumption of time between tests based on ambiguous wording. In some cases, the authors stated that a test was performed in a “single session” or a “single sitting”. The other test was performed at another time. The LLM incorrectly inferred, based on that expression, that both tests were performed in the same sitting.
4. Misjudgment about the clinical relevance of timing intervals. In some cases, the LLM did not understand that, within a hospitalization time for a disease (which is usually not very long), although the time between the two assessments was not specified, due to the fact that the disease progression is very slow, the time elapsed between the tests is not problematic.
5. Failing to understand the time between assessments of the same imagistic method. In some cases, the study assessed the same medical images by humans and by algorithms. The LLM argued that the authors failed to mention the delay between the tests. But this does not make any sense in this context.

Did all patients receive a reference standard?

1. Misclassification of exclusions at enrollment vs. after enrollment. In some cases, the LLM incorrectly considered participant exclusions at patient selection time, as missing at analysis time.
2. Misinterpreting the selection of participants. In some cases, the LLM observed that from an initial sample of participants, only some of the participants were assessed by the authors in the study. This subgroup was assessed using both the index and standard tests. Thus, in reality, the apparent subgroup was in fact the actual patient selection group. The LLM, instead, considered that there was a problem of not applying the reference test to all participants.

Did all patients receive the same reference standard?

1. Failing to identify multiple reference standard tests that were used. In some cases, the LLM missed recognizing that there were two groups of assessors from different countries with different coefficients of agreement between their assessments of images.

Were all patients included in the analysis?

1. Failing to verify the completeness of data tables. In some cases, the LLMs did not check in the tables what the numbers were, so that exclusion could be deducted, even though they weren’t mentioned in the text.
2. Failing to recognize post-index-test exclusions. In some cases, the LLMs, did not identify that there was a problem of exclusion, in case where exclusions were made at the time of using the index test (to exclude poor quality images, or difficult cases). This is clearly not an exclusion relevant to patient selection time.
3. Failing to correctly differentiate exclusions at patient selection moment from those after enrolment, concerning the flow and timing domain.

Other qualitative observations on LLMs.

Overall, the subjective perception of the methodological proficiency of LLMs based on the arguments used for reasoning placed Grok 3 in the first position, being the more argumentative and verbose, followed by ChatGPT 4o. The shortest answers were given by DeepSeek V3.

All the models understood well the medical field terminology, abbreviations, and methodological terms.

The time of processing the PDF file was adequate and similar between ChatGPT 4o, Grok 3, and Gemini 2.0 flash, but longer for DeepSeek V3.

### 3.5. Assessment of Articles That Are Not of the Correct Study Type

Two articles that were of a different study type from diagnostic accuracy studies were assessed with the prespecified LLMs [25,26]. Grok AI gave the best answers, insisting on the fact that the type of article is not appropriate to be assessed with QUADAS-2, and suggested other quality assessment tools. The other LLMs tried to answer each question but mentioned that the type of article is of a different kind. Thus, many questions received answers that were not applicable. DeepSeek, having the shortest arguments for the answers, was the least transparent compared to the other LLMs in that the article type is not appropriate for QUADAS-2.

## 4. Discussion

The results of this study show that large language models (LLMs) can achieve a moderate level of accuracy in the assessment of the methodological quality of studies on diagnostic accuracy using the QUADAS-2 instrument. Across the four models tested, the average accuracy in correctly answering signaling questions was 72.95%. The best-performing model was Grok 3 (77.27%), followed by ChatGPT 4o (75.45%), DeepSeek V3 (71.82%), and Gemini 2.0 flash (67.27%). These results suggest that although current LLMs are not yet equivalent to expert human reviewers, they demonstrate considerable potential as adjunct tools in evidence quality assessment. Performance varied across domains: the highest accuracy was observed in the “flow and timing” domain (80.63%), followed by “index test” (73.75%), “patient selection” (65.83%), and “reference standard” (63.75%). These differences highlight areas in which LLM performance could be improved. In a small percentage, the LLMs offered the correct response, but the reasoning behind the response was erroneous. Overall, the findings support the potential utility of incorporating LLMs into system review processes, especially for structured tasks like signaling question assessment, but with compulsory human oversight.

Regarding LLM reasoning, errors were observed in all QUADAS-2 domains. In the patient selection domain, common issues included misinterpretation of consecutive sampling, particularly in the context of case-control designs, incorrect assumptions about sampling methods, and a failure to distinguish between exclusions made at the selection and analysis stages. LLMs also misclassified justified clinical exclusions as inappropriate.

In the index test domain, LLMs often misunderstood the objectivity of some laboratory tests, misjudged the timing sequence between index and reference tests, inverted the logic of blinding, and erroneously treated data-driven thresholds as prespecified.

Within the reference standard domain, errors included interpreting frequent use or guideline endorsement as evidence of diagnostic accuracy, confusing high reliability with validity, failing to recognize problematic moderate inter-rater agreement, and misapplying concepts of blinding and timing in retrospective settings.

Lastly, in the flow and timing domain, LLMs incorrectly assumed adequate intervals based solely on study design labels, failed to acknowledge missing timing information, misclassified exclusions at enrollment as analysis-time losses, did not recognize the use of multiple reference standards, and inadequately distinguished post-index-test exclusions from initial patient selection criteria.

Our qualitative reasoning error analysis identified several limitations of current LLMs. Thus, they can fail to integrate multiple pieces of contextual information across a study, leading to superficial or incomplete judgments. Existing LLMs also lack explicit mechanisms for handling uncertainty. When the original text of the article gives conflicting information, the models give spurious results. LLMs do not seem to understand some aspects of how things happen in real life, at least in medical clinical research scenarios. LLMs cannot reliably say when a question cannot be answered based on the text in the article, sometimes offering speculative or “hallucinated” justifications. Future improvements should focus on integrating a document-wide context, enhancing real-life logic in reasoning, and developing models that can more effectively communicate the existence of uncertainty.

Our results revealed differences in accuracy among the evaluated LLMs, spanning a 10% interval. Several factors may have contributed. Each LLM is trained on various corpora of text, architecture, and reinforcement learning strategies. These can influence the answers they provide. It is possible that some LLMs have greater exposure to medical literature than others and this can be reflected in their understanding of methodological specificities of the medical literature. Also, the corpora of text for some models can have more sources of text specialized in logical thinking, critical assessment, nuances, that might have helped some models behave better in risk of bias assessment. Reinforcement learning is a method of fine-tuning a model (usually with human feedback), so that it will respond in a certain manner. It is possible that some models had reinforcement learning that increased their critical assessment. All major LLMs, including the four that were assessed, have a built-in system prompt, by which their creators dictate how the model should behave when asked by clients. This system prompt might influence the length of the answers, explaining why Grok 3’s answers tended to be longer and more argumentative, and Google’s Gemini Flash 2.0 and DeepSeek V3 giving more concise answers. All system prompts can be overridden by client prompts. In fact, our prompt had an explicit demand that elicited a chain of thought reasoning the phrase “provide a rationale for your answers”. Chain of thought is a method that LLMs use to give an explanation or justification of its reasoning process, detailing the logical or inferential steps taken to reach that answer. All tested LLMs when asked as we did would answer using chain of thought. It is possible that the same model might have been influenced in their chain of thought, by the system prompt, by the reinforcement learning or even by their architecture.

Initially, we anticipated that state-of-the-art LLMs would perform similarly and accurately on the QUADAS-2 assessment, given the tool’s relative explicit structure especially in the signaling questions. However, it is documented that even among human reviewers, inter-rater reliability for QUADAS-2 is moderate to low, particularly in risk-of-bias domains [27]. Our study found that LLM accuracy was moderate overall but varied across models and domains. LLMs had better accuracy in flow and timing, while showing lower accuracy in patient selection or reference standards. These results suggest that both humans and LLMs face difficulties in RoB judgements. Our surprise was not that great in the accuracy of the models, but in the straightforward reasoning errors that we found. Some of the LLM errors tend to be systematic, and future models or prompt engineering must find ways to diminish them.

To assist readers interested in applying large language models to RoB assessment we list here several suggestions. Users should assess domain-specific accuracy by comparing LLM judgments to expert ratings across the key QUADAS-2 domains, as we see that performances vary by domain. Also signaling question-specific accuracy should be further assessed. Users should analyze the types of reasoning errors made by LLM (misinterpretations, mishandling in case of uncertainty of article text, logical flaws, or omissions), and determine whether these are systematic or sporadic. Users should quantify inter-rater agreement between LLM and human experts. Furthermore, we recommend prompt engineering experiments, where users test different instructions to optimize both the accuracy, clarity and depth of the LLM’s rationales. The list of errors found in our article could guide in creating prompts that would prevent LLMs from erring again. By adopting these suggestions, researchers and practitioners can more confidently use LLMs as supportive tools in systematic review workflows.

LLMs were found to be used as tools helping in literature reviews by Scherbakov et al. [28] in a systematic review in 2025. The number of papers that reported LLM usage was low, at 26. This is probably only the tip of the iceberg. One can suspect that authors are using the tools without reporting them for fear of not being considered as an untrustworthy article due to AI use. The stages of review that were reported from the highest use of LLMs to the lowest were as follows: searching, data extraction, title and abstract screening, evidence summarization, drafting manuscripts, and full text-screening [28]. The mean precision in title and abstract screening was low (63%), but better in extracting data by GPT models (83%) [28]. The study quality assessment was reported in less than a third of the articles. Searching for literature in the context of AIs consists in assisting in building search strategies for bibliographic databases [29]. Issues were found in this respect when LLMs hallucinated controlled vocabulary terms [30]. Besides advantages given by the use of LLMs in all these stages of review, problems were found in their use [29] that imply the need for human supervision.

To our knowledge, no prior studies have assessed LLM performance in evaluating risk of bias in diagnostic accuracy studies using QUADAS-2.

However, we found other studies assessing RoB in randomized controlled trials by LLMs. One study by Lai et al. [31] used a modified version of the Cochrane RoB tool developed by the CLARITY group at McMaster University [32] to assess randomized clinical trials. They reported 84.5% accuracy for ChatGPT, and of 89.5% for Claude (another LLM from Anthropic [33]), approximately ten percentage points higher than the results from our study. Of course, the RoB assessments and the selection of articles are not similar between the two types of articles, and this can explain the differences. A more recent study by the same first author, Lai et al. [34], assessed LLMs (Claude and Moonshot-v1-128k [34]) for data extraction and RoB assessment in alternative medicine trials reporting even higher accuracy (96.9% and 95.7%, respectively). Here, the differences compared to our results are higher, but the LLM models are different. One explanation for why other studies found higher accuracy might be that some of the articles included in their study might have been assessed for bias in systematic reviews that were in the training data of the LLMs. To avoid this possible error, in our study we selected very recent articles only. Another explanation might be that their assessments did not consider the reasoning of the model behind its choices. In our study we considered as correct only the answers that, besides being concordant with human experts, had a correct reasoning behind them.

We found a study by Hasan et al. [35] assessing RoB in non-randomized studies of interventions (ROBINS-I). They compared ChatGPT 4 with human assessment, regarding the ROBINS-I instrument [36], and the LLM achieved an agreement of 61%, thus clearly limiting the use of the LLM alone. Again, the differences can also be explained by the use of different RoB instruments and the selection of articles.

An interesting observation is that for some of the answers that were correct the reasoning behind the answer was incorrect. The percentage of these situations was low (2.95), the most frequently affected domain being the index test (5%). Chen et al. found, surprisingly, that reasoning models do not always say what they think [37]. Nevertheless, they insist that monitoring reasoning of the models remains an important way to identify undesirable behaviors, even if they cannot eliminate them [37].

### 4.1. Limitations

This study had several limitations. First, the sample of diagnostic accuracy articles that were selected was relatively small, which may limit the generalizability of our findings. To mitigate this, we selected articles covering a wide range of medical domains. Since only free recent papers were searched from one database, a selection bias cannot be excluded and the diversity of research topics may have been limited. Second, the qualitative assessment of reasoning errors made by LLMs involves subjective human evaluation. Third, we evaluated only the publicly available versions of the LLMs at the time of the study. Newer, more advanced versions, available via paid subscription may outperform the models tested here in terms of risk of bias assessment. Fourth, we used only one single standardized prompt to ensure comparability across LLMs. Different prompts for the same model would have elicited different responses with different accuracy in assessing the risk or bias. We used a simple prompt to enact a scenario where researchers that are not trained in prompt engineering would use LLMs assess the RoB for their systematic reviews. We acknowledge that more complex prompts could produce better answers or reasoning. Future studies should test different prompt methods and different versions of LLMs. These studies can make use in their prompting strategies of hints based on our article results, to avoid the reasoning errors that we observed.

### 4.2. Strengths

To our knowledge, this is the first study to evaluate the capability of large language models to assess the risk of bias in diagnostic accuracy studies using the QUADAS 2 tool compared with human expert judgment. The inclusion of studies from diverse medical specialties enhances the robustness and the practical relevance of our findings. Furthermore, our study qualitatively presents the reasoning errors made by the LLMs.

### 4.3. Future Perspectives

The findings of this study have significant utility for both clinical researchers and developers of AI models. For clinical researchers, understanding the specific reasoning errors commonly made by LLMs during risk-of-bias assessments with the QUADAS-2 tool draws an alarm signal that, for now, the technology is not mature enough to be used without supervision. For the moment, LLMs could be used as providing alternative perspectives, or helping to find potential issues in studies during the risk of bias assessment within systematic reviews. Their role, at present, can be seen as supporting human experts, rather than replacing expert assessment, especially in context of systematic reviews, and in clinical guideline development, where accuracy is paramount. Our findings, especially the reasoning errors can help in creating complex prompts or fine-tune them to improve the accuracy of current LLMs. For AI developers, the detailed reasoning errors that we catalogued can guide future refinement in model training and prompt engineering. Ultimately, our work may help guide the iterative development of hybrid, AI-assisted review tools that combine the efficiency of automation with the reliability of expert oversight.

## 5. Conclusions

This study shows that large language models are capable of obtaining a moderate level of accuracy in evaluating the risk of bias in diagnostic accuracy studies by means of the QUADAS-2 tool, with a performance of between 67% and 77% across models. For the moment, they are not yet a substitute for expert clinical and methodological judgment. LLMs might be used as complementary assistants for systematic reviews when using structured and rule-based evaluations, but with compulsory human supervision. Additional refinement and validation are required in order to integrate safely and efficiently into evidence-based medicine processes.

## Figures and Tables

**Table 1:** Risk of bias assessment using QUADAS-2 tool, as performed by human experts and four artificial intelligence generative large language models.

**QUADAS 2 Domains, Signaling Questions/Study**	**Van Kleef, 2025 [17]**					**Subramani, 2025 [19]**				
**Assessor**	**Humans**	**ChatGPT4o**	**x.AI Grok 3**	**Gemini 2.0 Flash**	**DeepSeek V3**	**Humans**	**ChatGPT**	**x.AI Grok 3**	**Gemini 2.0 Flash**	**DeepSeek V3**
Patient selection										
Was a consecutive or random sample of patients enrolled?	Yes	Yes	Yes	Yes	Yes	Unclear	No	Unclear	No	Unclear
Was a case-control design avoided?	Yes	Yes	Yes	Yes	Yes	No	No	No	No	No
Did the study avoid inappropriate exclusions?	Yes	Yes	Yes	No	Yes	Yes	Yes	Yes	No	Yes
Risk of bias assessment	Low	Low	Low	Unclear	Low	High	High	High	High	High
Index test										
Were the index test results interpreted without knowledge of the results of the reference standard?	Yes	Unclear	Yes	Yes	Unclear	Unclear	Unclear	Unclear	Unclear	Unclear
If a threshold was used, was it pre-specified?	Yes	Yes	Yes	Yes	Yes	Yes	Yes	Yes	Yes	Yes
Risk of bias assessment	Low	Unclear	Low	Low	Low	Unclear	Unclear	Unclear	Unclear	Unclear
Reference standard										
Is the reference standard likely to correctly classify the target condition?	Yes	Yes	Yes	Yes	Yes	Yes	Yes	Yes	Yes	Yes
Were the reference standard results interpreted without knowledge of the results of the index test?	Yes	Unclear	Unclear	Yes *	Unclear	Unclear	Unclear	Unclear	Unclear	Unclear
Risk of bias assessment	Low	Unclear	Unclear	Low	Low	Unclear	Unclear	Unclear	Unclear	Unclear
Flow and timing										
Was there an appropriate interval between index test(s) and reference standard?	Yes	Yes	Yes	Yes *	Yes *	Unclear	Unclear	Unclear	Unclear	Unclear
Did all patients receive a reference standard?	Yes	Yes	Yes	No	Yes	Yes	Yes	Yes	Yes	Yes
Did patients receive the same reference standard?	Yes	Yes	Yes	Yes	Yes	Yes	Yes	Yes	Yes	Yes
Were all patients included in the analysis?	Yes	Yes	No	No	Yes	No	Yes	Yes	Yes	Yes
Risk of bias assessment	Low	Low	Low	Unclear	Low	Low	Low	Low	Low	Low
**QUADAS 2 domains, signaling questions/Study**	**Vamja, 2025 [18]**					**King, 2025 [20]**				
**Assessor**	**Humans**	**ChatGPT**	**x.AI Grok 3**	**Gemini 2.0 flash**	**DeepSeek V3**	**Humans**	**ChatGPT**	**x.AI Grok 3**	**Gemini 2.0 flash**	**DeepSeek V3**
Patient selection										
Was a consecutive or random sample of patients enrolled?	Yes	Yes	Yes	Yes	Yes	Unclear	No	No	No	No
Was a case-control design avoided?	Yes	Yes	Yes	Yes	Yes	Unclear	Yes	Yes	Yes	Yes
Did the study avoid inappropriate exclusions?	Yes	Yes	No	No	Unclear	Unclear	Yes	Yes	No	Unclear
Risk of bias assessment	Low	Low	High	High	Low	Unclear	Low	High	Unclear	High
Index test										
Were the index test results interpreted without knowledge of the results of the reference standard?	Yes	Unclear	Unclear	Unclear	Unclear	Yes	Yes	Yes *	Yes *	Yes
If a threshold was used, was it pre-specified?	Yes	Yes	Yes	Yes	Yes	Yes	Yes	Yes	Yes	Yes
Risk of bias assessment	Low	Unclear	Unclear	Low	Low	Low	Low	Low	Low	Low
Reference standard										
Is the reference standard likely to correctly classify the target condition?	No	Yes	No	Yes	Yes	No	Yes	Yes	Yes	Yes
Were the reference standard results interpreted without knowledge of the results of the index test?	Unclear	Unclear	Unclear	Unclear	Unclear	Yes	Yes	Yes	Yes	No
Risk of bias assessment	High	Unclear	High	Unclear	Unclear	High	Low	Low	Low	Unclear
Flow and timing										
Was there an appropriate interval between index test(s) and reference standard?	Unclear	Yes	Unclear	Yes	Yes	Yes	Yes	Yes	Yes	Yes
Did all patients receive a reference standard?	Yes	Yes	Yes	Yes	Yes	Yes	Yes	Yes	Yes	Yes
Did patients receive the same reference standard?	Yes	Yes	Yes	Yes	Yes	Yes	Yes	Yes	Yes	Yes
Were all patients included in the analysis?	Yes	Yes	Yes	Yes	Yes	Yes	Yes	Yes	Yes	Yes
Risk of bias assessment	Low	Low	Unclear	Low	Low	Low	Low	Low	Low	Low
**QUADAS 2 domains, signaling questions/Study**	**Yin, 2025 [21]**					**Fariba, 2024 [15]**				
**Assessor**	**Humans**	**ChatGPT**	**x.AI Grok 3**	**Gemini 2.0 flash**	**DeepSeek V3**	**Humans**	**ChatGPT**	**x.AI Grok 3**	**Gemini 2.0 flash**	**DeepSeek V3**
Patient selection										
Was a consecutive or random sample of patients enrolled?	No	No	Yes	No *	No	Yes	Yes	Yes	Yes	Unclear
Was a case-control design avoided?	Yes	Yes	Yes	Yes	Yes	Yes	Yes	Yes	Yes	Yes
Did the study avoid inappropriate exclusions?	No	No	No	No	Unclear	Yes	Yes	No	No	No
Risk of bias assessment	Unclear	High	High	Unclear	Low	Low	Low	High	Unclear	High
Index test										
Were the index test results interpreted without knowledge of the results of the reference standard?	Yes	Unclear	Yes *	Yes	Yes	Yes	Unclear	Unclear	Unclear	Unclear
If a threshold was used, was it pre-specified?	No	No	Yes	No	Yes	Yes	Yes	Yes	Yes	Yes
Risk of bias assessment	High	High	Low	Unclear	Low	Low	Unclear	Unclear	Unclear	Unclear
Reference standard										
Is the reference standard likely to correctly classify the target condition?	No	No	No	No	No	Yes	Yes	Yes	Yes	Yes
Were the reference standard results interpreted without knowledge of the results of the index test?	Yes	Unclear	Yes	Yes	Unclear	Unclear	Unclear	Unclear	Unclear	Unclear
Risk of bias assessment	High	High	High	High	High	Unclear	Unclear	Unclear	Unclear	Unclear
Flow and timing										
Was there an appropriate interval between index test(s) and reference standard?	Yes	Yes	Yes	Not applicable	Not applicable	Yes	Yes	Yes	Yes	Yes
Did all patients receive a reference standard?	Yes	Yes	Yes	Yes	Yes	No	No	No	No	No
Did patients receive the same reference standard?	Yes	Yes	Yes	Yes	Yes	Yes	Yes	Yes	Yes	Yes
Were all patients included in the analysis?	Yes	No	No	No	No	No	Yes	No *	No *	Yes
Risk of bias assessment	Low	High	High	Unclear	Unclear	High	High	High	High	High
**QUADAS 2 domains, signaling questions/Study**	**Li, 2025 [16]**					**Willems, 2025 [22]**				
**Assessor**	**Humans**	**ChatGPT4o**	**x.AI Grok 3**	**Gemini 2.0 flash**	**DeepSeek V3**	**Humans**	**ChatGPT**	**x.AI Grok 3**	**Gemini 2.0 flash**	**DeepSeek V3**
Patient selection										
Was a consecutive or random sample of patients enrolled?	Unclear	Unclear	Unclear	No	Unclear	Unclear	Yes	Unclear	No	No
Was a case-control design avoided?	Yes	Yes	Yes	Yes	Yes	Yes	Yes	Yes	Yes	Yes
Did the study avoid inappropriate exclusions?	Yes	Yes	Yes	No	Yes	Yes	Yes	Yes	No	Unclear
Risk of bias assessment	Unclear	Low	Unclear	High	Low	Unclear	Low	Unclear	Unclear	High
Index test										
Were the index test results interpreted without knowledge of the results of the reference standard?	Yes	Unclear	Unclear	Yes	Yes	Yes	Yes	Yes	Yes	Yes
If a threshold was used, was it pre-specified?	No	No	No	No	No	Yes	Yes	Yes	Yes	Yes
Risk of bias assessment	High	High	High	Unclear	High	Low	Low	Low	Low	Low
Reference standard										
Is the reference standard likely to correctly classify the target condition?	No	Yes	Yes	Yes	Yes	Yes	Yes	Yes	Yes	Yes
Were the reference standard results interpreted without knowledge of the results of the index test?	Unclear	Unclear	Unclear	Yes	Unclear	Unclear	Yes	Unclear	Yes	Unclear
Risk of bias assessment	High	Unclear	Unclear	Low	Unclear	Unclear	Low	Unclear	Low	Unclear
Flow and timing										
Was there an appropriate interval between index test(s) and reference standard?	Yes	Yes	Unclear	Unclear	Yes	Yes	Yes	Yes	Yes	Yes
Did all patients receive a reference standard?	Yes	Yes	Yes	Yes	Yes	Yes	Yes	Yes	Yes	Yes
Did patients receive the same reference standard?	Yes	Yes	Yes	Yes	Yes	Yes	Yes	Yes	Yes	Yes
Were all patients included in the analysis?	Yes	Yes	Yes	Yes	Yes	No	Yes	Yes	No	No
Risk of bias assessment	Low	Low	Unclear	Unclear	Low	High	Low	Low	Unclear	High
**QUADAS 2 domains, signaling questions/Study**	**Grzybowski, 2024 [23]**					**dos Reis, 2024 [24]**				
**Assessor**	**Humans**	**ChatGPT4o**	**x.AI Grok 3**	**Gemini 2.0 flash**	**DeepSeek V3**	**Humans**	**ChatGPT**	**x.AI Grok 3**	**Gemini 2.0 flash**	**DeepSeek V3**
Patient selection										
Was a consecutive or random sample of patients enrolled?	Yes	Yes	Yes	Yes *	Unclear	Unclear	No	Yes	No	Yes
Was a case-control design avoided?	Yes	Yes	Yes	Yes	Yes	Yes	Yes	Yes	Yes	Yes
Did the study avoid inappropriate exclusions?	No	No	No	No	No	No	Yes	Yes	No	Yes
Risk of bias assessment	High	High	High	Unclear	High	High	High	Low	High	Low
Index test										
Were the index test results interpreted without knowledge of the results of the reference standard?	Yes	Yes	Yes	Yes *	Yes	Yes	Yes	Yes	Yes	Yes
If a threshold was used, was it pre-specified?	Yes	Yes	Yes	Yes	Yes	No	No	Yes	No	Yes
Risk of bias assessment	Low	Low	Low	Low	Low	Unclear	Unclear	Low	Unclear	Low
Reference standard										
Is the reference standard likely to correctly classify the target condition?	No	Yes	Yes	Yes	Yes	Yes	Yes	Yes	Yes	Yes
Were the reference standard results interpreted without knowledge of the results of the index test?	Yes	Yes	Yes	Yes	Yes	Yes	Yes *	Yes	Yes	Yes
Risk of bias assessment	High	Low	Low	Low	Low	Low	Low	Low	Low	Low
Flow and timing										
Was there an appropriate interval between index test(s) and reference standard?	Yes	Yes	Yes	Yes	Yes *	Yes	Yes	Yes	Yes	Yes
Did all patients receive a reference standard?	Yes	Yes	Yes	Yes	Yes	Yes	Yes	Yes	Yes	Yes
Did patients receive the same reference standard?	No	Yes	No	Yes	No	Yes	Yes	Yes	Yes	Yes
Were all patients included in the analysis?	No	No	No	No	No	No	Yes	No	No	Yes
Risk of bias assessment	High	High	High	Unclear	High	High	Low	High	Unclear	Low

* indicates that although the LLM gave the correct answer, the argumentation was not correct.

**Table 2:** Correct responses for signaling questions of the QUADAS-2 risk of bias tool, by large language models, overall.

Characteristic	Number (%) (n = 110)
x.AI Grok 3	85/110 (77.27)
ChatGPT4o	83/110 (75.45)
DeepSeek V3	79/110 (71.82)
Gemini 2.0 flash	74/110 (67.27)

An assessment was considered correct if both the answer was correct and the reasoning for the argument was correct.

**Table 3:** Correct responses for signaling questions of the QUADAS-2 risk of bias tool, by large language models based on domains.

Domain	Patient Selection (n = 30)	Index Test (n = 20)	Reference Standard (n = 20)	Flow and Timing (n = 40)
ChatGPT 4o, n (%)	23 (76.67)	15 (75)	12 (60)	33 (82.5)
x.AI Grok 3, n (%)	22 (73.33)	13 (65)	16 (80)	34 (85)
Gemini 2.0 flash, n (%)	15 (50)	16 (80)	13 (65)	30 (75)
DeepSeek V3, n (%)	19 (63.33)	15 (75)	13 (65)	32 (80)
Total	79 (65.83)	59 (73.75)	51 (63.75)	129 (80.63)

An assessment was considered correct if both the answer and the reasoning for the argument were correct.

## Data Availability

Data are contained within the article or Supplementary Material.

## Supplementary Materials

The following supporting information can be downloaded at https://www.mdpi.com/article/10.3390/diagnostics15121451/s1, Table S1: Human and large language models (LLMs) detailed answers for each article, as well as explanation of correct answers and LLM reasoning errors.

## Abbreviations

The following abbreviations are used in this manuscript:

AI	Artificial Intelligence
LLM	Large Language Model
QUADAS	Quality Assessment of Diagnostic Accuracy Studies
RoB	Risk of Bias
PubMed	Public/Publisher MEDLINE (medical literature database)
NHANES	National Health and Nutrition Examination Survey
ROBINS-I	Risk Of Bias In Non-randomized Studies—of Interventions
CLARITY	Clinical Advances through Research and Information Translation at McMaster University
