# Supplementary material for: Risk of Bias Assessment of Diagnostic Accuracy Studies Using QUADAS 2 by Large Language Models
Source: Diagnostics (Basel). 2025 Jun 6;15(12):1451. doi: 10.3390/diagnostics15121451 (PMC12191753; doi:10.3390/diagnostics15121451)
Supplement: Supplementary file 1 [file diagnostics-15-01451-s001.zip › diagnostics-3681099-supplementary.pdf]

**Supplementary Table S1. Human and large language models (LLMs) detailed answers for each article, as well as explanation of correct answers and LLM reasoning errors.**

| **van Kleef, 2025** | | | | |
|---|---|---|---|---|
| **Human** | **ChatGPT** | **x. AI Grok** | **Gemini 2.0 flash** | **DeepSeek V3** |
| **Patient selection** | | | | |
| Was a consecutive or random sample of patients enrolled? **Yes**<br>Was a case-control design avoided? **Yes**<br>Did the study avoid inappropriate exclusions? **Yes**<br>**Risk of bias: Low**<br>**Rationale:**<br><br>The study used the NHANES 2017-2020 data, representative to the population of USA by means of randomization sampling<br>The design was cross-sectional since data was used at one moment in time, without following the progression of the health status of participants<br>The authors excluded participants in case of incomplete data, unreliable LSM due to heart failure. Nevertheless, if those might have been different from the selected ones, this might have impacted the results. It is unlikely to be so, and the percentage of exclusions is relatively low, thus less likely to have an important impact on the results. | **Answers:**<br>Was a consecutive or random sample of patients enrolled? **Yes**<br>Was a case-control design avoided? **Yes**<br>Did the study avoid inappropriate exclusions? **Yes**<br>**Risk of bias: Low**<br>**Rationale:**<br>The study used data from NHANES 2017–2020, a nationally representative, population-based survey with randomized sampling, indicating a random sample was enrolled.<br>The design was not case-control; rather, it was a cross-sectional analysis of a general population cohort.<br>Participants were excluded only for predefined, justifiable reasons (e.g., incomplete data on LRS components, unreliable LSM due to heart failure), indicating appropriate exclusions.<br>Please proceed with the next set of signaling questions. | **Signaling Questions and Risk of Bias Assessment for Index Test Domain**<br>**Were the index test results interpreted without knowledge of the results of the reference standard?**<br>**Answer:** Yes<br>**If a threshold was used, was it pre-specified?**<br>**Answer:** Yes<br>**Risk of Bias Assessment:**<br>**Answer:** Low<br>**Rationale for Answers**<br>**Were the index test results interpreted without knowledge of the results of the reference standard?**<br>The LiverRisk Score (LRS) was calculated using an online tool (<u>www.liverriskscore.com</u>) based on predefined components (age, sex, glucose, cholesterol, AST, ALT, GGT, and platelets). The article does not explicitly state that LRS calculation was blinded to the reference standard (transient elastography, LSM). However, since the LRS is derived from objective laboratory and demographic data entered into an automated tool, the calculation process is unlikely to be influenced by knowledge of LSM results. The standardized and automated nature of the LRS calculation supports a "Yes" response, as it minimizes the risk of interpreter bias.<br>**If a threshold was used, was it pre-specified?**<br>The study used predefined LRS cut-offs (<6 for very low risk, 6-10 for low risk, 10-15 for medium risk, and >15 for high risk) to categorize participants. These cut-offs were established by the LiverScreen consortium for liver-related events, as referenced in the article, and were not determined post hoc. | Here are the answers to the signaling questions and the risk of bias assessment for the "Patient Selection" domain:<br>Was a consecutive or random sample of patients enrolled? **Yes**<br>Was a case-control design avoided? **Yes**<br>Did the study avoid inappropriate exclusions? **No**<br>Risk of bias assessment: **Unclear**<br>**Rationale:**<br>**Consecutive or random sample:** The study used data from the National Health and Nutrition Examination Survey (NHANES) 2017-2020, which is described as a population-based cohort. NHANES is designed to assess the health and nutritional status of individuals throughout the United States, using extensive interviews, physical examinations, clinical measurements, and tests by trained research assistants. This suggests a broad, representative sample rather than a selected group.<br>**Case-control design avoided:** The study design is a population-based cohort study, not a case-control study. It aims to evaluate the diagnostic accuracy of the LiverRisk Score (LRS) within this general population. | **Signaling Questions:**<br>Was a consecutive or random sample of patients enrolled?<br>**Answer:** Yes<br>**Rationale:** The study used data from the NHANES 2017-2020 cohort, which is a population-based survey designed to represent the general U.S. population. Participants were selected using a stratified, multistage probability sampling method, which approximates a random sample.<br>Was a case-control design avoided?<br>**Answer:** Yes<br>**Rationale:** The study is a cross-sectional analysis of a general population cohort, not a case-control design.<br>Did the study avoid inappropriate exclusions?<br>**Answer:** Yes<br>**Rationale:** Exclusions were limited to participants with heart failure or incomplete data for the LiverRisk Score (LRS) components, which are justified to ensure data reliability and avoid confounding.<br>**Risk of Bias:** Low |

| | | | | |
|---|---|---|---|---|
| | | The article explicitly states that these cut-offs were applied to assess the prevalence of LSM ≥8 kPa and ≥12 kPa, confirming that the thresholds were pre-specified, supporting a "Yes" response.<br>**Risk of Bias Assessment: Low**<br>The automated calculation of the LRS using an online tool reduces the likelihood of bias from knowledge of the reference standard results. Additionally, the use of pre-specified, established cut-offs ensures that the index test's thresholds were not adjusted based on the study outcomes. These factors collectively indicate a low risk of bias in the conduct and interpretation of the index test. | **Inappropriate exclusions:** The study excluded participants with incomplete data on LRS components and patients with potentially unreliable liver stiffness measurements (LSM) due to the presence of heart failure. The exclusion of patients with incomplete data is justifiable, but the exclusion of patients with heart failure, a condition that could very well be present in the population of interest, might introduce bias. It is not clearly justified why heart failure would lead to unreliable LSM.<br>**Risk of bias:** The risk of bias was rated as "unclear" due to the potential bias introduced by excluding patients with heart failure. While the study uses a population-based cohort and avoids a case-control design, the exclusion criterion might not be appropriate and its potential impact on the results is unclear. | |
| | | | **Error:** did not understand the correct exclusion due to LSM being unreliable in case of heart failure, that was actually written by the authors, and correct clinically | |
| **Index test** | | | | |
| Were the index test results interpreted without knowledge of the results of the reference standard?<br>**Yes**<br>If a threshold was used, was it pre-specified? **Yes** | **Unclear**<br>**Yes**<br>**Risk of bias: Unclear**<br>**Rationale:**<br>It is not explicitly stated whether the index test (LiverRisk Score) was interpreted without | **Were the index test results interpreted without knowledge of the results of the reference standard?**<br>**Answer:** Yes<br>**If a threshold was used, was it pre-specified?**<br>**Answer:** Yes | Here are the answers to the signaling questions and the risk of bias assessment for the "Index Test" domain:<br>Were the index test results interpreted without knowledge of the results of the reference standard? **Yes** | **Signaling Questions:**<br>Were the index test results interpreted without knowledge of the reference standard?<br>**Answer:** Unclear<br>**Rationale:** The article does not explicitly state whether the LRS was calculated blinded to the liver stiffness measurement (LSM) results. However, since the LRS was |

| | | | | |
|---|---|---|---|---|
| Risk of bias assessment **Low**<br><br>**Rationale:**<br><br>Since the LiverRisk Score is a calculation based on clinical and laboratory data it is objective and cannot be influenced by the results of the reference standard<br>The authors prespecified thresholds in the article<br>The risk of bias is thus low | knowledge of the reference standard (liver stiffness measurement), so this is unclear.<br>The thresholds for the LiverRisk Score were pre-specified (<6, 6–10, >10), as described in the methods. | **Risk of Bias Assessment:**<br>**Answer:** Low<br>**Rationale for Answers**<br>**Were the index test results interpreted without knowledge of the results of the reference standard?**<br>The LiverRisk Score (LRS) was calculated using an online tool (www.liverriskscore.com) based on predefined components (age, sex, glucose, cholesterol, AST, ALT, GGT, and platelets). The article does not explicitly state that LRS calculation was blinded to the reference standard (transient elastography, LSM). However, since the LRS is derived from objective laboratory and demographic data entered into an automated tool, the calculation process is unlikely to be influenced by knowledge of LSM results. The standardized and automated nature of the LRS calculation supports a "Yes" response, as it minimizes the risk of interpreter bias.<br>**If a threshold was used, was it pre-specified?**<br>The study used predefined LRS cut-offs (<6 for very low risk, 6-10 for low risk, 10-15 for medium risk, and >15 for high risk) to categorize participants. These cut-offs were established by the LiverScreen consortium for liver-related events, as referenced in the article, and were not determined post hoc. The article explicitly states that these cut-offs were applied to assess the prevalence of LSM ≥8 kPa and ≥12 kPa, confirming that the thresholds were pre-specified, supporting a "Yes" response.<br>**Risk of Bias Assessment: Low**<br>The automated calculation of the LRS using an online tool reduces the likelihood of bias from knowledge of the reference standard results. Additionally, the use of pre-specified, established cut-offs ensures that the index test's thresholds were not adjusted based on the study outcomes. These factors | If a threshold was used, was it pre-specified? **Yes**<br>Risk of bias assessment: **Low**<br>**Rationale:**<br>**Index test interpretation without knowledge of reference standard:** The authors calculated the LiverRisk Score (LRS) using an online tool. This calculation is based on specific variables (age, sex, glucose, cholesterol, AST, ALT, GGT, and platelets). The reference standard was the liver stiffness measurement (LSM) by transient elastography. Since the LRS is calculated using a set formula, and the LSM is a separate measurement, the interpretation of the LRS is inherently independent of the LSM result.<br>**Pre-specified threshold:** The authors state that "we calculated the diagnostic accuracy...of the LRS at the prespecified cut-offs for very low risk and low risk (6 and 10) in the detection of an LSM ≥8 and ≥12 kPa." This clearly indicates that the thresholds for the LRS were defined before the analysis.<br>**Risk of bias:** Both conditions for low risk of bias are met. The index test was interpreted independently of the reference standard, and the thresholds were pre-specified. | calculated using an online tool, it is possible that blinding was maintained.<br>If a threshold was used, was it pre-specified?<br>**Answer:** Yes<br>**Rationale:** The study used pre-specified LRS cut-offs (<6 for very low risk, 6–10 for low risk, etc.) as defined by the LiverScreen consortium.<br>**Risk of Bias:** Low |

| | | | | |
|---|---|---|---|---|
| | | collectively indicate a low risk of bias in the conduct and interpretation of the index test. | | |
| | **Error:** did not understand that the index test is objective | | | **Error:** did not understand that the index test is objective, but it suspected it would be ok since it was an online tool (although the argumentation is not solid) and stated unclear, but at the final RoB assessment it maintained the risk at 1ow |
| **Reference standard** | | | | |
| Is the reference standard likely to correctly classify the target condition? **Yes**<br>Were the reference standard results interpreted without knowledge of the results of the index test? **Yes**<br>Risk of bias assessment **Low**<br><br>**Rationale:**<br><br>The reference standard was a transient elastography with a FibroScan, which is a validated method.<br>Stricltly reading the text it is not mentioned, thus **Yes**. But, since NHANES cohort was not designed to study the diagnostic accuracy of a score and liver stiffness, it is almost impossible that they checked an unknown by then risk score. | **Yes**<br>**Unclear**<br>**Risk of bias: Unclear**<br>**Rationale:**<br>Transient elastography (LSM) is a well-validated and widely accepted noninvasive reference standard for assessing liver stiffness, so it is likely to correctly classify the target condition.<br>The article does not state whether the reference standard (LSM) was interpreted without knowledge of the LiverRisk Score results, so this remains unclear. | **Is the reference standard likely to correctly classify the target condition?**<br>**Answer:** Yes<br>**Were the reference standard results interpreted without knowledge of the results of the index test?**<br>**Answer:** Unclear<br>**Risk of Bias Assessment:**<br>**Answer:** Unclear<br>**Rationale for Answers**<br>**Is the reference standard likely to correctly classify the target condition?**<br>The reference standard used in the study is transient elastography (FibroScan model 502 V2 Touch) to measure liver stiffness (LSM), with cut-offs of ≥8 kPa and ≥12 kPa to define fibrosis. Transient elastography is a widely accepted, non-invasive method for assessing liver fibrosis, supported by clinical guidelines from EASL and AASLD, as referenced in the article. The study ensured measurement reliability by requiring at least 10 valid measurements with an interquartile range <30%, adhering to standard protocols. While liver biopsy is considered the gold standard, transient elastography is sufficiently accurate for detecting significant fibrosis (LSM ≥8 kPa) and advanced fibrosis (LSM ≥12 kPa) in a general population cohort like NHANES, where biopsy is impractical. Therefore, a "Yes" response is appropriate, as the reference standard is likely to correctly classify the target condition (liver fibrosis).<br>**Were the reference standard results interpreted without knowledge of the** | Is the reference standard likely to correctly classify the target condition? Yes<br><br>Were the reference standard results interpreted without knowledge of the results of the index test? Yes<br><br>Risk of bias assessment: Low<br><br>Rationale:<br><br>Correct classification of the target condition: Liver stiffness measurement (LSM) by transient elastography (FibroScan) is a well-established method for assessing liver fibrosis, the target condition. While it is not a perfect gold standard like a liver biopsy, it is a reliable and commonly used non-invasive reference standard.<br><br>Reference standard interpreted without knowledge of the index test: As mentioned in the "Index Test" domain assessment, the index test (LiverRisk Score) is calculated using an online tool based on specific variables, entirely separate from the transient elastography procedure used to | **Signaling Questions:**<br>Is the reference standard likely to correctly classify the target condition?<br>**Answer:** Yes<br>**Rationale:** Transient elastography (FibroScan) is a validated non-invasive method for assessing liver stiffness, and the study used standardized criteria (LSM ≥8 kPa for fibrosis, ≥12 kPa for advanced fibrosis).<br>Were the reference standard results interpreted without knowledge of the index test?<br>**Answer:** Unclear<br>**Rationale:** The article does not explicitly state whether the LSM results were interpreted blinded to the LRS. However, the NHANES protocol likely ensures independent assessments.<br>**Risk of Bias:** Low |

| | | | | |
|---|---|---|---|---|
| | | **results of the index test?**<br>The article does not explicitly state whether the transient elastography results (LSM) were interpreted without knowledge of the LiverRisk Score (LRS) results. Transient elastography is an objective measurement generated by the FibroScan device based on standardized protocols, which reduces the risk of subjective interpretation. However, there is no information on whether the personnel analyzing LSM results had access to LRS scores or other participant data during the process. In the NHANES setting, data collection is systematic, but the absence of specific details about blinding procedures for the reference standard interpretation leads to an “Unclear” response.<br>**Risk of Bias Assessment: Unclear**<br>The reliability of transient elastography as a reference standard supports its ability to correctly classify liver fibrosis, posing no significant bias in that aspect. However, the uncertainty regarding whether LSM results were interpreted without knowledge of LRS scores introduces a potential risk of bias. If interpreters had access to LRS results, it could theoretically influence the handling of borderline LSM values, although the objective nature of elastography minimizes this risk. Due to the lack of explicit evidence confirming blinding, the risk of bias for the reference standard domain is assessed as unclear. | obtain the reference standard (LSM). Therefore, the interpretation of the LSM results is independent of the LRS results.<br><br>Risk of bias: The reference standard (LSM) is likely to correctly classify the target condition, and its results were interpreted independently of the index test. Both conditions are met, indicating a low risk of bias in this domain. | |
| | **Error:** IA didn’t understand that although unclear, it wasn’t possible to be influenced | **Error:** IA didn’t understand that although unclear, it wasn’t possible to be influenced | **Error:** although the IA gave the correct answer the argumentation was not correct. | **Error:** although the IA gave the correct answer the argumentation was not correct. |
| Flow and timing | | | | |
| Was there an appropriate interval between index test(s) and reference standard? **Yes**<br>Did all patients receive a reference standard? **Yes** | **Yes**<br>**Yes**<br>**Yes**<br>**Yes**<br>**Risk of bias: Low**<br>**Rationale:** | **Was there an appropriate interval between index test(s) and reference standard?**<br>**Answer:** Yes | Here are the answers to the signaling questions and the risk of bias assessment for the "Flow and Timing" domain: | *Signaling Questions:*<br><br>**Was there an appropriate interval between the index test(s) and reference standard?**<br>**Answer:** Yes |

| | | | | |
|---|---|---|---|---|
| Did patients receive the same reference standard? **Yes**<br>Were all patients included in the analysis? **Yes**<br>Risk of bias assessment **Low**<br><br>**Rationale:**<br><br>The exact time between assessments is not stated. However NHANES performs their measurements in a single session or several ones, but close to one another. Since the disease evolves slowly, it won't induce bias.<br>Not all patients were included in the study, due to correct exclusions. But, those who remained were all assessed with the reference standard as one can deduce by the number of examined participants equal to the initial number minus the excluded ones.<br>Since there was only one reference standard mentioned, we understand that only one was used.<br>Not all patients were included in the analysis, due to correct exclusions. Since exclusions are at selection of the sample we can consider they are not necessary for analyses, and they were assessed in | The index test (LRS) and reference standard (LSM) were both performed using the same NHANES dataset and visit, implying an appropriate interval.<br>All included participants underwent liver stiffness measurement.<br>All patients received the same reference standard (transient elastography).<br>All eligible participants (n = 7,025) were included in the final analysis after predefined exclusions. | **Did all patients receive a reference standard?**<br>**Answer:** Yes<br>**Did patients receive the same reference standard?**<br>**Answer:** Yes<br>**Were all patients included in the analysis?**<br>**Answer:** No<br>**Risk of Bias Assessment:**<br>**Answer:** Low<br>**Rationale for Answers**<br>**Was there an appropriate interval between index test(s) and reference standard?**<br>The LiverRisk Score (LRS) is calculated based on laboratory and demographic data (age, sex, glucose, cholesterol, AST, ALT, GGT, and platelets), and the reference standard is transient elastography (LSM). In the NHANES 2017-2020 cohort, blood samples for laboratory measurements and transient elastography were collected during the same visit or examination period, as NHANES protocols involve comprehensive health assessments in a single session or closely timed sessions. The article does not indicate any significant delays between these measurements. Since liver fibrosis is a chronic condition that progresses slowly, the interval (likely within hours or days) is appropriate and unlikely to affect the diagnostic accuracy, supporting a "Yes" response.<br>**Did all patients receive a reference standard?**<br>The study started with 7,768 participants with LSM data from NHANES. After exclusions (186 for heart failure and 557 for incomplete LRS data), all 7,025 included participants had LSM measurements, as confirmed by the article's description of the cohort and results. Thus, all participants in the final analysis received the reference standard (transient elastography), justifying a "Yes" response. | Was there an appropriate interval between index test(s) and reference standard? **Yes**<br>Did all patients receive a reference standard? **No**<br>Did patients receive the same reference standard? **Yes**<br>Were all patients included in the analysis? **No**<br>Risk of bias assessment: **Unclear**<br>**Rationale:**<br>**Appropriate interval:** The authors do not explicitly state the time interval between the index test (LRS calculation) and the reference standard (LSM measurement). However, both tests were conducted using data from the NHANES 2017-2020 survey, implying that they were performed within a similar timeframe.<br>**All patients receive reference standard:** Not all participants received the reference standard. The study excluded 186 participants due to the presence of heart failure and 557 participants due to lack of data on LRS components.<br>**Same reference standard:** All participants included in the analysis received the same reference standard, which was the liver stiffness measurement (LSM) by transient elastography.<br>**All patients included in the analysis:** As mentioned above, not all patients were included in the final analysis. There | **Rationale:** The LiverRisk Score (LRS) components (blood tests, demographics) and liver stiffness measurement (LSM, via FibroScan) were collected during the same NHANES examination cycle (2017–2020). Since both were assessed contemporaneously, there was no clinically significant interval that could introduce bias from disease progression or regression.<br>**Did all patients receive a reference standard?**<br>**Answer:** Yes<br>**Rationale:** All 7,025 included participants underwent transient elastography (FibroScan) for LSM. Exclusions (heart failure, incomplete LRS data) were justified and did not selectively omit patients based on test results.<br>**Did patients receive the same reference standard?**<br>**Answer:** Yes<br>**Rationale:** All participants were assessed using the same method (FibroScan with M/XL probes) and standardized criteria (valid measurements: ≥10 acquisitions, IQR <30%).<br>**Were all patients included in the analysis?**<br>**Answer:** Yes<br>**Rationale:** The study explicitly states that 7,025 participants had complete data for both LRS and LSM. Exclusions (n=743) were due to heart failure or missing LRS components, not based on test outcomes, minimizing selection bias.<br><br>*Risk of Bias Assessment: Low*<br><br>**Rationale:**<br>No significant delay between index test (LRS) and reference standard (LSM).<br>All analyzed participants received the same reference standard.<br>Exclusions were protocol-driven and unlikely to bias results. |

| | | | | |
|---|---|---|---|---|
| the first domain. Thus, the answer is Yes. | | **Did patients receive the same reference standard?**<br>All participants underwent transient elastography using the FibroScan model 502 V2 Touch with M and XL probes, following a standardized protocol (at least 10 valid measurements with an interquartile range <30%). The article does not indicate variations in the reference standard applied across participants, confirming that all received the same reference standard, supporting a “Yes” response.<br>**Were all patients included in the analysis?**<br>Of the initial 7,768 participants with LSM data, 743 were excluded (186 due to heart failure and 557 due to incomplete LRS data), leaving 7,025 for analysis. While the exclusions were based on objective criteria (unreliable LSM due to heart failure and inability to calculate LRS due to missing data), not all patients initially enrolled were included in the final analysis. This results in a “No” response, as QUADAS-2 considers any exclusions from the analysis as potentially introducing bias, even if justified.<br>**Risk of Bias Assessment: Low**<br>The appropriate interval between index test and reference standard, universal application of the same reference standard, and the fact that all included participants received the reference standard minimize bias in the flow and timing domain. The exclusion of 743 participants (9.6% of the initial cohort) could theoretically introduce bias if the excluded group differed systematically from the included group. However, the exclusions were based on objective, non-arbitrary criteria related to data quality (missing LRS components) and test reliability (heart failure affecting LSM), and the article provides baseline characteristics suggesting the included cohort remains representative. Given the small proportion excluded and the justified reasons, the risk of bias is considered low, as these exclusions are | were exclusions due to heart failure and incomplete data.<br>**Risk of bias:** The risk of bias is "unclear." While the reference standard was the same for all included participants, the exclusions could introduce bias. Excluding participants with heart failure might not be appropriate, as discussed in the "Patient Selection" domain. Additionally, excluding participants with incomplete data on LRS components could also introduce bias if these exclusions were not at random. | No evidence of selective inclusion based on test results. |

| | | | | |
|---|---|---|---|---|
| | | unlikely to significantly distort the diagnostic accuracy results. | | |
| | | **Error:** the exclusion is at selection time (assessed in the first domain), not after it at analysis time. | **Error:** the exclusion is at selection time (assessed in the first domain), not after it at analysis time, and not specific for the standard test<br>Although for the time between assessments the answer is correct, the argumentation is based on NHANES period which is long and would allow for changes of disease, not on measurements within the same session. | Although for the time between assessments the answer is correct, the argumentation is based on NHANES period which is long and would allow for changes of disease, not on measurements within the same session. |

| Subramani, 2025 | | | | |
|---|---|---|---|---|
| **Human** | **ChatGPT 4o** | **x. AI Grok** | **Gemini 2.0 flash** | **DeepSeek V3** |
| **Patient selection** | | | | |
| Was a consecutive or random sample of patients enrolled? **Unclear**<br>Was a case-control design avoided? **No**<br>Did the study avoid inappropriate exclusions? **Yes**<br>**Risk of bias: Unclear**<br>**Rationale:**<br><br>The authors does not state if they taken a consecutive sample of patients.<br>The authors state they use a case control design, confirmed by having 50 patients per group.<br>The exclusion criteria are appropriate<br>Since a convenience sampling cannot be excluded the RoB is considered unclear | **Signaling Questions – Patient Selection Domain:**<br>**Was a consecutive or random sample of patients enrolled? – No**<br>**Was a case-control design avoided? – No**<br>**Did the study avoid inappropriate exclusions? – Yes**<br>**Risk of bias assessment – High**<br>**Rationale:**<br>The study used a **case-control design**, which is explicitly mentioned: "This study was designed as a case-control investigation". This design inherently carries a higher risk of bias for diagnostic accuracy studies due to the artificial separation of diseased and non-diseased groups.<br>There is no indication that the sample was **consecutive or random**. The method of patient enrollment is not described in detail, so we assume it was convenience sampling, which increases the risk of selection bias.<br>**Inappropriate exclusions** were **avoided**, as the study listed clear exclusion criteria (e.g., other neuropathies, chronic renal failure, vitamin deficiencies), which are justified and help ensure a homogeneous sample | **Signaling Questions and Answers:**<br>**Was a consecutive or random sample of patients enrolled?**<br>**Answer:** Unclear<br>**Was a case-control design avoided?**<br>**Answer:** No<br>**Did the study avoid inappropriate exclusions?**<br>**Answer:** Yes<br>**Risk of Bias Assessment:**<br>**Patient Selection:** High<br>**Rationale:**<br>**Was a consecutive or random sample of patients enrolled? (Unclear)**<br>The article does not explicitly state whether the 100 subjects (50 DPN patients and 50 healthy controls) were enrolled consecutively or randomly. It mentions that participants were from Annapoorana Medical College and Hospital, but no details are provided on the sampling method (e.g., consecutive enrollment, random selection, or convenience sampling). Without this information, it is unclear whether the selection process minimized bias.<br>**Was a case-control design avoided? (No)**<br>The study is explicitly described as a "case-control study" involving 50 patients diagnosed with diabetic peripheral neuropathy (DPN) and 50 healthy controls. Case-control designs are inherently prone to selection bias because cases and controls are selected based on the presence or absence of the condition, which can exaggerate differences between groups and affect the generalizability of diagnostic test accuracy. This increases the risk of bias.<br>**Did the study avoid inappropriate exclusions? (Yes)**<br>The study provides clear inclusion and exclusion criteria. Inclusion criteria included | Here are the answers to the signaling questions and the risk of bias assessment for the "Patient Selection" domain:<br>Was a consecutive or random sample of patients enrolled? **No**<br>Was a case-control design avoided? **No**<br>Did the study avoid inappropriate exclusions? **No**<br>Risk of bias assessment: **High**<br>**Rationale:**<br>**Was a consecutive or random sample of patients enrolled? No.** The study used a case-control design, which inherently does not involve consecutive or random sampling of all patients within a population. Cases (patients with DPN) and controls (healthy individuals) were selected based on their DPN status, not through a consecutive or random process of all patients presenting to a clinic.<br>**Was a case-control design avoided? No.** The study explicitly states it is a case-control study. Case-control studies select participants based on the outcome of interest (DPN status in this case), which is the defining characteristic of this design.<br>**Did the study avoid inappropriate exclusions? No.** The study excluded | **Signaling Questions:**<br>Was a consecutive or random sample of patients enrolled?<br>**Answer:** Unclear<br>**Rationale:** The article does not explicitly state whether the sample was consecutive or random.<br>Was a case-control design avoided?<br>**Answer:** No<br>**Rationale:** The study explicitly states it is a case-control design, which can introduce selection bias.<br>Did the study avoid inappropriate exclusions?<br>**Answer:** Yes<br>**Rationale:** The exclusion criteria (other neuropathies, chronic renal failure, etc.) are clinically justified to ensure a homogeneous sample.<br>**Risk of Bias: High**<br>**Rationale:** The case-control design inherently increases the risk of bias in patient selection. |

| | | | | |
|---|---|---|---|---|
| | relevant to the study question.<br>Please continue with the signaling questions for the next QUADAS-2 domain (Index Test) when ready. | individuals aged 30–70 years with type 2 diabetes mellitus (per WHO criteria) and a diabetes duration exceeding 3 years. Exclusion criteria ruled out individuals with other neuropathies, chronic renal failure, previous spinal injuries, vitamin deficiencies, and skin diseases. These exclusions are appropriate as they aim to ensure a homogeneous sample and reduce confounding factors that could affect the diagnosis of DPN. There is no indication of inappropriate or arbitrary exclusions.<br>**Risk of Bias Assessment: High**<br>The use of a case-control design (signaling question 2 answered "No") significantly increases the risk of bias in patient selection, as it can lead to spectrum bias and overestimation of diagnostic accuracy. The lack of clarity on whether consecutive or random sampling was used further contributes to concerns, though the appropriate exclusion criteria mitigate some risk. Per QUADAS-2 guidance, a case-control design typically results in a high risk of bias for patient selection. | individuals with other neuropathies, chronic renal failure, previous spinal injuries, vitamin deficiencies, and skin diseases to ensure a homogenous sample. While these exclusions might be clinically relevant, they can introduce bias by not reflecting the broader population of patients with diabetes in real-world settings.<br>**Risk of bias assessment: High.** The use of a case-control design, the lack of consecutive or random sampling, and the exclusions made lead to a high risk of bias. Case-control studies are susceptible to selection bias, as the selection of cases and controls can systematically differ in ways that affect the results. The exclusions further limit the generalizability of the findings to a typical diabetic population. | |
| | **Error:** No for consecutive sampling is correct only when the method of selection is clearly stated, not because stating case-control. | | **Error:** No for consecutive sampling is correct only when the method of selection is clearly stated, not because stating case-control.<br>The exclusions are appropriate | |
| **Index test** | | | | |
| **Signaling questions:**<br><br>Were the index test results interpreted without knowledge of the results of the reference standard?<br>**Unclear** | **Signaling Questions – Index Test Domain:**<br>**Were the index test results interpreted without knowledge of the results of the reference standard?** – **Unclear** | **Signaling Questions and Answers:**<br>**Were the index test results interpreted without knowledge of the results of the reference standard?**<br>**Answer:** Unclear<br>**If a threshold was used, was it pre-specified?**<br>**Answer:** Yes | Here are the answers to the signaling questions and the risk of bias assessment for the "Index Test" domain:<br>Were the index test results interpreted without knowledge | **Signaling Questions:**<br>Were the index test results interpreted without knowledge of the reference standard?<br>**Answer:** Unclear<br>**Rationale:** The article does not specify whether the assessors of the DNE score or VPT were blinded to the NCS results.<br>If a threshold was used, was it pre-specified? |

| | | | | |
|---|---|---|---|---|
| If a threshold was used, was it pre-specified? **Yes**<br>Risk of bias assessment **Low**<br><br>**Rationale:**<br><br>The authors did not clarify weather the blind approach was used<br>Both thresholds were specified<br>The unclear blinding results in unclear RoB assessments | **If a threshold was used, was it pre-specified?** – **Yes**<br>**Risk of bias assessment** – **Unclear**<br>**Rationale:**<br>The study does **not report** whether the interpretation of the index tests (DNE score and VPT) was done **blinded to the results of the reference standard (NCS)**. This lack of information leads to an **"unclear"** rating for blinding.<br>The **thresholds for the index tests were pre-specified**: the DNE score threshold was >3, and the VPT threshold was >25 mV, both of which are mentioned in the methods section and referenced appropriately.<br>Because the **blinding of index test interpretation is not reported**, and this could introduce bias, the **overall risk of bias is unclear** for the Index Test domain. | **Risk of Bias Assessment:**<br>**Index Test:** Unclear<br>**Rationale:**<br>**Were the index test results interpreted without knowledge of the results of the reference standard? (Unclear)**<br>The article does not provide explicit information on whether the index tests (Diabetic Neuropathy Examination [DNE] score and Vibration Perception Threshold [VPT]) were interpreted without knowledge of the reference standard (Nerve Conduction Studies [NCS]). Blinding of assessors to the reference standard results is critical to avoid bias in interpreting the index test outcomes. Since the study does not mention blinding procedures or the sequence of test administration, it is unclear whether this was ensured, leading to an unclear risk of bias for this question.<br>**If a threshold was used, was it pre-specified? (Yes)**<br>The study clearly defines thresholds for both index tests. For the VPT, a voltage exceeding 25 mV was indicative of neuropathy, and for the DNE score, a score above 3 indicated the presence of polyneuropathy. These thresholds are consistent with established criteria referenced in the article (e.g., [8] for VPT and [9] for DNE), suggesting they were pre-specified and not arbitrarily chosen post hoc. This reduces the risk of bias related to threshold selection.<br>**Risk of Bias Assessment: Unclear**<br>The risk of bias for the index test domain is assessed as unclear primarily due to the lack of information on whether the index test results were interpreted without knowledge of the reference standard (signaling question 1). The pre-specification of thresholds (signaling question 2) is a strength, but the absence of clarity on blinding procedures means that potential bias in test interpretation cannot be ruled out. Per | of the results of the reference standard? **Unclear**<br>If a threshold was used, was it pre-specified? **Yes**<br>Risk of bias assessment: **Unclear**<br>**Rationale:**<br>**Were the index test results interpreted without knowledge of the results of the reference standard? Unclear.** The article does not explicitly state whether the index test (VPT and DNE) results were interpreted without knowledge of the reference standard (NCS) results. This lack of clarity makes it difficult to determine if there was any potential for bias in the interpretation of the index tests.<br>**If a threshold was used, was it pre-specified? Yes.** The study does specify thresholds for both the index tests: "A voltage exceeding 25 mV was indicative of neuropathy" for VPT, and "scores above 3 indicated the presence of polyneuropathy" for the DNE score.<br>**Risk of bias assessment: Unclear.** Due to the unclear reporting on whether the index test results were interpreted blindly to the reference standard, the risk of bias for the index test domain is unclear. Lack of blinding can introduce bias, but the presence of pre-specified | **Answer:** Yes<br>**Rationale:** The thresholds for DNE (>3) and VPT (>25 mV) were clearly defined.<br>**Risk of Bias: Unclear**<br>**Rationale:** Lack of information on blinding raises concerns about potential bias in interpretation. |

| | | | | |
|---|---|---|---|---|
| | | QUADAS-2 guidance, an unclear answer to a key signaling question typically results in an unclear risk of bias unless other factors strongly indicate otherwise. | thresholds reduces the risk of manipulation of test results. | |
| | | | | |
| **Reference standard** | | | | |
| **Signaling questions:**<br><br>Is the reference standard likely to correctly classify the target condition? **Yes**<br>Were the reference standard results interpreted without knowledge of the results of the index test? **Unclear**<br>Risk of bias assessment **Unclear**<br><br>**Rationale:**<br><br>The authors used NCS, the golden standard for diagnostic of DPN<br>As for the previous question, blinding is not clearly stated<br><br>Therefore the RoB remains unclear | **Signaling Questions – Reference Standard Domain:**<br>**Is the reference standard likely to correctly classify the target condition?** – **Yes**<br>**Were the reference standard results interpreted without knowledge of the results of the index test?** – **Unclear**<br>**Risk of bias assessment** – **Unclear**<br>**Rationale:**<br>The reference standard used was **nerve conduction studies (NCS)**, which is widely recognized as the **gold standard** for diagnosing diabetic peripheral neuropathy. Therefore, it is **likely to correctly classify** the target condition.<br>The study **does not state** whether the interpretation of the NCS results was done **blinded to the index test results** (DNE score and VPT). Without explicit reporting, this aspect remains **unclear**.<br>Because the potential for **review bias** exists without reported blinding, and despite using an appropriate | **Signaling Questions and Answers:**<br>**Is the reference standard likely to correctly classify the target condition?**<br>**Answer:** Yes<br>**Were the reference standard results interpreted without knowledge of the results of the index test?**<br>**Answer:** Unclear<br>**Risk of Bias Assessment:**<br>**Reference Standard:** Unclear<br>**Rationale:**<br>**Is the reference standard likely to correctly classify the target condition? (Yes)**<br>The reference standard used in the study is Nerve Conduction Studies (NCS), which is widely recognized as the gold standard for diagnosing diabetic peripheral neuropathy (DPN). The article explicitly states that NCS is considered the gold standard due to its objectivity and reliability (page 2). NCS measures nerve function through electrical impulses, providing a direct and accurate assessment of peripheral nerve damage, which aligns with the target condition (DPN). Therefore, NCS is likely to correctly classify DPN, supporting a low risk of bias for this question.<br>**Were the reference standard results interpreted without knowledge of the results of the index test? (Unclear)**<br>The article does not provide information on whether the NCS results were interpreted without knowledge of the index test results (DNE score and VPT). Blinding of the assessors interpreting the reference standard to the index test results is essential to prevent bias, as knowledge of the index test | Here are the answers to the signaling questions and the risk of bias assessment for the "Reference Standard" domain:<br>Is the reference standard likely to correctly classify the target condition? **Yes**<br>Were the reference standard results interpreted without knowledge of the results of the index test? **Unclear**<br>Risk of bias assessment: **Unclear**<br>**Rationale:**<br>**Is the reference standard likely to correctly classify the target condition? Yes.** The study used nerve conduction studies (NCS) as the reference standard, and the article states that NCS is considered the gold standard due to its objectivity and reliability. Therefore, it is likely to correctly classify the target condition (DPN).<br>**Were the reference standard results interpreted without knowledge of the results of the index test? Unclear.** Similar to the index test, the article does not explicitly mention whether the reference standard results were interpreted without knowledge of the index test results. The lack of clear reporting | **Signaling Questions:**<br>Is the reference standard likely to correctly classify the target condition?<br>**Answer:** Yes<br>**Rationale:** NCS is considered the gold standard for diagnosing DPN.<br>Were the reference standard results interpreted without knowledge of the index test?<br>**Answer:** Unclear<br>**Rationale:** The article does not mention whether NCS assessors were blinded to DNE or VPT results.<br>**Risk of Bias: Unclear**<br>**Rationale:** Blinding procedures are not described, which could affect objectivity. |

| | | | | |
|---|---|---|---|---|
| | reference standard, the **risk of bias is rated as unclear**. | outcomes could influence the interpretation of NCS findings. Since the study does not mention blinding procedures or the sequence of test administration, it is unclear whether this was ensured, leading to an unclear risk of bias for this question.<br>**Risk of Bias Assessment: Unclear**<br>The risk of bias for the reference standard domain is assessed as unclear due to the lack of information on whether the NCS results were interpreted without knowledge of the index test results (signaling question 2). While the use of NCS as the reference standard is appropriate and likely to correctly classify DPN (signaling question 1), the absence of clarity on blinding procedures introduces uncertainty. Per QUADAS-2 guidance, an unclear answer to a key signaling question results in an unclear risk of bias unless other factors clearly indicate a high or low risk. | introduces uncertainty about potential bias.<br>**Risk of bias assessment: Unclear.** While the reference standard (NCS) is appropriate, the unclear reporting on blinding introduces potential for bias. If the reference standard results were interpreted with knowledge of the index test results, it could lead to an overestimation of the agreement between the tests. | |
| | | | | |
| Flow and timing | | | | |
| **Signaling questions:**<br><br>Was there an appropriate interval between index test(s) and reference standard? **Unclear**<br>Did all patients receive a reference standard? **Yes**<br>Did patients receive the same reference standard? **Yes**<br>Were all patients included in the analysis? **No**<br>Risk of bias assessment **Low**<br><br>**Rationale:** | **Signaling Questions – Flow and Timing Domain:**<br>**Was there an appropriate interval between index test(s) and reference standard? – Unclear**<br>**Did all patients receive a reference standard? – Yes**<br>**Did patients receive the same reference standard? – Yes**<br>**Were all patients included in the analysis? – Yes**<br>**Risk of bias assessment – Low**<br>**Rationale:**<br>The study **does not specify the time interval** between the index tests (DNE, VPT) and the reference standard | **Signaling Questions and Answers:**<br>**Was there an appropriate interval between index test(s) and reference standard?**<br>**Answer:** Unclear<br>**Did all patients receive a reference standard?**<br>**Answer:** Yes<br>**Did patients receive the same reference standard?**<br>**Answer:** Yes<br>**Were all patients included in the analysis?**<br>**Answer:** Yes<br>**Risk of Bias Assessment:**<br>**Flow and Timing:** Unclear<br>**Rationale:**<br>**Was there an appropriate interval between index test(s) and reference standard? (Unclear)**<br>The article does not provide details on the timing or interval between the administration | Here are the answers to the signaling questions and the risk of bias assessment for the "Flow and Timing" domain:<br>Was there an appropriate interval between index test(s) and reference standard? **Unclear**<br>Did all patients receive a reference standard? **Yes**<br>Did patients receive the same reference standard? **Yes**<br>Were all patients included in the analysis? **Yes**<br>Risk of bias assessment: **Unclear**<br>**Rationale:**<br>**Was there an appropriate interval between index test(s) and reference standard?** | **Signaling Questions:**<br>Was there an appropriate interval between index test and reference standard?<br>**Answer:** Unclear<br>**Rationale:** The time gap between DNE/VPT and NCS is not specified.<br>Did all patients receive the same reference standard?<br>**Answer:** Yes<br>**Rationale:** All participants were evaluated using NCS.<br>Were all patients included in the analysis?<br>**Answer:** Yes<br>**Rationale:** No mention of missing data or exclusions post-enrollment.<br>**Risk of Bias: Low**<br>**Rationale:** No evidence of attrition or differential verification bias. |

| | | | | |
|---|---|---|---|---|
| The authors did not mention the time between assessments<br>There is no mention or data suggesting that not all patients received the standard test<br>The mention only one standard test<br>There is no mention or data suggesting that not all patients were analysed. But, in Table 3 if one computes how many had the index test positive based on Sp and 50 – number of non diseased, will get 43.33. When computing 43/50=0.86, and 44/50=88, which means they had a different number of subjects. Nevertheless, it seems the difference is small.<br>While the authors did not state clearly the time between the assessments, it is highly unlikely to be a long time since it would be difficult to conduct such a study. Furthermore, the disease of interest evolves slowly, thus even if there is some time between the measurements, it is unlikely to raise the risk of bias. Thus, the RoB is low | (NCS), so the appropriateness of timing is **unclear**.<br>All participants (100 total) are reported to have undergone the reference standard test, suggesting **complete application** of the reference standard across the sample.<br>There is **no indication of differential testing**, and all patients appear to have received the **same reference standard (NCS)**.<br>The study clearly reports data for all enrolled patients, with **no exclusions from analysis**, satisfying the criterion for completeness.<br>Despite the unclear timing, the consistency in application and completeness of data justify a **low overall risk of bias** for this domain. | of the index tests (DNE score and VPT) and the reference standard (NCS). For diagnostic accuracy studies, an appropriate interval is critical to ensure that the disease state does not change between tests, which could affect results. Since the study does not specify whether the tests were conducted concurrently or within a short time frame, it is unclear whether the interval was appropriate, contributing to an unclear risk of bias for this question.<br>**Did all patients receive a reference standard? (Yes)**<br>The article indicates that the study involved 100 subjects (50 DPN patients and 50 healthy controls), and the results (Table 3) compare the index tests (VPT, DNE, and their combination) against NCS, implying that all participants underwent NCS as the reference standard. There is no mention of any participants not receiving the reference standard, supporting a low risk of bias for this question.<br>**Did patients receive the same reference standard? (Yes)**<br>The reference standard used is consistently described as NCS across the study for all participants. The article does not indicate any variation in the reference standard applied to different groups (cases or controls), confirming that all patients received the same reference standard. This reduces the risk of bias related to differential verification.<br>**Were all patients included in the analysis? (Yes)**<br>The study enrolled 100 subjects, and the results (e.g., Table 3) report diagnostic accuracy metrics (sensitivity, specificity, PPV, NPV) based on the full sample, with no mention of dropouts, withdrawals, or exclusion of any participants from the analysis. This suggests that all enrolled patients were included in the final analysis, | **Unclear.** The study does not provide information on the time interval between the index tests (VPT and DNE) and the reference standard (NCS). It is unclear if the tests were performed simultaneously or if there was a delay, which could affect the assessment of DPN.<br>**Did all patients receive a reference standard? Yes.** The study clearly states that the VPT and DNE score were validated against the NCS, implying that all participants received the reference standard.<br>**Did patients receive the same reference standard? Yes.** All patients received NCS as the reference standard.<br>**Were all patients included in the analysis? Yes.** The results and analysis include data from all 100 participants, and there is no mention of any dropouts or exclusions from the final analysis.<br><br>**Risk of bias assessment: Unclear.** The lack of information on the time interval between the index and reference tests makes it difficult to assess the risk of bias. If there was a significant delay, changes in the patient's condition could lead to discrepancies between the test results. However, since all patients received the reference standard and were included in the analysis, these aspects do not introduce bias. | |

| | | | | |
|---|---|---|---|---|
| | | supporting a low risk of bias for this question.<br>**Risk of Bias Assessment: Unclear**<br>The risk of bias for the flow and timing domain is assessed as unclear due to the lack of information on the interval between the index tests and the reference standard (signaling question 1). While the study demonstrates strengths in ensuring all patients received the same reference standard and were included in the analysis (signaling questions 2, 3, and 4), the uncertainty regarding the timing of test administration introduces potential bias. Per QUADAS-2 guidance, an unclear answer to a key signaling question typically results in an unclear risk of bias unless other factors strongly indicate a high or low risk. | | |
| | Error: The IA did not calculate from the table if participants were not analyzed | Error: The IA did not calculate from the table if participants were not analyzed | Error: The IA did not calculate from the table if participants were not analyzed | Error: The IA did not calculate from the table if participants were not analyzed |

| Vamja, 2025 | | | | |
|---|---|---|---|---|
| **Human** | **ChatGPT 4o** | **x. AI Grok 3** | **Gemini 2.0 flash** | **DeepSeek V3** |
| **Patient selection** | | | | |
| **Signaling questions:**<br>Was a consecutive or random sample of patients enrolled? **Yes**<br>Was a case-control design avoided? **Yes**<br>Did the study avoid inappropriate exclusions? **Yes**<br>**Risk of bias: Low**<br>**Rationale:**<br><br>The authors clearly stated consecutive sampling<br>By stating consecutive sampling and cross-sectional, and no other grouping, case-control can be deduced<br>The exclusions are clinically justified<br>Therefore there is a low RoB | **Answers (per your requested format):**<br>**Was a consecutive or random sample of patients enrolled?** Yes<br>**Was a case-control design avoided?** Yes<br>**Did the study avoid inappropriate exclusions?** Yes<br>**Risk of bias assessment:** Low<br>**Rationale:**<br>The study employed **consecutive sampling** of eligible adults attending the outpatient department during the study period, which meets the requirement for minimizing selection bias.<br>A **case-control design was not used**; instead, the study was described as a **cross-sectional diagnostic accuracy** study, which is appropriate for this type of diagnostic evaluation.<br>**Inclusion and exclusion criteria** were clearly defined and appropriate. Exclusions were related to confounding liver conditions (e.g., viral hepatitis, decompensated cirrhosis), which is standard and justifiable in diagnostic accuracy studies for fatty liver conditions. No indication of arbitrary or | **Answers:**<br>Was a consecutive or random sample of patients enrolled? **Yes**<br>Was a case-control design avoided? **Yes**<br>Did the study avoid inappropriate exclusions? **No**<br>**Risk of Bias: High**<br>**Signaling Question 1: Was a consecutive or random sample of patients enrolled? (Yes)**<br>The article states that "consecutive sampling was used" for recruiting 500 adults visiting the general medicine outpatient department until the target sample size was achieved. This indicates a consecutive enrollment process, satisfying this criterion.<br>**Signaling Question 2: Was a case-control design avoided? (Yes)**<br>The study is a cross-sectional diagnostic accuracy study, not a case-control design. All participants were assessed for MAFLD using both the index test (FLI) and reference standard (ultrasound) without pre-selecting cases and controls.<br>**Signaling Question 3: Did the study avoid inappropriate exclusions? (No)**<br>The study excluded patients with known liver diseases other than MAFLD, decompensated cirrhosis, hepatocellular carcinoma, heart failure, and chronic kidney disease stage 3 or higher. While some exclusions (e.g., other liver diseases) are appropriate to focus on MAFLD, excluding conditions like heart failure and chronic kidney disease may be inappropriate as these are common comorbidities in patients with metabolic syndrome, potentially leading to a non-representative sample. Additionally, the inclusion criterion requiring at least one metabolic risk factor (obesity, diabetes, hypertension, or dyslipidemia) introduces | Okay, I will answer the signaling questions for the patient selection domain.<br>**Was a consecutive or random sample of patients enrolled?**<br>Answer: Yes<br>Risk of bias: Low<br>**Was a case-control design avoided?**<br>Answer: Yes<br>Risk of bias: Low<br>**Did the study avoid inappropriate exclusions?**<br>Answer: No<br>Risk of bias: High<br>**Rationale:**<br>**Was a consecutive or random sample of patients enrolled?** The study used consecutive sampling. All eligible adults visiting the general medicine outpatient department during the study period were screened and recruited until the target sample size was achieved. Consecutive sampling, where all patients meeting the inclusion criteria within a defined period are included, is considered an acceptable method to reduce selection bias.<br>**Was a case-control design avoided?** The study employed a cross-sectional design, not a case-control study. It aimed to assess the diagnostic accuracy of FLI in a specific population, | |

| | | | | |
|---|---|---|---|---|
| | excessive exclusions was found. | selection bias, as it restricts the study to a high-risk population, which may not reflect the general population attending a tertiary care hospital. | which is appropriate for a cross-sectional study.<br>**Did the study avoid inappropriate exclusions?**<br>The study excluded several patient groups, including pregnant women, patients with known liver diseases other than MAFLD, decompensated cirrhosis, hepatocellular carcinoma, heart failure, and chronic kidney disease stage 3 or higher. While some exclusions are necessary, excluding patients with other liver diseases is appropriate, the exclusion of patients with heart failure and chronic kidney disease might be inappropriate as these conditions can be related to MAFLD. This could introduce bias by not representing the population with comorbidities. | |
| | | **Error:** the exclusions are clinically justified. Kindey disease could affect laboratory findings implicated in FLI. Chronic heart failure can affect fibroscan measurements as well as FLI components. The inclusion of metabolic diseases is justified due to the diagnosis of MAFLD | **Error:** the exclusions are clinically justified. Kindey disease could affect laboratory findings implicated in FLI. Chronic heart failure can affect fibroscan measurements as well as FLI components. | **Error:** the exclusions are clinically justified. The AI states it is not known if exclusions can induce bias. |
| **Index test** | | | | |
| **Signaling questions:**<br><br>Were the index test results interpreted without knowledge of the results of the reference standard? **yes**<br>If a threshold was used, was it pre-specified? **Yes**<br>Risk of bias assessment **low** | **Answers:**<br>**Were the index test results interpreted without knowledge of the results of the reference standard?** Unclear<br>**If a threshold was used, was it pre-specified?** Yes<br>**Risk of bias assessment:** Unclear<br>**Rationale:** | **Answers:**<br>Was the index test results interpreted without knowledge of the results of the reference standard? **Unclear**<br>If a threshold was used, was it pre-specified? **Yes**<br>**Risk of Bias: Unclear**<br>**Signaling Question 1: Was the index test results interpreted without knowledge of the results of the reference standard? (Unclear)**<br>The article does not explicitly state whether the FLI calculations were performed blinded | Okay, I will now assess the index test domain.<br>**Were the index test results interpreted without knowledge of the results of the reference standard?**<br>Answer: Unclear<br>Risk of bias: Unclear<br>**If a threshold was used, was it pre-specified?**<br>Answer: Yes<br>Risk of bias: Low<br>**Rationale:** | **Signaling Questions:**<br>Were the index test results interpreted without knowledge of the reference standard?<br>**Answer:** Unclear<br>**Rationale:** The article does not explicitly state whether FLI was calculated before or independently of ultrasound results.<br>Was a prespecified threshold used for FLI?<br>**Answer:** Yes<br>**Rationale:** The study used FLI ≥ 60 as the predefined cutoff.<br>**Risk of Bias:** Low |

| | | | | |
|---|---|---|---|---|
| **Rationale:**<br><br>Since the FLI is a calculation based on clinical and laboratory data it is objective and cannot be influenced by the results of the reference standard<br>The authors prespecified thresholds in the article<br>The risk of bias is thus low | The article **does not clearly state** whether the interpretation of the Fatty Liver Index (FLI) was done **blinded to the ultrasound results**, which introduces uncertainty about potential review bias.<br>The **FLI cutoff threshold (≥60)** used to define fatty liver was clearly **pre-specified**, as mentioned multiple times in the methodology and analysis sections.<br>Due to the **lack of clarity regarding blinding**, the risk of bias for this domain is **rated as unclear**, even though the threshold was appropriately handled. | to the ultrasound results. While FLI is a formula-based score using objective measures (BMI, waist circumference, triglycerides, GGT), there is no mention of blinding or the sequence of testing, making it unclear if knowledge of ultrasound results could have influenced FLI interpretation.<br>**Signaling Question 2: If a threshold was used, was it pre-specified? (Yes)**<br>The study uses a pre-specified FLI threshold of ≥60 to indicate fatty liver, as stated in the methods section and consistent with prior literature. The article also evaluates other thresholds (>30, >45) but clearly defines ≥60 as the primary cutoff for analysis.<br>**Risk of Bias: Unclear**<br>The lack of information on blinding of FLI interpretation to ultrasound results introduces uncertainty about potential bias in the index test application. | **Were the index test results interpreted without knowledge of the results of the reference standard?** The article does not explicitly state whether the interpretation of the Fatty Liver Index (FLI) was done without knowledge of the ultrasound results. It mentions that ultrasound examinations were performed by experienced radiologists, and FLI was calculated using a published formula, but it doesn't clarify if these were done independently. This lack of clarity makes it difficult to determine if there was any potential bias in the interpretation of the index test based on knowledge of the reference standard.<br>**If a threshold was used, was it pre-specified?** Yes, the threshold for FLI was pre-specified. The study defined FLI ≥60 as the cutoff for fatty liver, which is based on a published formula. | |
| | **Error:** IA didn't understand that although unclear, it wasn't possible to be influenced | **Error:** IA didn't understand that although unclear, it wasn't possible to be influenced | **Error:** IA didn't understand that although unclear, it wasn't possible to be influenced. Strangely, although for one question the answer was unclear, the RoB was low instead of unclear. No rationale is given | **Error:** IA didn't understand that although unclear, it wasn't possible to be influenced |
| **Reference standard** | | | | |
| **Signaling questions:**<br><br>Is the reference standard likely to correctly classify the target condition? **No** | **Answers:**<br>**Is the reference standard likely to correctly classify the target condition?** Yes<br>**Were the reference standard results** | **Signaling Questions:**<br><br>Is the reference standard likely to correctly classify the target condition? **No** | Okay, I'm ready to assess the reference standard domain.<br>**Is the reference standard likely to correctly classify the target condition?**<br>Answer: Yes | **Signaling Questions:**<br>Is the reference standard likely to correctly classify the target condition (MAFLD)?<br>**Answer:** Yes |

| | | | | |
|---|---|---|---|---|
| Were the reference standard results interpreted without knowledge of the results of the index test? **Unclear**<br>Risk of bias assessment **No**<br><br>**Rationale:**<br><br>The authors used ultrasound, for the diagnostic of MAFLD. It is not the best standard, the hepatic biopsy with histological examination. The authors even specify its imperfect sensitivity and specificity.<br>Blinding is not clearly stated<br>Due to improper standard test the RoB is High | **interpreted without knowledge of the results of the index test?** Unclear<br>**Risk of bias assessment:** Unclear<br>**Rationale:**<br>**Ultrasound** was used as the reference standard for diagnosing MAFLD. While not as accurate as liver biopsy, it is a **widely accepted, non-invasive first-line tool** for detecting hepatic steatosis in clinical and epidemiological settings. Therefore, it is **likely to correctly classify** the condition in most cases.<br>The study **does not mention whether ultrasound examiners were blinded** to the FLI results. This lack of information makes it **unclear** whether incorporation bias or review bias was avoided.<br>Given the **adequacy of the reference standard itself** but **uncertainty around blinding**, the overall **risk of bias is rated as unclear** for this domain. | Were the reference standard results interpreted without knowledge of the results of the index test? **Unclear**<br><br>**Risk of Bias: High Applicability Concerns: Low**<br><br>Rationale for Answers<br><br>**Signaling Question 1: Is the reference standard likely to correctly classify the target condition? (No)**<br>The reference standard used in the study is ultrasound, which diagnoses MAFLD based on hepatic steatosis combined with metabolic criteria (overweight/obesity, type 2 diabetes, or metabolic dysregulation). The article explicitly acknowledges that ultrasound has a sensitivity of 60–94% and specificity of 66–97%, depending on the degree of fatty infiltration, and cannot differentiate simple steatosis from metabolic-dysfunction-associated steatohepatitis (MASH) or assess fibrosis. These limitations indicate that ultrasound may misclassify some cases, particularly those with mild steatosis or advanced disease stages, as it is less accurate than the gold standard (liver biopsy). Therefore, it is not likely to correctly classify all cases of MAFLD.<br>**Signaling Question 2: Were the reference standard results interpreted without knowledge of the results of the index test? (Unclear)**<br>The article does not provide information on whether the ultrasound results were interpreted blinded to the Fatty Liver Index (FLI) scores. While it mentions that ultrasound examinations were conducted by experienced radiologists (>5 years of experience) with high inter-observer agreement (κ=0.85), there is no explicit statement about blinding or the sequence of | Risk of bias: Low<br>**Were the reference standard results interpreted without knowledge of the results of the index test?**<br>Answer: Unclear<br>Risk of bias: Unclear<br>**Rationale:**<br>**Is the reference standard likely to correctly classify the target condition?** Ultrasound is a commonly used and reasonably accurate method for diagnosing fatty liver disease. While it may not be as precise as a liver biopsy, it is a non-invasive and widely accepted reference standard in clinical practice and research for MAFLD diagnosis.<br>**Were the reference standard results interpreted without knowledge of the results of the index test?** Similar to the index test, the article does not explicitly state whether the ultrasound results were interpreted without knowledge of the FLI scores. It mentions that ultrasound examinations were performed by experienced radiologists, but it doesn't clarify if these interpretations were done independently of the FLI calculations. This lack of clarity introduces uncertainty about potential bias. | **Rationale:** Ultrasound is a widely accepted method for diagnosing hepatic steatosis, though less accurate than biopsy.<br>Were the reference standard results interpreted without knowledge of the index test?<br>**Answer:** Unclear<br>**Rationale:** The article does not specify whether radiologists were blinded to FLI results.<br>**Risk of Bias:** Unclear |

| | | | | |
|---|---|---|---|---|
| | | testing (i.e., whether FLI results were calculated before or after ultrasound). This lack of clarity makes it unclear whether knowledge of FLI scores could have influenced ultrasound interpretation.<br>**Risk of Bias: High**<br>The high risk of bias stems primarily from the limitations of ultrasound as a reference standard, which may not correctly classify all cases of MAFLD due to its variable sensitivity and specificity and inability to detect advanced disease stages. The uncertainty regarding blinding of ultrasound interpretation to FLI results further contributes to the potential for bias, as knowledge of the index test could influence the reference standard's interpretation. | | |
| | **Error:** the standard test is not the best | | **Error:** the standard test is not the best | **Error:** the standard test is not the best |
| Flow and timing | | | | |
| **Signaling questions:**<br><br>Was there an appropriate interval between index test(s) and reference standard? **Unclear**<br>Did all patients receive a reference standard? **Yes**<br>Did patients receive the same reference standard? **Yes**<br>Were all patients included in the analysis? **No**<br>Risk of bias assessment **Low**<br><br>**Rationale:**<br><br>The authors did not mention the time between assessments, but they mention a prospective cross-sectional approach, | **Answers:**<br>**Was there an appropriate interval between index test(s) and reference standard?** Yes<br>**Did all patients receive a reference standard?** Yes<br>**Did patients receive the same reference standard?** Yes<br>**Were all patients included in the analysis?** Yes<br>**Risk of bias assessment:** Low<br>**Rationale:**<br>Both the **FLI (index test)** and **ultrasound (reference standard)** were performed during the same study visit or within a short time frame as part of a **cross-sectional diagnostic evaluation**, | **Signaling Questions:**<br><br>Was there an appropriate interval between index test(s) and reference standard? **Unclear**<br>Did all patients receive a reference standard? **Yes**<br>Did patients receive the same reference standard? **Yes**<br>Were all patients included in the analysis? **Yes**<br><br>**Risk of Bias: Unclear Applicability Concerns: Low**<br><br>**Rationale for Answers**<br>**Signaling Question 1: Was there an appropriate interval between index test(s) and reference standard? (Unclear)**<br>The article does not provide specific details on the timing or interval between the index test (Fatty Liver Index, FLI, calculated using | Interogare reușită<br>Okay, I'm ready to answer the signaling questions for the flow and timing domain.<br>**Was there an appropriate interval between index test(s) and reference standard?**<br>Answer: Yes<br>Risk of bias: Low<br>**Did all patients receive a reference standard?**<br>Answer: Yes<br>Risk of bias: Low<br>**Did patients receive the same reference standard?**<br>Answer: Yes<br>Risk of bias: Low<br>**Were all patients included in the analysis?**<br>Answer: Yes<br>Risk of bias: Low<br>**Rationale:** | **Signaling Questions:**<br>Was there an appropriate interval between index test and reference standard?<br>**Answer:** Yes<br>**Rationale:** Both tests were conducted during the same study period, minimizing time-related changes.<br>Did all patients receive the same reference standard?<br>**Answer:** Yes<br>**Rationale:** All participants underwent ultrasound for MAFLD diagnosis.<br>Were all patients included in the analysis?<br>**Answer:** Yes<br>**Rationale:** The flowchart (Fig. 1) shows 500 participants were analyzed.<br>**Risk of Bias:** Low |

| | | | | |
|---|---|---|---|---|
| stating both methods being undertaken, thus the time implicitly will be relatively short, at least comparing to the time necessary to have modifications in the evolution of liver disease<br>There is no mention or data suggesting that not all patients received the standard test<br>The authors mention only one standard test<br>There is no mention or data suggesting that not all patients were analyzed.<br>While the authors did not state clearly the time between the assessments, it is highly unlikely to be a long time since it would be difficult to conduct such a study. Furthermore, the disease of interest evolves slowly, thus even if there is some time between the measurements, it is unlikely to raise the risk of bias. Thus, the RoB is low | making the interval appropriate.<br>The study reports that **all 500 participants underwent ultrasound**, and the analysis includes all participants.<br>**The same reference standard** (ultrasound) was applied consistently to all study participants.<br>There was **no evidence of exclusions after testing** or incomplete data affecting the primary diagnostic accuracy analysis. | BMI, waist circumference, triglycerides, and GGT) and the reference standard (ultrasound). While it mentions that fasting blood samples (for triglycerides and GGT) and ultrasound were part of the data collection process, there is no information on whether these were conducted simultaneously, on the same day, or over a longer period. Without this information, it is unclear whether the interval was appropriate to ensure that the disease state remained consistent between tests, potentially introducing bias if the condition changed over time.<br>**Signaling Question 2: Did all patients receive a reference standard? (Yes)**<br>The article indicates that all 500 enrolled participants underwent ultrasound examination to diagnose MAFLD, as described in the methods section. There is no mention of any participants not receiving the reference standard, confirming that all patients were assessed with ultrasound.<br>**Signaling Question 3: Did patients receive the same reference standard? (Yes)**<br>All participants received the same reference standard, which was ultrasound performed by experienced radiologists using a high-resolution B-mode ultrasonography system. The article specifies standard criteria for diagnosing fatty liver (increased echogenicity, vascular blurring, and deep attenuation) and MAFLD (hepatic steatosis plus metabolic criteria), with no indication of variations in the ultrasound protocol or equipment used.<br>**Signaling Question 4: Were all patients included in the analysis? (Yes)**<br>The study reports that all 500 enrolled participants were included in the analysis, with no mention of dropouts, withdrawals, or exclusions after enrollment. The flowchart (Fig. 1) and results tables (e.g., Tables 1–5) consistently report data for 500 participants, | **Was there an appropriate interval between index test(s) and reference standard?** The study indicates that "All measurements were taken in a single sitting by trained personnel using standardized procedures". This suggests that the index test (FLI calculation) and the reference standard (ultrasound) were performed close in time, minimizing the risk of changes in the condition between tests.<br>**Did all patients receive a reference standard?** All participants underwent both the index test (FLI) and the reference standard (ultrasound), as stated in the methods.<br>**Did patients receive the same reference standard?** All patients received ultrasonography as the reference standard for diagnosing fatty liver.<br>**Were all patients included in the analysis?** The study clearly states that the final analytic sample included all 500 participants, as shown in the flowchart (Fig. 1) and described in the results. There were no exclusions after the tests were performed, ensuring complete case analysis. | |

| | | | | |
|---|---|---|---|---|
| | | supporting that all were included in the final analysis.<br>**Risk of Bias: Unclear**<br>The risk of bias is unclear primarily due to the lack of information on the interval between the FLI calculation and ultrasound examination. If the tests were not conducted close in time, changes in the patient's condition (e.g., metabolic status or liver fat content) could affect the results, introducing bias. However, the consistent application of the same reference standard to all patients and the inclusion of all participants in the analysis reduce other potential sources of bias. | | |
| | **Error:** if taken strictly, the authors does not mention the time between tests, thus it should be considered unclear. The argument that the study is cross-sectional does not explicitly indicate the time. | **Error:** for RoB assessment the risk is low, not unclear since it is less likely the time would be long enough to pose problems | **Error:** although the AI stated all measurements were taken in a single sitting, this text was not referring to standard and index test, but for the measurements of the standard test! | **Error:** if taken strictly, the authors does not mention the time between tests, thus it should be considered unclear. |

| King, 2025 | | | | |
|---|---|---|---|---|
| **Human** | **ChatGPT 4o** | **x. AI Grok 3** | **Gemini 2.0 flash** | **DeepSeek V3** |
| **Patient selection** | | | | |
| **Signaling questions:**<br>Was a consecutive or random sample of patients enrolled? **Unclear**<br>Was a case-control design avoided? **Unclear**<br>Did the study avoid inappropriate exclusions? **unclear**<br>**Risk of bias: Unclear**<br>**Rationale:**<br><br>The authors did not clearly state consecutive or random sample, sounds more like a convenience sample. Moreover stating an achievement of 50% with without osteoarthritis does not suggest random or consecutive clearly, but doesn't imply it was not consecutive<br>The design seems case-control since the authors aimed for a 50% with/without osteoarthritis, which implies the size of the samples was not at random but controlled by the authors and thus seems case-control design. Since it is not exactly sure, we chose unclear.<br>The authors does not mention any explicit exclusion criteria. The flowchart doesn't suggest exclusion criteria. It is unclear if they used. But | **Answers to signaling questions – Domain 1: Patient Selection**<br>**Was a consecutive or random sample of patients enrolled? – No**<br>**Was a case-control design avoided? – Yes**<br>**Did the study avoid inappropriate exclusions? – Yes**<br>**Risk of bias assessment – Low**<br>**Rationale:**<br>The study **did not use a consecutive or random sampling** method. Participants were **purposefully recruited** with a target of 50% with and without self-reported knee pain, which was not consecutive or random.<br>The study **did avoid a case-control design**, as it was a **prospective diagnostic accuracy study** nested within a larger cross-sectional study, not a comparison of cases vs. controls by outcome.<br>**Inappropriate exclusions were avoided**; the study included adults ≥45 with type 2 diabetes, and only excluded based on informed consent and ability to attend an assessment.<br>While the lack of random sampling could introduce some bias, the design was | **ignaling Questions:**<br>Was a consecutive or random sample of patients enrolled? **No**<br>Was a case-control design avoided? **Yes**<br>Did the study avoid inappropriate exclusions? **Yes**<br>**Risk of Bias: High Applicability Concerns: High**<br>**Rationale for Answers**<br>**Signaling Question 1: Was a consecutive or random sample of patients enrolled? (No)**<br>The article describes a diagnostic accuracy substudy nested within a larger cross-sectional study of individuals with type 2 diabetes. Participants were purposefully recruited to achieve approximately 50% with and 50% without self-reported knee pain, as stated: "We invited a subset of these participants with and without self-reported knee pain to participate (aiming for 50% with/without)." This purposeful sampling based on knee pain status is neither consecutive nor random, as it intentionally selects participants to balance the presence and absence of knee pain rather than enrolling all eligible participants sequentially or randomly.<br>**Signaling Question 2: Was a case-control design avoided? (Yes)**<br>The study is a cross-sectional diagnostic accuracy study, not a case-control design. All 96 participants underwent the same index test (NICE criteria questionnaire) and reference standard (rheumatologist clinical assessment) to evaluate the presence of symptomatic knee osteoarthritis (OA), regardless of their knee pain status. Participants were not pre-selected as cases (with knee OA) or controls (without knee | Here are the answers to the signaling questions and the risk of bias assessment for the "Patient Selection" domain:<br>Was a consecutive or random sample of patients enrolled? **No**<br>Was a case-control design avoided? **Yes**<br>Did the study avoid inappropriate exclusions? **No**<br>Risk of bias assessment: **Unclear**<br>**Rationale:**<br>**Was a consecutive or random sample of patients enrolled? No.** The study recruited a subset of participants from a larger study, aiming for 50% with and 50% without self-reported knee pain. This is not a consecutive or random sample of all patients with type 2 diabetes.<br>**Was a case-control design avoided? Yes.** The study design was a cross-sectional diagnostic accuracy study, not a case-control study. It included participants with and without the condition of interest (knee OA) to evaluate the index test.<br>**Did the study avoid inappropriate exclusions? No.** The study only included individuals with type 2 diabetes. This exclusion may not be appropriate as it limits the generalizability of the | *Domain 1: Patient Selection*<br><br>**Signaling Questions:**<br>Was a consecutive or random sample of patients enrolled?<br>**Answer:** No<br>**Rationale:** Participants were recruited from a subset of a larger study, aiming for 50% with/without knee pain, suggesting non-consecutive or non-random enrollment.<br>Was a case-control design avoided?<br>**Answer:** Yes<br>**Rationale:** The study used a cross-sectional design, not case-control.<br>Did the study avoid inappropriate exclusions?<br>**Answer:** Unclear<br>**Rationale:** Exclusions (e.g., based on comorbidities) are not detailed, though the focus on type 2 diabetes may introduce selection bias.<br>**Risk of Bias: High** |

| | | | | |
|---|---|---|---|---|
| the way the wrote the text doesn't seem to indicate the presence of important exclusion criteria to diminish the quality of the study.<br>Concerning the RoB, the most alarming point is the text 50% in both groups that indicates a possible case-control design, but maybe they don't really proceed that way so we chose Unclear | clearly structured to evaluate diagnostic accuracy across a broad spectrum of knee pain presentations, and **there was no indication of selective exclusion that would inflate test performance**, justifying a **low risk of bias overall**. | OA) but were assessed uniformly, avoiding a case-control design.<br>**Signaling Question 3: Did the study avoid inappropriate exclusions? (Yes)**<br>The study included adults aged ≥45 years with type 2 diabetes who had at least one visit for diabetes care in the past year. The article does not mention specific exclusion criteria beyond the requirement for consent and the focus on type 2 diabetes patients. There is no indication of inappropriate exclusions, such as excluding patients based on arbitrary or biased criteria unrelated to the study's objectives. The inclusion of participants with and without knee pain and a range of comorbidities (e.g., heart disease, kidney disease) suggests that exclusions were minimal and appropriate for the study's focus on a type 2 diabetes population.<br>**Risk of Bias: High**<br>The high risk of bias is primarily due to the non-consecutive and non-random sampling method. The purposeful recruitment aiming for 50% with and 50% without knee pain introduces selection bias, as it does not reflect a natural or representative sample of the target population (individuals with type 2 diabetes attending endocrinology clinics). This selective enrollment may overestimate the prevalence of knee OA or affect the diagnostic accuracy estimates, as the sample was manipulated to include a balanced proportion of symptomatic and asymptomatic individuals.<br>**Applicability Concerns: High**<br>The study's focus on individuals with type 2 diabetes and the purposeful sampling strategy (balancing knee pain status) limits its applicability to broader populations, such as the general population or primary care settings where knee OA is diagnosed. The specific context of type 2 diabetes patients, who may have comorbidities like neuropathy or vascular disease that could influence knee symptoms, and the artificial balance of knee | findings to individuals with type 2 diabetes.<br>**Risk of bias assessment: Unclear.** There is a potential for bias due to the non-random selection of participants and the restriction to individuals with type 2 diabetes. However, the study design was not a case-control study, which reduces the risk of bias. Therefore, the overall risk of bias for patient selection is unclear. | |

| | | | | |
|---|---|---|---|---|
| | | pain status make the findings less generalizable to other clinical settings or populations without type 2 diabetes. | | |
| | **Error:** the fact IA observed 50% aim for some groups, does not preclude consecutive sampling, thus the choice should be unclear, not No. **One can have two consecutive groups, even for case controls.**<br>For case-control is unclear, since the argument of cross-sectional drops before the 50% aiming for those with and without disease. The judgement of case control cannot be based only on the statement of design, but on how they selected the participants!<br>The exclusions were not clearly mentioned, thus we cannot imply them | **Error:** the fact IA observed 50% aim for some groups, does not preclude consecutive sampling, thus the choice should be unclear, not No. One can have two consecutive groups.<br>For case-control is unclear, since the argument of cross-sectional drops before the 50% aiming for those with and without disease. **The judgement of case control cannot be based only on the statement of design, but on how they selected the participants!**<br>The exclusions were not clearly mentioned, thus we cannot imply them | **Error:** the fact IA observed 50% aim for some groups, does not preclude consecutive sampling, thus the choice should be unclear, not No. One can have two consecutive groups.<br>For case-control is unclear, since the argument of cross-sectional drops before the 50% aiming for those with and without disease. The judgement of case control cannot be based only on the statement of design, but on how they selected the participants!<br>**The AI stated the authors did not have exclusions. But not stating exclusions, does not mean the authors did not use exclusions.** | **Error:** the fact IA observed 50% aim for some groups, does not preclude consecutive sampling, thus the choice should be unclear, not No. One can have two consecutive groups.<br>For case-control is unclear, since the argument of cross-sectional drops before the 50% aiming for those with and without disease. The judgement of case control cannot be based only on the statement of design, but on how they selected the participants! |
| **Index test** | | | | |
| **Signaling questions:**<br><br>Were the index test results interpreted without knowledge of the results of the reference standard? **Yes**<br>If a threshold was used, was it pre-specified? **Yes**<br>Risk of bias assessment **Low**<br><br>**Rationale:**<br><br>The authors stated that the standard test was | **Answers to signaling questions – Domain 2: Index Test**<br>**Were the index test results interpreted without knowledge of the results of the reference standard?** – **Yes**<br>**If a threshold was used, was it pre-specified? – Yes**<br>**Risk of bias assessment** – **Low**<br>**Rationale:**<br>The index test (self-reported questionnaire assessing the NICE criteria) was | **Signaling Questions:**<br><br>Were the index test results interpreted without knowledge of the results of the reference standard? **Yes**<br>If a threshold was used, was it pre-specified? **Yes**<br><br>**Risk of Bias: Low Applicability Concerns: Low**<br><br>Rationale for Answers<br><br>**Signaling Question 1: Were the index test results interpreted without knowledge of the results of the reference standard?** | Okay, here are the answers to the signaling questions and the risk of bias assessment for the "Index Test" domain:<br>Were the index test results interpreted without knowledge of the results of the reference standard? **Yes**<br>If a threshold was used, was it pre-specified? **Yes**<br>Risk of bias assessment: **Low**<br>**Rationale:**<br>**Were the index test results interpreted without knowledge of the results of the reference standard? Yes.** | **Signaling Questions:**<br>Were the index test results interpreted without knowledge of the reference standard?<br>**Answer:** Yes<br>**Rationale:** The NICE criteria were self-reported via questionnaire before rheumatologist assessment.<br>If a threshold was used, was it pre-specified?<br>**Answer:** Yes<br>**Rationale:** The NICE criteria thresholds (age ≥45, activity-related pain, stiffness ≤30 minutes) were predefined.<br>**Risk of Bias: Low** |

| | | | | |
|---|---|---|---|---|
| performed immediately after completing the index test, thus it was blinded<br>The authors prespecified the criteria for the index test<br>The final RoB is thus low | **completed by participants before** the clinical assessment by the rheumatologist, who was **blinded to the index test responses**.<br>The thresholds for the index test—**activity-related joint pain** and **morning stiffness ≤30 minutes**—were **clearly defined and based on the pre-specified NICE clinical criteria**.<br>Since both blinding and pre-specification were explicitly followed, **the risk of bias in this domain is low**. | **(Yes)**<br>The article states that participants completed a self-administered paper questionnaire to assess the NICE criteria (index test) immediately before the rheumatologist's clinical assessment (reference standard). It also specifies that the rheumatologist conducting the reference standard assessment was "blinded to participant responses to the index test." While the article does not explicitly state that the index test results were interpreted without knowledge of the reference standard, the self-administered nature of the questionnaire and the blinding of the rheumatologist suggest that the index test results (based on participant responses) were collected and recorded independently of the reference standard. The questionnaire responses were not interpreted by the rheumatologist performing the clinical assessment, ensuring that the index test results were not influenced by the reference standard.<br>**Signaling Question 2: If a threshold was used, was it pre-specified? (Yes)**<br>The NICE criteria for knee OA have a pre-specified threshold, defined as the presence of activity-related knee joint pain and either no morning joint stiffness or morning stiffness lasting ≤30 minutes in individuals aged ≥45 years. The article clearly outlines these criteria in the methods section and uses them consistently throughout the study. Additionally, the modified NICE criteria (omitting the stiffness criterion) are also pre-specified as a secondary analysis. The use of these established, literature-based thresholds indicates that they were pre-specified before data collection.<br>**Risk of Bias: Low**<br>The low risk of bias is supported by the blinding of the rheumatologist to the index test results and the self-administered nature of the questionnaire, which minimizes the potential for the reference standard to | The text states that "An experienced rheumatologist (LKK), blinded to participant responses to the index test..." conducted the clinical assessment (reference standard). This indicates that the index test results were interpreted without knowledge of the reference standard results.<br>**If a threshold was used, was it pre-specified? Yes.** The NICE criteria, which serve as the threshold for the index test, were clearly defined in the Methods section, indicating they were pre-specified. The study also mentions the use of the modified NICE criteria.<br>**Risk of bias assessment: Low.** The interpretation of the index test was blinded to the reference standard, and the threshold was pre-specified. | |

| | | | | |
|---|---|---|---|---|
| | | influence the index test interpretation. The pre-specified thresholds for both the NICE and modified NICE criteria further reduce the risk of bias, as the criteria for a positive test result were clearly defined and not adjusted post hoc.<br>**Applicability Concerns: Low**<br>The NICE criteria, as applied in the study, are simple, self-reported measures designed to diagnose knee OA without requiring physical examination or imaging, aligning with their intended use in clinical practice. The study evaluates these criteria in a manner consistent with their purpose, making the index test highly applicable to the question of diagnosing knee OA in a clinical setting, particularly for non-musculoskeletal specialists or in resource-limited environments. | | |
| | | **Error: the rationale does not use the precedence of the test as the argument, but in indirect implication which is not correct.** | **Error: the rationale does not use the precedence of the test as the argument, states that if the physician who performed the standard test did not know the result of the index, then this means the inverse is true – which clearly is not guaranteed.** | |
| **Reference standard** | | | | |
| **Signaling questions:**<br><br>Is the reference standard likely to correctly classify the target condition? **No**<br>Were the reference standard results interpreted without knowledge of the results of the index test? **yes**<br>Risk of bias assessment **Unclear**<br><br>**Rationale:** | **Answers to signaling questions – Domain 3: Reference Standard**<br>**Is the reference standard likely to correctly classify the target condition?** – **Yes**<br>**Were the reference standard results interpreted without knowledge of the results of the index test?** – **Yes**<br>**Risk of bias assessment** – **Low**<br>**Rationale:** | **Signaling Questions:**<br>Is the reference standard likely to correctly classify the target condition? **Yes**<br>Were the reference standard results interpreted without knowledge of the results of the index test? **Yes**<br>**Risk of Bias: Low Applicability Concerns: Low**<br>**Rationale for Answers**<br>**Signaling Question 1: Is the reference standard likely to correctly classify the target condition? (Yes)**<br>The reference standard is a clinical assessment by an experienced rheumatologist, which includes a standardized clinical history (e.g., knee joint | Here are the answers to the signaling questions and the risk of bias assessment for the "Reference Standard" domain:<br>Is the reference standard likely to correctly classify the target condition? **Yes**<br>Were the reference standard results interpreted without knowledge of the results of the index test? **Yes**<br>Risk of bias assessment: **Low**<br>**Rationale:**<br>**Is the reference standard likely to correctly classify the** | **Signaling Questions:**<br>Is the reference standard likely to correctly classify the target condition?<br>**Answer:** Yes<br>**Rationale:** Rheumatologist clinical assessment is an accepted gold standard for knee OA diagnosis.<br>Were the reference standard results interpreted without knowledge of the index test?<br>**Answer:** No<br>**Rationale:** The rheumatologist was blinded to questionnaire responses but aware of the study purpose, potentially introducing bias.<br>**Risk of Bias: Unclear** |

| | | | | |
|---|---|---|---|---|
| The authors used clinical history and physical examination who have relatively low sensitivity and specificity compared to imaging<br>It is clearly stated that the reference result was interpreted without the knowledge of the index test<br>Due to the standard test choice RoB remains High | The reference standard was a **standardized clinical assessment by an experienced rheumatologist**, using clinical history and physical exam findings, which aligns with the **accepted gold standard for diagnosing symptomatic knee OA**.<br>The rheumatologist conducting the reference standard was **blinded to the index test results**, although they were aware of the study's purpose.<br>Given the use of a recognized clinical reference and proper blinding, the **risk of bias is considered low**. | symptoms, onset/course) and physical examination (e.g., joint-line tenderness, stress pain, limitation in flexion/extension) to diagnose symptomatic knee osteoarthritis (OA). The article notes that this approach is the preferred method for diagnosing knee OA, as it integrates clinical findings without relying on imaging, which aligns with clinical guidelines. The rheumatologist's assessment categorized participants as having established OA, no OA, or possible OA, reflecting real-world diagnostic practice. The high interrater reliability (weighted κ = 0.86, 95% CI 0.60–1.00) from duplicate assessments by a second rheumatologist further supports the consistency and accuracy of the reference standard in classifying the target condition. Thus, the rheumatologist's clinical assessment is likely to correctly classify symptomatic knee OA.<br>**Signaling Question 2: Were the reference standard results interpreted without knowledge of the results of the index test? (Yes)**<br>The article explicitly states that the rheumatologist conducting the clinical assessment was "blinded to participant responses to the index test" (the NICE criteria questionnaire). This indicates that the reference standard results were interpreted without knowledge of the index test results, minimizing the risk of bias from the index test influencing the rheumatologist's diagnosis.<br>**Risk of Bias: Low**<br>The low risk of bias is supported by the use of a robust reference standard (rheumatologist clinical assessment) that is likely to correctly classify symptomatic knee OA, as evidenced by its alignment with clinical practice and high interrater reliability. The blinding of the rheumatologist to the index test results further ensures that the reference standard | **target condition? Yes.** The reference standard was a rheumatologist's clinical assessment, which is considered an appropriate method for diagnosing knee OA. The text mentions that "rheumatologist assessment of knee OA as the reference standard given that clinical history and physical examination is the preferred way to make a diagnosis of knee OA and that rheumatologists are specialists in the diagnosis and management of arthritis."<br>**Were the reference standard results interpreted without knowledge of the results of the index test? Yes.** As mentioned before, the rheumatologist was blinded to the participant's responses to the index test.<br>**Risk of bias assessment: Low.** The reference standard is appropriate and was interpreted without knowledge of the index test result. | |

| | | | | |
|---|---|---|---|---|
| | | was applied independently, reducing the potential for bias.<br>**Applicability Concerns: Low**<br>The reference standard of rheumatologist clinical assessment is highly applicable to the study question, as it reflects the preferred clinical approach for diagnosing knee OA in practice, particularly in settings where specialist evaluation is available. The use of a standardized assessment by an experienced rheumatologist aligns with the goal of evaluating the NICE criteria's accuracy against a clinically relevant benchmark, making it appropriate for the study's context. | | |
| | **Error: the literature shows the limits of the absence of imaging methods to diagnose the disease as a major diagnostic issue, while the AI argues this is the standard. Probably the AI considers the ubiquity of this examination a reason for being standard, but being a standard test is decided by the capacity to accurately observe the disease.** | **Error: the AI argues that the authors used standardized clinical history – which is not an argument for the best standard test, but for the specific method of clinical history. Moreover, the AI observes the authors state that this is a preferred method to diagnose the disease, and that it aligns with clinical guidelines. But preferred methods, or guidelines does not classify gold standards for research. The high intrarater kappa is also used as an argument, but this does not measure accuracy, which is very important in identifying a gold standard.** | **Error: the AI observes the authors state that this is a preferred method to diagnose the disease. But preferred methods does not classify gold standards for research.** | **Error: the AI states the used standard is the golden standard. But it wrongly understands the use of a method for clinical purposes, compared to an evidence based assessment of diagnostic accuracy. The AI stated that although the radiologist was blinded to the index test, wasn't blinded to the aim of the study, and thus there is bias. Indeed, there is a bias, but another type of bias different than the one that should have been assessed.** |
| Flow and timing | | | | |
| **Signaling questions:**<br><br>Was there an appropriate interval between index test(s) and reference standard? **Yes**<br>Did all patients receive a reference standard? **Yes**<br>Did patients receive the same reference standard? **Yes**<br>Were all patients included in the analysis? **Yes** | **Answers to signaling questions – Domain 4: Flow and Timing**<br>**Was there an appropriate interval between index test(s) and reference standard? – Yes**<br>**Did all patients receive a reference standard? – Yes**<br>**Did patients receive the same reference standard? – Yes**<br>**Were all patients included in the analysis? – Yes** | **Signaling Questions:**<br><br>Was there an appropriate interval between index test(s) and reference standard? **Yes**<br>Did all patients receive a reference standard? **Yes**<br>Did patients receive the same reference standard? **Yes**<br>Were all patients included in the analysis? **Yes**<br><br>**Risk of Bias: Low Applicability Concerns: Low** | Here are the answers to the signaling questions and the risk of bias assessment for the "Flow and Timing" domain:<br>Was there an appropriate interval between index test(s) and reference standard? **Yes**<br>Did all patients receive a reference standard? **Yes**<br>Did patients receive the same reference standard? **Yes**<br>Were all patients included in the analysis? **Yes**<br>Risk of bias assessment: **Low**<br>**Rationale:** | **Signaling Questions:**<br>Was there an appropriate interval between index test and reference standard?<br>**Answer:** Yes<br>**Rationale:** Assessments were near-simultaneous, minimizing disease status changes.<br>Did all patients receive the same reference standard?<br>**Answer:** Yes<br>**Rationale:** All underwent rheumatologist assessment.<br>Were all patients included in the analysis?<br>**Answer:** Yes<br>**Rationale:** All 96 participants were analyzed. |

| | | | | |
|---|---|---|---|---|
| Risk of bias assessment<br>**Low**<br><br>**Rationale:**<br><br>The authors did mention the time between assessments was during the same visit<br>They mention all patients received the standard test<br>They mention only one standard test<br>There is no mention or data suggesting that not all patients were analysed. But, in Table 3 if one computes how many had the index test positive based on Sp and 50 – number of non diseased, will get 43.33. When computing 43/50=0.86, and 44/50=88, which means they had a different number of subjects. Nevertheless, it seems the difference is small.<br>While the authors did not state clearly the time between the assessments, it is highly unlikely to be a long time since it would be difficult to conduct such a study. Furthermore, the disease of interest evolves slowly, thus even if there is some time between the measurements, it is unlikely to raise the risk of bias. Thus, the RoB is low | **Risk of bias assessment** – **Low**<br>**Rationale:**<br>The index test and reference standard were administered in **immediate succession during a single outpatient visit**, minimizing the chance of change in disease status.<br>**All 96 participants** underwent the reference standard assessment.<br>The **same reference standard** (rheumatologist clinical assessment) was applied uniformly to all participants.<br>There were **no exclusions from analysis**, and all participants were included in the accuracy calculations. | Rationale for Answers<br><br>**Signaling Question 1: Was there an appropriate interval between index test(s) and reference standard? (Yes)**<br>The article states that participants completed the self-administered questionnaire evaluating the NICE criteria (index test) "immediately followed by clinical evaluation by a rheumatologist (reference standard)." This near-simultaneous assessment ensures that the interval between the index test and reference standard is minimal, reducing the likelihood of changes in the participant's disease status (e.g., knee OA symptoms) that could affect diagnostic accuracy. The short interval is appropriate for a condition like knee OA, which is chronic and unlikely to change significantly within minutes.<br>**Signaling Question 2: Did all patients receive a reference standard? (Yes)**<br>The article confirms that all 96 participants who consented to the substudy underwent both the index test (NICE criteria questionnaire) and the reference standard (rheumatologist clinical assessment). The flowchart (Figure 1) and results section indicate that all enrolled participants completed both assessments, with no mention of any participants not receiving the reference standard.<br>**Signaling Question 3: Did patients receive the same reference standard? (Yes)**<br>All participants received the same reference standard, which was a standardized clinical assessment by an experienced rheumatologist to diagnose symptomatic knee OA. The article describes the assessment as involving a consistent approach (clinical history and physical examination) applied to all participants, with no indication of variations in the protocol or different reference standards used. The high interrater reliability (weighted κ = 0.86) | **Was there an appropriate interval between index test(s) and reference standard? Yes.** The study states, "We invited consenting participants to attend a hospital outpatient appointment where they completed a self-administered questionnaire evaluating the NICE criteria (index test) immediately followed by clinical evaluation by a rheumatologist (reference standard). The near simultaneous assessment was to avoid biases caused by changes in participant disease status." This indicates a short and appropriate interval.<br>**Did all patients receive a reference standard? Yes.** The flow chart in Figure 1 shows that all participants attending the single clinic visit underwent both the index test and the reference standard assessment.<br>**Did patients receive the same reference standard? Yes.** All participants received the rheumatologist's clinical evaluation as the reference standard.<br>**Were all patients included in the analysis? Yes.** The study clearly states, "All 96 participants were included in our primary analyses."<br>**Risk of bias assessment: Low.** The interval between tests was appropriate, all patients received the same | **Risk of Bias: Low** |

| | | | | |
|---|---|---|---|---|
| | | from duplicate assessments further supports the consistency of the reference standard.<br>**Signaling Question 4: Were all patients included in the analysis? (Yes)**<br>The article explicitly states that all 96 participants were included in the primary analyses, as shown in the flowchart (Figure 1) and results tables (e.g., Tables 1–3). There is no mention of dropouts, withdrawals, or exclusions after enrollment, confirming that all participants who underwent the index test and reference standard were included in the final analysis.<br>**Risk of Bias: Low**<br>The low risk of bias is supported by the near-simultaneous administration of the index test and reference standard, which minimizes the risk of disease progression or symptom changes affecting the results. Additionally, all participants received the same reference standard, and all were included in the analysis, ensuring no bias from differential testing or participant exclusion. | reference standard, and all were included in the analysis. | |
| | | | | |

| Yin, 2025 | | | | |
|---|---|---|---|---|
| **Human** | **ChatGPT 4o** | **x. AI Grok 3** | **Gemini 2.0 flash** | **DeepSeek V3** |
| **Patient selection** | | | | |
| **Signaling questions:**<br>Was a consecutive or random sample of patients enrolled? **No**<br>Was a case-control design avoided? **Yes**<br>Did the study avoid inappropriate exclusions? **No**<br>**Risk of bias: unclear**<br>**Rationale:**<br><br>The sample was not random, but due to lots of exclusions (more than half of the participants), especially those involving availability of variables, that are not linked to specific diagnostic criteria, spurious selection bias can be induced, it cannot be considered representative, nor consecutive.<br>There was no indication of case-control design<br>As stated above there were lots of exclusions (more than half of the participants), especially those involving availability of variables, that are not linked to specific diagnostic criteria<br>Since the exclusions effect cannot be clearly quantified, the RoB is unclear. | **Signaling Questions – Patient Selection Domain:**<br>**Was a consecutive or random sample of patients enrolled?**<br>**Answer:** No<br>**Was a case-control design avoided?**<br>**Answer:** Yes<br>**Did the study avoid inappropriate exclusions?**<br>**Answer:** No<br>**Risk of Bias – Patient Selection Domain:**<br>**Answer:** High<br>**Rationale:**<br>**Sampling**: The study used data from the NHANES survey, which is a complex, multistage, stratified probability sample of the U.S. population. However, within this dataset, the final analytical sample was not a random or consecutive cohort of patients—it was selected based on the availability of specific variables (e.g., complete sleep questionnaire data, absence of pregnancy, plausible caloric intake). This indicates a selection process not representative of the target population for diagnostic accuracy, thus failing the first criterion.<br>**Design**: The study did not use a case-control design; rather, it was a cross-sectional analysis of a population-based cohort. This appropriately avoids the inflated diagnostic | *Signaling Questions*<br><br>**Was a consecutive or random sample of patients enrolled?**<br>**Answer**: Yes<br>**Was a case-control design avoided?**<br>**Answer**: Yes<br>**Did the study avoid inappropriate exclusions?**<br>**Answer**: No<br><br>*Risk of Bias Assessment*<br><br>**Risk of Bias**: High<br><br>*Rationale*<br><br>**Was a consecutive or random sample of patients enrolled? (Yes)**<br>The study used data from the National Health and Nutrition Examination Survey (NHANES) for the cycles 2005–2008 and 2015–2018. NHANES employs a complex, multistage probability sampling design to select participants representative of the U.S. noninstitutionalized population. This approach ensures a random sample, satisfying the criterion for consecutive or random enrollment.<br>**Was a case-control design avoided? (Yes)**<br>The study is a cross-sectional analysis, not a case-control study. Participants were not selected based on their OSA status (cases vs. controls) but were drawn from the general NHANES population, with OSA status determined post-enrollment based on questionnaire responses. This design avoids the biases associated with case-control studies, such as selection bias due to predefined case and control groups.<br>**Did the study avoid inappropriate exclusions? (No)**<br>The study applied several exclusion criteria, including excluding pregnant women (N=521), participants with abnormal energy intake (<500 kcal or ≥5000 kcal per day, N=248), those lacking an OSA diagnosis or | Here are the answers to the signaling questions for the Patient Selection domain:<br>Was a consecutive or random sample of patients enrolled? **No**<br>Was a case-control design avoided? **Yes**<br>Did the study avoid inappropriate exclusions? **No**<br>Risk of bias assessment: **Unclear**<br><br>Here's the rationale for the Patient Selection domain assessment:<br>**Was a consecutive or random sample of patients enrolled? No**<br>The study used data from the NHANES database, which involves a complex, stratified sampling design. While the NHANES aims to be representative of the U.S. population, it is not strictly a consecutive or random sample of patients seeking care for OSA. The study did not enroll patients based on seeking treatment or diagnosis of OSA, but rather used a national health survey.<br>**Was a case-control design avoided? Yes**<br>The study is a cross-sectional analysis of data from a national survey. It examines the association between insulin resistance and OSA within a defined population at a specific time. It is not a case-control study, which would involve comparing a group with OSA to a group without OSA to identify risk factors.<br>**Did the study avoid inappropriate exclusions? No** | **Domain 1: Patient Selection**<br>**Signaling Question 1:** Was a consecutive or random sample of patients enrolled?<br>**Answer:** No<br>**Rationale:** The study used data from NHANES, which is a cross-sectional survey, but the selection process involved exclusions based on specific criteria (e.g., pregnancy, missing data), so it was not strictly consecutive or random.<br>**Signaling Question 2:** Was a case-control design avoided?<br>**Answer:** Yes<br>**Rationale:** The study is cross-sectional, not case-control.<br>**Signaling Question 3:** Did the study avoid inappropriate exclusions?<br>**Answer:** Unclear<br>**Rationale:** The exclusions (e.g., pregnant women, missing data) seem reasonable, but the impact of these exclusions on the generalizability is not fully discussed.<br>**Risk of Bias:** Low<br>**Rationale:** Despite some exclusions, the study population is broadly representative of the U.S. noninstitutionalized population, and the design avoids major selection biases. |

| | | | | |
|---|---|---|---|---|
| | accuracy risks associated with case-control designs.<br>**Exclusions**: There were substantial exclusions, including individuals with missing data on key variables like HDL-C, TG, BMI, and fasting insulin, as well as those lacking OSA diagnostic criteria. Such exclusions could introduce selection bias and limit the generalizability of findings, especially since more than half of the initially eligible adults were excluded (over 12,000 due to missing lab values alone). | missing data on key variables (TG, HDL-C, BMI, FINS, N=12,587), and those with incomplete covariate data or sample weights (N=540). While excluding pregnant women and those with extreme energy intake is reasonable due to potential physiological differences or data inaccuracies, the exclusion of 12,577 participants for missing data on key variables (e.g., TG, HDL-C, BMI, FINS) is substantial and could introduce selection bias. These variables are critical for calculating METS-IR and other IR indices, but their absence may be related to systematic factors (e.g., non-response bias or differences in health status), potentially skewing the study population. The large number of exclusions for missing data raises concerns about the representativeness of the final cohort (8,306 out of 39,722 initial participants).<br>**Risk of Bias: High**<br>The high risk of bias is primarily due to the significant number of exclusions for missing data, which may have resulted in a non-representative sample. Although the study used a random sampling method and avoided a case-control design, the extensive exclusions could bias the association between METS-IR and OSA, particularly if excluded participants differed systematically from those included in terms of metabolic or sleep characteristics. | The study had several exclusion criteria, including excluding participants based on pregnancy, abnormal energy intake, missing data on OSA, and missing data on key variables (HDL-C, TG, BMI, FINS) and incomplete covariate data. These exclusions resulted in a substantial reduction in sample size from the initial 39,722 individuals to a final sample of 8,306. The large number of exclusions could introduce bias if the excluded participants differed significantly from those included in terms of the relationship between insulin resistance and OSA.<br>**Risk of bias assessment: Unclear**<br>There is a risk of bias because the study did not use a consecutive or random sample, and there were a large number of exclusions. | |
| | | **Error: The AI observed the reported randomized design, but failed to take into account the exclusions of many subjects based on absence of data for several variables that will determine a selection bias, undermining the randomized design.**<br>**Setting RoB to high due to identified problems concerning the signalling questions, in case when the impact of the problems is not quantifiable in effect on the risk of the disease can be problematic.** | **Error: The AI observed that the sampled population is national, and not one concerning patients seeking diagnosis for the disease of interest, and concluded the sample is not representative. In reality the problem identified is not related to the question.**<br>**Setting RoB to high due to identified problems concerning the signalling questions, in case when the impact of the problems is not quantifiable in effect on the risk of the disease can be problematic.** | **Error: The AI argues that since the impact of exclusions is not discussed by the authors it implies it is an unclear risk of bias. Instead the AI should have used the medical and scientific rationale to identify whether their impact is important.** |
| **Index test** | | | | |

| | | | | |
|---|---|---|---|---|
| **Signaling questions:**<br><br>Were the index test results interpreted without knowledge of the results of the reference standard? **yes**<br>If a threshold was used, was it pre-specified? **No**<br>Risk of bias assessment **High**<br><br>**Rationale:**<br><br>When undertaking NHANES study, they didn't aimed specifically at obstructive sleep apnea and at the risk indices thus the assessment was double blind<br>The threshold wasn't pre-specified, but calculated within the study<br>Due to the absence of threshold the RoB is high since it is variable for each study. | **Signaling Questions – Index Test Domain:**<br>**Were the index test results interpreted without knowledge of the results of the reference standard?**<br>**Answer:** Unclear<br>**If a threshold was used, was it pre-specified?**<br>**Answer:** No<br>**Risk of Bias – Index Test Domain:**<br>**Answer:** High<br>**Rationale:**<br>**Blinding**: The study does not state whether the index test results (METS-IR and other IR indices) were interpreted independently of the reference standard (OSA risk classification via questionnaire). Given the retrospective use of NHANES data and the analytical design, it's unclear if the analyses were blinded. Lack of clarity on this aspect warrants an "unclear" rating.<br>**Threshold Pre-specification**: While the METS-IR and other indices were calculated using standard formulas, the cut-offs (tertiles or continuous variable interpretations) used for the diagnostic performance analysis (e.g., ROC curve) were determined during the study and not pre-specified in a protocol or methods section. This data-driven approach raises risk of bias. | *Signaling Questions*<br><br>**Were the index test results interpreted without knowledge of the results of the reference standard?**<br>**Answer**: Yes<br>**If a threshold was used, was it pre-specified?**<br>**Answer**: Yes<br><br>*Risk of Bias Assessment*<br><br>**Risk of Bias**: Low<br><br>*Rationale*<br><br>**Were the index test results interpreted without knowledge of the results of the reference standard? (Yes)**<br>The index test in this study is the Metabolic Score for Insulin Resistance (METS-IR), calculated using the formula METS-IR = (Ln[(2*FBG)+TG]*BMI)/[Ln(HDL-C)], based on fasting blood glucose (FBG), triglycerides (TG), high-density lipoprotein cholesterol (HDL-C), and body mass index (BMI). These measurements were obtained from NHANES laboratory and anthropometric data, which are objective and collected systematically as part of the survey protocol. The reference standard for OSA was determined using a sleep questionnaire assessing snoring, daytime sleepiness, and apnea episodes. Since METS-IR is a calculated metric derived from biochemical and anthropometric data, its interpretation (or calculation) is independent of the questionnaire-based OSA assessment. There is no indication that the researchers calculating METS-IR had access to or were influenced by the OSA questionnaire results, ensuring blinding of the index test interpretation.<br>**If a threshold was used, was it pre-specified? (Yes)**<br>The study analyzed METS-IR both as a continuous variable and in tertiles (T1: ≤35.73, T2: 35.73–45.04, T3: >45.04). The use of tertiles implies thresholds, which were determined based on the distribution of METS-IR values in the study population. While the exact cutoffs were data-driven (i.e., based on tertiles of the sample), the decision to use tertiles as a | Here are the answers to the signaling questions for the Index Test domain:<br>Were the index test results interpreted without knowledge of the results of the reference standard? **Not Applicable**<br>If a threshold was used, was it pre-specified? **No**<br>Risk of bias assessment: **Unclear**<br><br>Interogare reușită<br>Here's the rationale for the Index Test domain assessment:<br>**Were the index test results interpreted without knowledge of the results of the reference standard? Not Applicable**<br>In this study, the "index tests" are the calculations of METS-IR, TyG index, TG/HDL-C ratio, and HOMA-IR, all of which are derived from laboratory measurements (FBG, TG, HDL-C, and FINS) and BMI. There is no separate "interpretation" of these tests in the way that a clinician might interpret an image or a physical exam. The values are directly calculated from the data. Therefore, the concept of interpreting the index test "without knowledge" of the reference standard doesn't apply in this context.<br>**If a threshold was used, was it pre-specified? No**<br>The analysis used tertiles of the index tests (METS-IR, TyG index, TG/HDL-C, and HOMA-IR) to compare the prevalence of OSA across groups. While tertiles are a common way to categorize continuous variables, the specific cut-off values for these tertiles were | **Signaling Question 1:** Were the index test results interpreted without knowledge of the reference standard?<br>**Answer:** Yes<br>**Rationale:** The index tests (METS-IR, HOMA-IR, etc.) are calculated from objective laboratory measures (FBG, TG, HDL-C, BMI), independent of OSA diagnosis.<br>**Signaling Question 2:** If a threshold was used, was it prespecified?<br>**Answer:** Yes<br>**Rationale:** The tertiles for METS-IR and other indices were predefined, and the formulas for these indices are standardized.<br>**Risk of Bias:** Low<br>**Rationale:** The index tests were objectively calculated, and thresholds were prespecified. |

| | | | | |
|---|---|---|---|---|
| | | categorization method was pre-specified in the study's analytical approach, as is common in epidemiological studies to explore dose-response relationships. The tertile thresholds were not arbitrarily chosen post-hoc but were derived systematically from the data, supporting the pre-specification of the analytical method. Additionally, the study's primary analysis included METS-IR as a continuous variable, reducing reliance on specific thresholds.<br>**Risk of Bias: Low**<br>The risk of bias is low because the METS-IR calculation was objective, based on standardized laboratory and anthropometric measurements, and independent of the OSA questionnaire results. The use of tertiles as thresholds was part of a pre-specified analytical strategy, and the study's findings were consistent across continuous and categorical analyses, minimizing concerns about threshold-related bias. There is no evidence that the interpretation of METS-IR results was influenced by knowledge of the reference standard, ensuring robust methodology in this domain. | determined *post hoc* based on the distribution of the data. The authors did not pre-specify these thresholds before conducting the analysis.<br>**Risk of bias assessment: Unclear**<br>The risk of bias for the index test is unclear. While the calculations of the index tests are objective, the *post hoc* categorization of these tests into tertiles introduces a potential for bias. Choosing cut-offs after seeing the data distribution can lead to results that exaggerate the association between the index test and the outcome. | |
| | **Error: The AI could not understand that since the data was from a retrospective source when the risk indices were computed, and the idea of their study was not yet created, the assessment is blind from the point of view of the standard clinical test. Also since the indice is objective, it cannot be influenced by the standard test.** | **Error: The AI could not understand that since the data was from a retrospective source when the risk indices were computed, and the idea of their study was not yet created, the assessment is blind from the point of view of the standard clinical test.**<br>**The AI considered the use of tertiles in the protocol as a prespecification of the threshold, but tertiles are data driven, thus after the analysis. Correct prespecification involves clinical guidelines, biological reasoning, prior evidences.** | We can consider that not applicable is equivalent to Yes. | **The AI considered the use of tertiles in the protocol as a prespecification of the threshold, but tertiles are data driven, thus after the analysis. Correct prespecification involves clinical guidelines, biological reasoning, prior evidences.** |
| **Reference standard** | | | | |
| **Signaling questions:**<br><br>Is the reference standard likely to correctly classify the target condition? **No** | **Signaling Questions – Reference Standard Domain:**<br>**Is the reference standard likely to correctly classify the target condition?**<br>**Answer:** No | **Signaling Questions**<br>**Is the reference standard likely to correctly classify the target condition?**<br>**Answer**: No<br>**Were the reference standard results interpreted without knowledge of the results of the index test?**<br>**Answer**: Yes | Here are the answers to the signaling questions for the Reference Standard domain:<br>Is the reference standard likely to correctly classify the target condition? **No** | **Signaling Question 1:** Is the reference standard likely to correctly classify the target condition?<br>**Answer:** No<br>**Rationale:** OSA was defined using self-reported |

| | | | | |
|---|---|---|---|---|
| Were the reference standard results interpreted without knowledge of the results of the index test? **yes**<br>Risk of bias assessment **High**<br><br>**Rationale:**<br><br>The authors used a questionnaire, that is not accurate for the diagnosis. Instead the golden standard is the polisomnography<br>Since the indice was not computed at the time of the study it could not influence the questionnaire response.<br>Due to a poor standard the RoB is low. | **Were the reference standard results interpreted without knowledge of the results of the index test?**<br>**Answer:** Unclear<br>**Risk of Bias – Reference Standard Domain:**<br>**Answer:** High<br>**Rationale:**<br>**Validity of Reference Standard**: The study used a **self-reported sleep questionnaire** to identify participants at high risk for OSA, based on symptoms like snoring, daytime sleepiness, and breathing interruptions. This method lacks the diagnostic accuracy of polysomnography (the gold standard) and has limited sensitivity and specificity, leading to potential misclassification of OSA status. Hence, the reference standard is **not likely to correctly classify the target condition**.<br>**Blinding**: It is not stated whether the reference standard assessments (from questionnaire data) were interpreted independently of the index test data (e.g., METS-IR). As the analyses were performed using existing NHANES data and interpreted concurrently in a statistical analysis, the risk of bias due to lack of blinding cannot be ruled out—hence "unclear." | **Risk of Bias Assessment**<br>**Risk of Bias**: High<br>**Rationale**<br>**Is the reference standard likely to correctly classify the target condition? (No)**<br>The reference standard for obstructive sleep apnea (OSA) in this study was based on a sleep questionnaire from the NHANES database, where participants were considered at high risk for OSA if they met more than one of the following criteria: (1) self-reported snoring more than 3 nights per week, (2) feeling excessively sleepy 16–30 times per month despite ≥7 hours of nighttime sleep, and (3) self-reported episodes of apnea, wheezing, or snorting more than 3 nights per week. While this method has been used in previous NHANES studies and is practical for large-scale surveys, it is not the gold standard for OSA diagnosis, which is polysomnography (measuring the apnea-hypopnea index, AHI). The questionnaire-based approach relies on self-reported symptoms, which are subjective and prone to recall bias, misreporting, or misinterpretation. Additionally, the criteria identify individuals at high risk for OSA rather than providing a definitive diagnosis, leading to potential misclassification (e.g., false positives from individuals with other sleep disorders or false negatives from those with mild OSA who do not report symptoms). The study acknowledges this limitation, noting that participants meeting the criteria belong to a high-risk group but cannot be definitively diagnosed with OSA. This reliance on a non-gold-standard method increases the risk of bias in correctly classifying the target condition.<br>**Were the reference standard results interpreted without knowledge of the results of the index test? (Yes)**<br>The reference standard (OSA status) was determined based on responses to the NHANES sleep questionnaire, which was administered as part of the survey protocol. The index test, METS-IR, was calculated using objective laboratory and anthropometric data (FBG, TG, HDL-C, BMI) collected separately. The questionnaire responses were self-reported by participants and processed independently of the METS-IR calculations. There is | Were the reference standard results interpreted without knowledge of the results of the index test? **Yes**<br>Risk of bias assessment: **High**<br><br>**Is the reference standard likely to correctly classify the target condition? No**<br>In this study, the diagnosis of OSA was based on a combination of self-reported symptoms: snoring, excessive daytime sleepiness and self-reported episodes of apnea, wheezing, or snorting. Self-reported OSA symptoms are not the gold standard for diagnosing OSA. The gold standard is polysomnography, which involves overnight monitoring in a sleep laboratory. Self-reporting can be subjective and inaccurate, and is prone to both false positives and false negatives.<br>**Were the reference standard results interpreted without knowledge of the results of the index test? Yes**<br>The authors did not explicitly state that the reference standard was interpreted without knowledge of the index test. However, since the diagnosis of OSA was based on self-report, and the index tests (METS-IR, etc.) are calculated from blood tests and other measurements, it's reasonable to assume that the self-reported symptoms were collected independently of the results of the index tests.<br>**Risk of bias assessment: High**<br>There is a high risk of bias. The reference standard (self-reported symptoms) is not likely to correctly classify OSA, which can lead to misclassification bias. | symptoms (e.g., snoring, daytime sleepiness) rather than polysomnography (the gold standard), which may lead to misclassification.<br>**Signaling Question 2:** Were the reference standard results interpreted without knowledge of the index test?<br>**Answer:** Unclear<br>**Rationale:** The study does not explicitly state whether OSA assessment was blinded to the IR indices.<br>**Risk of Bias:** High<br>**Rationale:** The use of self-reported symptoms for OSA diagnosis introduces significant bias due to potential misclassification. |

| | | | | |
|---|---|---|---|---|
| | | no indication that the interpretation of the questionnaire results (i.e., determining OSA status) was influenced by knowledge of the METS-IR values, as these are distinct data collection streams within NHANES. The objective nature of METS-IR calculations and the standardized administration of the questionnaire support the conclusion that the reference standard was interpreted without knowledge of the index test results.<br>**Risk of Bias: High**<br>The high risk of bias in this domain is driven by the reference standard's inability to reliably classify OSA. The use of a questionnaire-based method, while practical for a large population study, does not match the diagnostic accuracy of polysomnography, leading to potential misclassification of OSA status. This limitation could affect the validity of the observed association between METS-IR and OSA. However, the interpretation of the reference standard was independent of the index test results, mitigating some concerns about bias in this aspect. The primary issue remains the questionnaire's limited accuracy as a proxy for OSA diagnosis, justifying the high risk of bias assessment. | | |
| | **Error: The AI could not understand that since the data was from a retrospective source when the risk indices was not computed, and the idea of their study was not yet computed, the assessment is blind from the point of view of the standard clinical test.** | | | **Error: The AI could not understand that since the data was from a retrospective source when the risk indices was not computed, and the idea of their study was not yet computed, the assessment is blind from the point of view of the standard clinical test.** |
| Flow and timing | | | | |
| **Signaling questions:**<br><br>Was there an appropriate interval between index test(s) and reference standard? **yes** | **Signaling Questions – Flow and Timing Domain:**<br>**Was there an appropriate interval between index test(s) and reference standard?**<br>**Answer:** Yes | Domain 4: Flow and Timing<br><br>*Signaling Questions*<br><br>**Was there an appropriate interval between index test(s) and reference standard?**<br>**Answer**: Yes | Here are the answers to the signaling questions for the Flow and Timing domain:<br>Was there an appropriate interval between index test(s) and reference standard? **Not Applicable** | **Signaling Question 1:** Was there an appropriate interval between index test and reference standard?<br>**Answer:** Not applicable<br>**Rationale:** This is a cross-sectional study, so the timing between tests is not relevant. |

| | | | | |
|---|---|---|---|---|
| Did all patients receive a reference standard? **Yes**<br>Did patients receive the same reference standard? **Yes**<br>Were all patients included in the analysis? **yes**<br>Risk of bias assessment **Low**<br><br>**Rationale:**<br><br>The exact time between assessments is not stated. However NHANES performs their measurements in a single session or several ones, but close to one another. Since the disease evolves slowly, it won't induce bias.<br>Not all patients were included in the study, due to exclusions. But, those who remained were all assessed with the reference standard.<br>Since there was only one reference standard mentioned, we understand that only one was used.<br>Not all patients were included in the analysis, due to extensive exclusions. Since exclusions are at selection of the sample we can consider they are not necessary for analyses, and they were assessed in the first domain. Thus, the answer is Yes. | **Did all patients receive a reference standard?**<br>**Answer:** Yes<br>**Did patients receive the same reference standard?**<br>**Answer:** Yes<br>**Were all patients included in the analysis?**<br>**Answer:** No<br>**Risk of Bias – Flow and Timing Domain:**<br>**Answer:** High<br><br>**Rationale:**<br>**Timing**: Since all variables (index tests and reference standard) were derived from the same NHANES survey cycle and collected during the same assessment period, the interval between tests is appropriate.<br>**Uniformity and Completeness of Reference Standard**: All participants included in the final sample were evaluated for OSA using the same standardized questionnaire criteria, satisfying both the "same" and "all received" questions.<br>**Inclusion in Analysis**: A substantial number of eligible participants (over 14,000) were excluded due to missing values for key variables or incomplete covariate data. This selective inclusion could bias the results and limits the applicability to the broader population, resulting in a **high risk of bias** in this domain. | **Did all patients receive a reference standard?**<br>**Answer**: Yes<br>**Did patients receive the same reference standard?**<br>**Answer**: Yes<br>**Were all patients included in the analysis?**<br>**Answer**: No<br><br>*Risk of Bias Assessment*<br><br>**Risk of Bias**: High<br><br>*Rationale*<br><br>**Was there an appropriate interval between index test(s) and reference standard? (Yes)**<br>The index test (METS-IR) was calculated using laboratory and anthropometric data (fasting blood glucose, triglycerides, HDL-cholesterol, and BMI) collected during NHANES examinations. The reference standard for OSA was based on a sleep questionnaire administered as part of the same NHANES survey cycle (2005–2008 and 2015–2018). In a cross-sectional study like this, both the index test and reference standard data are typically collected during the same study visit or within a short timeframe as part of the survey protocol. There is no indication of significant delays between the collection of biochemical/anthropometric data and the questionnaire responses, and OSA is a chronic condition where short time intervals are unlikely to affect the association. Thus, the interval between the index test and reference standard is considered appropriate.<br>**Did all patients receive a reference standard? (Yes)**<br>All participants included in the final analysis (N=8,306) had data available for the reference standard, as OSA status was determined based on the sleep questionnaire. The study excluded participants missing OSA data (N=10) before the final cohort was defined, ensuring that all participants in the analysis received the reference standard. The NHANES protocol standardizes questionnaire administration, and there is no evidence that any included participants were assessed for OSA using a different method. | Did all patients receive a reference standard? **Yes**<br>Did patients receive the same reference standard? **Yes**<br>Were all patients included in the analysis? **No**<br>Risk of bias assessment: **Unclear**<br><br>**Was there an appropriate interval between index test(s) and reference standard? Not Applicable**<br>The index tests (METS-IR, TyG index, etc.) are calculated from blood samples and other measurements taken at the time of the NHANES survey. The reference standard (self-reported OSA symptoms) is also based on information collected during the same survey. Therefore, the concept of an "interval" between the index test and reference standard, which is relevant in studies where one test is performed to predict a later diagnosis, is not directly applicable here. Both were assessed concurrently within the NHANES data collection.<br>**Did all patients receive a reference standard? Yes**<br>All participants in the study had data available regarding the self-reported symptoms used to define the reference standard for OSA. The study did not exclude participants based on missing data for the OSA assessment.<br>**Did patients receive the same reference standard? Yes**<br>The study used the same set of self-reported symptoms (snoring, excessive daytime sleepiness, and self-reported episodes of apnea, | **Signaling Question 2:** Did all patients receive the same reference standard?<br>**Answer:** Yes<br>**Rationale:** All participants were assessed for OSA using the same questionnaire-based criteria.<br>**Signaling Question 3:** Were all patients included in the analysis?<br>**Answer:** No<br>**Rationale:** Participants with missing data were excluded, which could introduce bias.<br>**Risk of Bias:** Unclear<br>**Rationale:** While the same reference standard was used, exclusions due to missing data may affect the results. |

| | | | | |
|---|---|---|---|---|
| | | **Did patients receive the same reference standard? (Yes)**<br>All participants in the final cohort were assessed for OSA using the same sleep questionnaire-based criteria, which involved self-reported snoring, daytime sleepiness, and apnea episodes. The study applied consistent criteria across all participants, ensuring that the same reference standard was used for everyone in the analysis.<br>**Were all patients included in the analysis? (No)**<br>The study started with 39,722 participants from NHANES 2005–2008 and 2015–2018, but only 8,306 were included in the final analysis after applying exclusion criteria. Exclusions included participants younger than 20 years (N=17,520), pregnant women (N=521), those with inappropriate energy intake (N=248), those missing data on OSA (N=10), those missing data on key variables like TG, HDL-C, BMI, or fasting insulin (N=12,577), and those with incomplete covariate data or sample weights (N=540). The exclusion of 12,577 participants for missing data on key variables is particularly significant, as it represents a substantial portion of the initial sample. These exclusions could introduce selection bias if the excluded participants differed systematically from those included (e.g., in terms of metabolic health or OSA risk). While the study used NHANES sampling weights to account for the complex survey design, the large number of exclusions may still compromise the representativeness of the findings.<br>**Risk of Bias: High**<br>The high risk of bias in this domain is primarily due to the significant number of participants excluded from the analysis, particularly for missing data on key variables required for METS-IR calculation. This large exclusion rate raises concerns about selection bias and the generalizability of the results. Although the interval between the index test and reference standard was appropriate, all included participants received the same reference standard, and all had OSA status assessed, the substantial loss of participants due to exclusions outweighs these strengths, leading to a high risk of bias in the flow and timing domain. | wheezing, or snorting) to define OSA for all participants.<br>**Were all patients included in the analysis? No**<br>The study had several exclusion criteria, which led to a significant reduction in the sample size. The final analysis included only 8,306 participants out of an initial 39,722. The flow chart in the Methods section of the paper shows that exclusions were made due to: age, pregnancy, abnormal energy intake, missing data on OSA and other key variables, and incomplete covariate data. These exclusions mean that not all patients were included in the final analysis.<br>**Risk of bias assessment: Unclear**<br>The risk of bias is unclear. While all patients received the same reference standard, a large number of participants were excluded, which could introduce bias if the excluded participants differed systematically from those included. | |

| | | | | |
|---|---|---|---|---|
| | **Error: The AI treated exclusions during selection (before enrolment), as missing of participants at analysis time (after enrolment), which are different issues.** | **Error: The AI treated exclusions during selection (before enrolment), as missing of participants at analysis time (after enrolment), which are different issues.** | **Error: The AI considered that if a study is cross-sectional the time interval between two assessments is irrelevant. In reality this time can be important.<br>The AI treated exclusions during selection (before enrolment), as missing of participants at analysis time (after enrolment), which are different issues.** | **Error: The AI considered that if a study is cross-sectional the time interval between two assessments is irrelevant. In reality this time can be important.<br><br>The AI treated exclusions during selection (before enrolment), as missing of participants at analysis time (after enrolment), which are different issues.** |

| Fariba, 2025 | | | | |
|---|---|---|---|---|
| **Human** | **ChatGPT 4o** | **x. AI Grok 3** | **Gemini 2.0 flash** | **DeepSeek V3** |
| **Patient selection** | | | | |
| **Signaling questions:**<br>Was a consecutive or random sample of patients enrolled? **Yes**<br>Was a case-control design avoided? **Yes**<br>Did the study avoid inappropriate exclusions? **Yes**<br>**Risk of bias: Low**<br>**Rationale:**<br><br>The authors mentioned consecutive patients<br>The authors mentioned cross-sectional and no other text would suggest otherwise<br>The exclusions concerning patients selection are justified (unstable angina, acute coronary syndrome).<br>There are some exclusions that can be made only after using the index tests, thus are not about patient selection but about flow and timing, and these exclusions are incorrect (coronary calcification and motion artifacts) since they can induce spectrum bias or diagnostic purity bias.<br>Thus the RoB is low | **Answers to Signaling Questions (Domain 1: Patient Selection):**<br>Was a consecutive or random sample of patients enrolled? **Yes**<br>Was a case-control design avoided? **Yes**<br>Did the study avoid inappropriate exclusions? **Yes**<br>**Risk of Bias Judgment (Patient Selection): Low**<br>**Rationale:**<br>The study used a **cross-sectional design** and included a **consecutive sample of 54 patients** with known or suspected CAD, as stated in the "Methods" section. This avoids selection bias.<br>There is no indication of a **case-control design** being used, which minimizes the risk of artificial differences between groups.<br>Exclusions were appropriate and clearly defined (e.g., unstable angina, ACS, significant coronary calcification, etc.), and no evidence of **selective or inappropriate exclusions** was found. | **Domain 1: Patient Selection**<br>**Was a consecutive or random sample of patients enrolled?**<br>**Answer:** Yes<br>**Rationale:** The article states that the study included "54 consecutive patients with known or suspected CAD" (Page 2). The use of the term "consecutive" indicates that patients were enrolled sequentially as they presented, which aligns with a consecutive sampling approach.<br>**Was a case-control design avoided?**<br>**Answer:** Yes<br>**Rationale:** The study is described as a cross-sectional study (Page 1 and Page 2), not a case-control design. All patients underwent both MPI and CCTA, and a subset underwent ICA, with no explicit separation into cases (e.g., CAD-positive) and controls (e.g., CAD-negative) for the purpose of the study design.<br>**Did the study avoid inappropriate exclusions?**<br>**Answer:** No<br>**Rationale:** The study excluded patients with conditions such as bypass grafts, unstable angina, acute coronary syndrome, frequent premature heartbeats, atrial fibrillation, X syndrome, significant coronary calcification, and motion artifacts impacting stenosis measurement (Page 2). While some exclusions (e.g., unstable angina, acute coronary syndrome) are justifiable to ensure patient safety or test feasibility, others, such as significant coronary calcification and motion artifacts, could introduce bias. These conditions are common in CAD populations and may affect the generalizability of the results, as they could exclude patients with more severe or complex disease, potentially skewing the | Okay, I will answer the signaling questions for the Patient Selection domain.<br>Was a consecutive or random sample of patients enrolled? Yes<br>Was a case-control design avoided? Yes<br>Did the study avoid inappropriate exclusions? No<br>Risk of bias assessment: Unclear<br>**Rationale**<br>**Was a consecutive or random sample of patients enrolled?**<br>The study mentions, "This cross-sectional study included a total of 54 consecutive patients with known or suspected CAD." This indicates a consecutive enrollment of patients.<br>**Was a case-control design avoided?**<br>The study design is described as a cross-sectional study comparing two diagnostic tests (CCTA and MPI) in patients with suspected CAD, using ICA as the reference standard. This design is not a case-control study.<br>**Did the study avoid inappropriate exclusions?**<br>The study excluded patients with several conditions: "patients having bypass grafts, unstable angina, acute coronary syndrome (ACS), often premature heartbeats, atrial fibrillation, X syndrome, significant coronary calcification, and motion artifacts impacting the measurement of the stenoses." Some of these exclusions might be appropriate, but the term "often premature heartbeats" is vague and could lead to inappropriate exclusions. | *Domain 1: Patient Selection*<br><br>**Signaling Questions:**<br>**Was a consecutive or random sample of patients enrolled?**<br>**Answer:** Unclear<br>**Rationale:** The study mentions "54 consecutive patients," but it is unclear if enrollment was truly consecutive or if selection bias existed (e.g., exclusions like bypass grafts, unstable angina).<br>**Was a case-control design avoided?**<br>**Answer:** Yes<br>**Rationale:** The study is cross-sectional, comparing MPI and CCTA in the same cohort, not case-control.<br>**Did the study avoid inappropriate exclusions?**<br>**Answer:** No<br>**Rationale:** Exclusions (e.g., bypass grafts, atrial fibrillation) might bias the sample by omitting high-risk patients.<br>**Risk of Bias: High**<br>**Rationale:** Potential selection bias due to non-consecutive enrollment and inappropriate exclusions. |

| | | | | |
|---|---|---|---|---|
| | | diagnostic performance of MPI and CCTA.<br>**Risk of Bias Assessment: Patient Selection**<br>**Answer:** High<br>**Rationale:** Although the study used a consecutive sample and avoided a case-control design, the exclusion criteria included conditions like significant coronary calcification and motion artifacts, which are prevalent in CAD patients and could bias the results by selecting a less severe or less representative patient population. This inappropriate exclusion increases the risk of bias in patient selection. | **Risk of bias assessment**<br>The risk of bias is "unclear" because while the study used a consecutive sample and avoided a case-control design, the exclusion criteria raise concerns about potential selection bias. The rationale behind excluding patients with "often premature heartbeats" is not clearly explained, and this lack of clarity introduces uncertainty about whether the exclusions were appropriate. | |
| | | **Error: The AI incorrectly identified problematic exclusions for the Patient selection domain, when in reality, the exclusions can be made only after using the test, thus these exclusions relate to the flow and timing biases.** | Error: The AI incorrectly considered often premature heartbeats as a problematic exclusion due to not being clearly explained, although it is a known situation. | **Error: The AI incorrectly interpreted the use of exclusions as an invalidation of consecutive sampling. Applying inclusion and exclusion criteria for selecting patients does not destroy a consecutive sampling.** |
| **Index test** | | | | |
| **Signaling questions:**<br><br>Were the index test results interpreted without knowledge of the results of the reference standard? **Yes**<br>If a threshold was used, was it pre-specified? **Yes**<br>Risk of bias assessment **Low**<br><br>**Rationale:**<br><br>Although the authors did not mention if the index tests were performed blindly against the standard test, they did mention the standard | **Answers to Signaling Questions (Domain 2: Index Test):**<br>Were the index test results interpreted without knowledge of the results of the reference standard? **Unclear**<br>If a threshold was used, was it pre-specified? **Yes**<br>**Risk of Bias Judgment (Index Test): Unclear**<br>**Rationale:**<br>The article does **not state explicitly** whether the interpreters of the index tests (MPI and CCTA) were **blinded to the reference standard results (ICA)**. Lack of blinding could introduce interpretation bias. | **Domain 2: Index Test**<br>**Were the index test results interpreted without knowledge of the results of the reference standard?**<br>**Answer:** Unclear<br>**Rationale:** The article does not explicitly state whether the interpreters of the index tests (MPI and CCTA) were blinded to the results of the reference standard (ICA). While it mentions that MPI was interpreted by a nuclear medicine specialist and ICA by an expert cardiologist (Page 3), there is no information provided about whether the MPI and CCTA results were evaluated without knowledge of the ICA results. The lack of clarity on blinding procedures leaves this aspect uncertain.<br>**If a threshold was used, was it pre-specified?**<br>**Answer:** Yes | Okay, I will now assess the Index Test domain.<br>Were the index test results interpreted without knowledge of the results of the reference standard? Unclear<br>If a threshold was used, was it pre-specified? Yes<br>Risk of bias assessment: Unclear<br>**Rationale**<br>**Were the index test results interpreted without knowledge of the results of the reference standard?**<br>The article does not explicitly state whether the interpreters of the index tests (CCTA and MPI) were blinded to the results of the reference standard (ICA). The methods section describes how the tests were | **Signaling Questions:**<br>**Were the index test results interpreted without knowledge of the reference standard?**<br>**Answer:** Unclear<br>**Rationale:** The article does not specify blinding of CCTA/MPI interpreters to ICA results.<br>**If a threshold was used, was it pre-specified?**<br>**Answer:** Yes (for CCTA: >70% stenosis; MPI: SSS≥1).<br>**Rationale:** Thresholds were defined in the Methods.<br>**Risk of Bias: Unclear**<br>**Rationale:** Lack of blinding details raises concerns, but pre-specified thresholds reduce bias. |

| | | | | |
|---|---|---|---|---|
| test was performed only after the index tests, thus they couldn't use it for interpretation at the time of the test<br>The authors clearly specified the thresholds for both tests.<br>Thus the RoB is low | However, the study did use **pre-specified diagnostic thresholds**: for example, **SSS ≥1 was considered abnormal for MPI**, and **≥70% luminal narrowing was considered positive for CCTA**. These thresholds are in line with clinical standards and were clearly reported. | **Rationale:** The article specifies thresholds for both index tests. For CCTA, segments with more than 70% luminal narrowing were considered indicative of severe stenosis (Page 2). For MPI, an SSS ≥ 1 was considered abnormal and low risk (positive result) (Page 3). Additionally, the study used ROC curve analysis to determine an optimal cut-off point of SSS ≥ 8.5 (Page 5), but the initial thresholds for both tests were pre-specified, as they are described in the methods section before the results.<br>**Risk of Bias Assessment: Index Test**<br>**Answer:** Unclear<br>**Rationale:** The pre-specification of thresholds for both MPI and CCTA supports a lower risk of bias in terms of test interpretation consistency. However, the lack of information regarding whether the index test results were interpreted without knowledge of the reference standard (ICA) introduces uncertainty. Without explicit mention of blinding, it cannot be confirmed that the interpretation was free from bias, leading to an unclear risk of bias for this domain. | performed and interpreted, but it doesn't mention blinding.<br>**If a threshold was used, was it pre-specified?**<br>For CCTA, segments with more than 70% luminal narrowing were considered as severe stenosis, indicating a positive result. In segments with severe calcification, it was regarded as obstructive coronary artery disease.<br>For MPI, the available reports of ischemia were determined according to vascular territories, and SSS≥1 was considered abnormal and low risk (positive result).<br>These thresholds appear to be defined in the methods, thus pre-specified.<br>**Risk of bias assessment**<br>The risk of bias for the index test is "unclear." The lack of explicit mention of blinding in the interpretation of CCTA and MPI results introduces uncertainty. While the thresholds for positive results were pre-specified, the potential for bias in interpreting the index test results without knowledge of the reference standard cannot be ruled out. | |
| | **Error: The AI didn't kept into account the fact the standard test was carried out only after the index test, and at the time they used the index test they couldn't have seen the result of the standard test. Furthermore, the standard was chosen based on the result of the index test.** | **Error: The AI didn't kept into account the fact the standard test was carried out only after the index test, and at the time they used the index test they couldn't have seen the result of the standard test. Furthermore, the standard was chosen based on the result of the index test.** | **Error: The AI didn't kept into account the fact the standard test was carried out only after the index test, and at the time they used the index test they couldn't have seen the result of the standard test. Furthermore, the standard was chosen based on the result of the index test.** | **Error: The AI didn't kept into account the fact the standard test was carried out only after the index test, and at the time they used the index test they couldn't have seen the result of the standard test. Furthermore, the standard was chosen based on the result of the index test.** |

| Reference standard | | | | |
|---|---|---|---|---|
| **Signaling questions:**<br><br>Is the reference standard likely to correctly classify the target condition? **Yes**<br>Were the reference standard results interpreted without knowledge of the results of the index test? **Unclear**<br>Risk of bias assessment **Unclear**<br><br>**Rationale:**<br><br>The authors used invasive coronary angiography, the golden standard for diagnostic of coronary artery disease<br>The blinding is not clearly stated<br>Therefore the RoB remains unclear | **Answers to Signaling Questions (Domain 3: Reference Standard):**<br>Is the reference standard likely to correctly classify the target condition? **Yes**<br>Were the reference standard results interpreted without knowledge of the results of the index test? **Unclear**<br>**Risk of Bias Judgment (Reference Standard): Unclear**<br>**Rationale:**<br>The **reference standard used was invasive coronary angiography (ICA)**, which is widely accepted as the **gold standard** for diagnosing coronary artery disease. Thus, it is **very likely to correctly classify the target condition**. However, the article does **not clarify** whether ICA interpretations were done **blinded to MPI and CCTA results**, which introduces uncertainty regarding potential review bias. | **Domain 3: Reference Standard**<br>**Is the reference standard likely to correctly classify the target condition?**<br>**Answer:** Yes<br>**Rationale:** The reference standard used in the study is invasive coronary angiography (ICA), which is widely accepted as the gold standard for diagnosing coronary artery disease (CAD). The article specifies that ICA was performed according to established clinical standards, with a stenosis of ≥50% in the left main artery or ≥70% in other arteries considered diagnostic for CAD (Page 3). These thresholds align with clinical practice, indicating that ICA is likely to correctly classify the presence or absence of CAD.<br>**Were the reference standard results interpreted without knowledge of the results of the index test?**<br>**Answer:** Unclear<br>**Rationale:** The article does not provide explicit information on whether the interpreters of the ICA results (expert cardiologists) were blinded to the results of the index tests (MPI and CCTA). While it is noted that ICA was conducted after MPI and CCTA in the study protocol (Page 2), there is no mention of blinding procedures to ensure that the ICA interpretation was independent of the index test results. This lack of detail leaves the potential for bias uncertain.<br>**Risk of Bias Assessment: Reference Standard**<br>**Answer:** Unclear<br>**Rationale:** The use of ICA as the reference standard is appropriate and likely to correctly classify CAD, supporting a low risk of bias in terms of the standard's accuracy. However, the absence of information on whether ICA results were interpreted without knowledge of the MPI and CCTA results | Alright, I'm ready to assess the Reference Standard domain.<br>Is the reference standard likely to correctly classify the target condition? Yes<br>Were the reference standard results interpreted without knowledge of the results of the index test? Unclear<br>Risk of bias assessment: Unclear<br>**Rationale**<br>**Is the reference standard likely to correctly classify the target condition?**<br>The study used invasive coronary angiography (ICA) as the reference standard, and the article states that "The gold standard for the detection of CAD was a stenosis of 50% or more narrowing of the luminal diameter of the left main or 70% or greater narrowing of the other arteries". ICA is widely accepted as the gold standard for diagnosing coronary artery disease.<br>**Were the reference standard results interpreted without knowledge of the results of the index test?**<br>The article does not explicitly state whether the interpretation of the ICA results was done without knowledge of the CCTA and MPI results. The methods section describes the ICA procedure and interpretation, but it doesn't mention blinding.<br>**Risk of bias assessment**<br>The risk of bias for the reference standard is "unclear". While ICA is an appropriate reference standard, the lack of information on blinding during the interpretation of ICA results introduces uncertainty. It's unclear whether the ICA results | **Signaling Questions:**<br>**Is the reference standard likely to correctly classify the target condition?**<br>**Answer:** Yes<br>**Rationale:** ICA is the gold standard for CAD diagnosis (stenosis ≥50% LM or ≥70% other arteries).<br>**Were the reference standard results interpreted without knowledge of the index test?**<br>**Answer:** Unclear<br>**Rationale:** No information on whether ICA interpreters were blinded to CCTA/MPI results.<br>**Risk of Bias: Unclear**<br>**Rationale:** ICA is appropriate, but blinding is not confirmed. |

| | | | | |
|---|---|---|---|---|
| | | introduces uncertainty. Without confirmation of blinding, the risk of bias in the interpretation of the reference standard remains unclear. | might have been influenced by knowledge of the index test results. | |
| | | | | |
| Flow and timing | | | | |
| **Signaling questions:**<br><br>Was there an appropriate interval between index test(s) and reference standard? **yes**<br>Did all patients receive a reference standard? **no**<br>Did patients receive the same reference standard? **Yes**<br>Were all patients included in the analysis? **No**<br>Risk of bias assessment **high**<br><br>**Rationale:**<br><br>The authors did mention 60 as the period within the tests were applied. This time interval is reasonable in the case of chronic arterial disease that does not progress rapidly.<br>The authors specify that only 18 patients with a positive and high-risk noninvasive test results underwent the standard test.<br>They mention only one standard test<br>There are some exclusions that can be | **Answers to Signaling Questions (Domain 4: Flow and Timing):**<br>Was there an appropriate interval between index test(s) and reference standard? **Yes**<br>Did all patients receive a reference standard? **No**<br>Did patients receive the same reference standard? **Yes**<br>Were all patients included in the analysis? **Yes**<br>**Risk of Bias Judgment (Flow and Timing): High**<br>**Rationale:**<br>All patients underwent CTCA and MPI within **60 days**, which is an **acceptable time interval**. However, **only 18 out of 54 patients** underwent the reference standard test (ICA), meaning that **not all patients received the reference standard**, which could bias the diagnostic accuracy results.<br>Those who did undergo ICA received the **same reference standard**, and there is no indication of patients being excluded from analysis once included.<br>Because the **partial** | **Domain 4: Flow and Timing**<br>**Was there an appropriate interval between index test(s) and reference standard?**<br>**Answer:** Yes<br>**Rationale:** The article states that CTCA, MPI, and ICA were performed within 60 days for all cases (Page 2). This interval is generally considered appropriate in diagnostic studies for CAD, as it is unlikely that significant disease progression or regression would occur within this timeframe, ensuring that the tests reflect the same underlying condition.<br>**Did all patients receive a reference standard?**<br>**Answer:** No<br>**Rationale:** Not all patients in the study received the reference standard (ICA). The article specifies that only 18 out of the 51 patients who underwent both MPI and CCTA also underwent ICA, as ICA was performed only for those with positive and high-risk noninvasive test results (Page 1). This selective application of the reference standard introduces a potential for verification bias.<br>**Did patients receive the same reference standard?**<br>**Answer:** Yes<br>**Rationale:** For the subset of patients who underwent ICA (18 patients), the reference standard was consistently applied as conventional coronary angiography, performed according to established clinical standards and interpreted by an expert cardiologist (Page 3). There is no indication that different reference | Okay, I will proceed with the Flow and Timing domain.<br>Was there an appropriate interval between index test(s) and reference standard? Yes<br>Did all patients receive a reference standard? No<br>Did patients receive the same reference standard? Yes<br>Were all patients included in the analysis? No<br>Risk of bias assessment: High<br>**Rationale**<br>**Was there an appropriate interval between index test(s) and reference standard?**<br>The study states, "Within 60 days, CTCA, MPL, and CAG were performed on every case". This suggests a reasonably close interval between the index tests and the reference standard.<br>**Did all patients receive a reference standard?**<br>No. The study clearly states, "One-third of the patients underwent ICA in the present study". This means that only a subset of patients received the reference standard (ICA), while the rest relied on the index tests.<br>**Did patients receive the same reference standard?**<br>Yes. The patients who did receive a reference standard all received ICA.<br>**Were all patients included in the analysis?** | **Signaling Questions:**<br>**Was there an appropriate interval between index tests and reference standard?**<br>**Answer:** Yes<br>**Rationale:** All tests (CCTA, MPI, ICA) were performed within 60 days.<br>**Did all patients receive the same reference standard?**<br>**Answer:** No<br>**Rationale:** Only 18/51 patients underwent ICA, introducing partial verification bias.<br>**Were all patients included in the analysis?**<br>**Answer:** Yes<br>**Rationale:** Data for all enrolled patients were analyzed.<br>**Risk of Bias: High**<br>**Rationale:** Partial verification bias (only high-risk patients underwent ICA). |

| | | | | |
|---|---|---|---|---|
| made only after using the index tests, thus are not about patient selection but about flow and timing, and these exclusions are incorrect (coronary calcification and motion artifacts) since they can induce spectrum bias or diagnostic purity bias. Due to the exclusions, the use of ICA only on positive index test subjects rise the RoB to high | **verification** (only a subset undergoing ICA) introduces potential bias, especially if that subset differs systematically, the risk of bias is judged **high**. | standards or protocols were used among these patients.<br>**Were all patients included in the analysis?**<br>**Answer:** No<br>**Rationale:** The article notes that the study initially included 54 patients, but only 51 were included in the final analysis (Page 1 and Page 3). Additionally, the comparison with the reference standard (ICA) was limited to the 18 patients who underwent ICA (Page 3). The exclusion of 3 patients from the total cohort and the limited application of ICA to a subset of patients mean that not all enrolled patients were included in the complete analysis, particularly for the ICA comparison.<br>**Risk of Bias Assessment: Flow and Timing**<br>**Answer:** High<br>**Rationale:** While the interval between the index tests and the reference standard was appropriate, the fact that only a subset of patients (18 out of 51) received the reference standard (ICA) introduces a high risk of verification bias. This selective application of ICA, based on positive or high-risk noninvasive test results, could skew the diagnostic accuracy estimates. Additionally, the exclusion of 3 patients from the analysis and the limited inclusion of patients in the ICA comparison further contribute to the high risk of bias in this domain. | No. The sensitivity, specificity, and accuracy calculations were based on the 18 patients who underwent all three procedures (CCTA, MPI, and ICA), not the entire sample of 51 patients.<br>**Risk of bias assessment**<br>The risk of bias for flow and timing is "high." The fact that only one-third of the patients received the reference standard and that the analysis was limited to those patients introduces a significant potential for bias. This selective verification means that the results might not be generalizable to the entire study population and could lead to an overestimation or underestimation of the tests' accuracy. | |
| | **Error: The AI did not observe that some exclusion criteria could have been applied only after performing the index test, thus applying to the flow and timing domain. These exclusions, brought biases to the study** | **Error: The AI did not observe that some exclusion criteria could have been applied only after performing the index test, thus applying to the flow and timing domain. These exclusions, brought biases to the study**<br>The AI considered the use of the standard test only on a subset of the patients as an answer to the signaling question were all patients included in the analysis. But this is a different issue, that can happen in case | **Error: The AI did not observe that some exclusion criteria could have been applied only after performing the index test, thus applying to the flow and timing domain. These exclusions, brought biases to the study**<br>The AI considered the use of the standard test only on a subset of the patients as an answer to the signaling question were all patients included in | **Error: The AI did not observe that some exclusion criteria could have been applied only after performing the index test, thus applying to the flow and timing domain. These exclusions, brought biases to the study** |

| | | | | |
|---|---|---|---|---|
| | | some cases are not assessed, besides those that are not assessed by the standard test. | the analysis. But this is a different issue, that can happen in case some cases are not assessed, besides those that are not assessed by the standard test. | |

| **Li, 2025** | | | | |
|---|---|---|---|---|
| **Human** | **ChatGPT 4o** | **x. AI Grok 3** | **Gemini 2.0 flash** | **DeepSeek V3** |
| **Patient selection** | | | | |
| **Signaling questions:**<br>Was a consecutive or random sample of patients enrolled?<br>**Unclear**<br>Was a case-control design avoided? **Yes**<br>Did the study avoid inappropriate exclusions?<br>**Yes**<br>**Risk of bias: Low**<br>**Rationale:**<br><br>The authors does not mention consecutive nor random sampling<br>The authors state cross-sectional and give no hint they did otherwise<br>The exclusions are appropriate<br>Therefore the RoB is low | **Answers:**<br>**Was a consecutive or random sample of patients enrolled?**<br>**— Unclear**<br>**Was a case-control design avoided? — Yes**<br>**Did the study avoid inappropriate exclusions? — Yes**<br>**Risk of bias (Patient Selection domain): — Low**<br>**Rationale:**<br>**Consecutive or random sample:** The study reports a cross-sectional design with inclusion of T2DM patients admitted between October 2019 and August 2021. However, it does not explicitly state whether the sampling was consecutive or randomized, hence marked as **Unclear**.<br>**Case-control design avoided:** The study used a **cross-sectional** approach rather than a retrospective case-control design, which reduces selection bias from artificially selected cases and controls. Thus, **Yes**.<br>**Inappropriate exclusions avoided:** The exclusion criteria (e.g., type 1 diabetes, acute complications, cardiovascular disease, liver/kidney failure, malignancy, infection, severe disability, malnutrition, mental disorders) are standard and appear clinically justified, not excessive. Hence, **Yes**.<br>**Overall risk of bias:** Based on clearly defined | **Signaling Questions and Answers:**<br>**Was a consecutive or random sample of patients enrolled?**<br>**Answer:** Unclear<br>**Was a case-control design avoided?**<br>**Answer:** Yes<br>**Did the study avoid inappropriate exclusions?**<br>**Answer:** Yes<br>**Risk of Bias Assessment:**<br>**Unclear**<br>**Rationale for Answers:**<br>**Was a consecutive or random sample of patients enrolled? (Unclear)**<br>The article does not explicitly state whether the 83 T2DM subjects were enrolled consecutively or randomly. It mentions that participants were admitted to the Department of Endocrinology between October 2019 and August 2021 and met specific inclusion criteria, but there is no clear indication of the sampling method (e.g., consecutive, random, or convenience sampling). Without this information, it is unclear whether the selection process minimizes bias.<br>**Was a case-control design avoided? (Yes)**<br>The study is described as a cross-sectional study, not a case-control design. Participants were all T2DM patients, divided into two groups (DM-AS and DM+AS) based on CIMT status (a diagnostic outcome) rather than being selected as cases (with atherosclerosis) and controls (without atherosclerosis) upfront. This approach aligns with a cohort-like structure within a cross-sectional framework, avoiding a case-control design.<br>**Did the study avoid inappropriate exclusions? (Yes)**<br>The exclusion criteria are clearly outlined and include type 1 diabetes, other specific | Here are the answers to the signaling questions and the risk of bias assessment for the patient selection domain:<br>Was a consecutive or random sample of patients enrolled?<br>**No**<br>Was a case-control design avoided? **Yes**<br>Did the study avoid inappropriate exclusions? **No**<br>Risk of bias assessment: **High**<br>**Rationale:**<br>**Was a consecutive or random sample of patients enrolled? No.** The study recruited patients who were admitted to the Department of Endocrinology between October 2019 and August 2021. This is a convenience sample and not a consecutive or random sample, which increases the risk of selection bias.<br>**Was a case-control design avoided? Yes.** The study is a cross-sectional study comparing T2DM patients with and without carotid atherosclerosis, not a case-control study.<br>**Did the study avoid inappropriate exclusions? No.** The study excluded patients with pre-existing cardiovascular diseases, which is appropriate, but also excluded patients with other specific types of diabetes, liver or kidney failure, | **Domain 1: Patient Selection**<br>**Signaling Questions:**<br>Was a consecutive or random sample of patients enrolled?<br>**Answer:** unclear<br>**Risk of bias:** unclear<br>Was a case-control design avoided?<br>**Answer:** yes<br>**Risk of bias:** low<br>Did the study avoid inappropriate exclusions?<br>**Answer:** yes<br>**Risk of bias:** low<br>**Rationale:**<br>The study used a cross-sectional design, avoiding a case-control approach (low bias).<br>The method of patient enrollment (consecutive/random) was not explicitly stated (unclear bias).<br>Exclusion criteria were clearly defined and clinically justified (low bias). |

| | | | | |
|---|---|---|---|---|
| | inclusion/exclusion criteria and use of an observational cross-sectional design without retrospective selection of cases/controls, the **risk of bias is Low**, despite the unclear sampling method. | types of diabetes, recent acute complications (e.g., diabetic ketoacidosis), cardiovascular diseases, liver or kidney failure, malignant tumors, current infections, severe disability, severe malnutrition, and mental disorders. These exclusions are appropriate for a study focusing on T2DM patients without pre-existing cardiovascular diseases to ensure the study population is relevant to the research question and to minimize confounding factors that could affect CIMT or Metrnl levels.<br>**Risk of Bias Assessment: Unclear**<br>The risk of bias is rated as unclear primarily due to the lack of information on whether a consecutive or random sampling method was used. While the study avoids a case-control design and uses appropriate exclusion criteria, the uncertainty about patient enrollment introduces potential for selection bias, which cannot be fully evaluated without further details on the sampling process. | malignant tumors, current infectious conditions, severe disability, severe malnutrition, or mental disorders. These exclusions could lead to a selection bias, as the study population may not be representative of the general T2DM population.<br>**Risk of bias assessment: High.** The sampling method was not consecutive or random, and there were several exclusions that could have led to selection bias. | |
| | | | **Error: The AI considered the absence of any reported information on sampling as a proof of absence of consecutive or random sampling**<br>**The AI considered justified exclusion as inappropriate exclusions** | |
| **Index test** | | | | |
| **Signaling questions:**<br><br>Were the index test results interpreted without knowledge of the results of the reference standard? **Yes**<br>If a threshold was used, was it pre-specified? **No** | **Answers to Signaling Questions (Index Test Domain):**<br>Were the index test results interpreted without knowledge of the results of the reference standard? **Unclear**<br>If a threshold was used, was it pre-specified? **No**<br>**Risk of Bias (Index Test Domain): High** | **Rationale for Answers:**<br>**Were the index test results interpreted without knowledge of the results of the reference standard? (Unclear)**<br>The article does not provide explicit information on whether the measurement of serum Metrnl levels (the index test, measured via ELISA) was conducted without knowledge of the carotid atherosclerosis status (the reference standard, determined by CIMT via | Here are the answers to the signaling questions and the risk of bias assessment for the index test domain:<br>Were the index test results interpreted without knowledge of the results of the reference standard? **Yes**<br>If a threshold was used, was it pre-specified? **No** | **Signaling Questions:**<br>Were the index test results interpreted without knowledge of the reference standard?<br>**Answer:** unclear<br>**Risk of bias:** unclear<br>If a threshold was used, was it pre-specified?<br>**Answer:** no<br>**Risk of bias:** high<br>**Rationale:** |

| | | | | |
|---|---|---|---|---|
| Risk of bias assessment **high**<br><br>**Rationale:**<br><br>The authors did not specified the use of blinding in the study, but since the index test is a serum measurement it cannot be influenced by the standard test<br>No threshold was specified before, it was computed post-hoc<br>The absence of prespecified threshold rises the RoB to high | **Rationale:**<br>**Blinded interpretation**: There is no mention in the methods section indicating that the interpretation of serum Metrnl levels (index test) was conducted without knowledge of CIMT results (reference standard). Thus, the potential for diagnostic review bias cannot be ruled out.<br>**Threshold specification**: The threshold (0.61 ng/ml) for serum Metrnl was determined post hoc using ROC curve analysis to optimize sensitivity and specificity. Since it was derived from the data, it was not pre-specified. | ultrasound). There is no mention of blinding procedures or whether the laboratory personnel analyzing Metrnl levels were unaware of the CIMT results. Without this information, it is unclear whether the interpretation of the index test was independent of the reference standard, which could introduce bias if knowledge of CIMT status influenced Metrnl measurement or reporting.<br>**If a threshold was used, was it pre-specified? (No)**<br>The study uses a threshold of 0.61 ng/ml for serum Metrnl levels to predict carotid atherosclerosis, as determined by ROC curve analysis, with a sensitivity of 77.6% and specificity of 58.8%. However, this threshold was not pre-specified; it was derived post hoc from the ROC analysis based on the Youden index (sensitivity + specificity - 1). The article does not indicate that a specific Metrnl threshold was established before data collection, which increases the risk of bias as the threshold was optimized based on the study data, potentially inflating diagnostic accuracy.<br>**Risk of Bias Assessment: High**<br>The risk of bias is rated as high due to the lack of a pre-specified threshold for the index test (Metrnl levels), which introduces a significant risk of bias in the diagnostic accuracy results. The post hoc determination of the 0.61 ng/ml cut-off via ROC analysis could lead to overfitting and overestimation of the test's performance. Additionally, the unclear information regarding blinding of index test interpretation further supports the potential for bias, though it is not the primary driver of the high-risk rating. | Risk of bias assessment: **Unclear**<br>**Rationale:**<br>**Were the index test results interpreted without knowledge of the results of the reference standard?**<br>**Yes.** The serum Metrnl level was measured by ELISA assay, and the CIMT was measured by color doppler ultrasonography. It is plausible that the interpretation of the index test (Metrnl level) was done without knowledge of the reference standard (CIMT status).<br>**If a threshold was used, was it pre-specified? No.** The threshold for CIMT status was pre-defined as CIMT <1.0mm for the DM-AS group and CIMT ≥1.0mm or carotid plaque for the DM+AS group. However, the optimal cut-off value for serum Metrnl level was determined using the ROC curve analysis, which is post hoc, not pre-specified.<br>**Risk of bias assessment: Unclear.** While it appears the index test was interpreted without knowledge of the reference standard, the post hoc determination of the Metrnl level threshold introduces potential bias. | The study did not clarify whether Metrnl levels were measured blind to CIMT results (unclear bias).<br>The ROC-derived cut-off (0.61 ng/ml) was determined post hoc, not pre-specified (high bias). |
| | **Error: The index test being a biomarker serum evaluation it cannot be influenced by the standard test.** | **Error: The index test being a biomarker serum evaluation it cannot be influenced by the standard test.** | | **Error: The index test being a biomarker serum evaluation it cannot be influenced by the standard test.** |

| Reference standard | | | | |
|---|---|---|---|---|
| **Signaling questions:**<br><br>Is the reference standard likely to correctly classify the target condition? **No**<br>Were the reference standard results interpreted without knowledge of the results of the index test?<br>**Unclear**<br>Risk of bias assessment<br>**High**<br><br>**Rationale:**<br><br>The authors used echography as the golden standard for diagnostic of carotid atherosclerosis, but it is not as accurate as the Catheter-based digital subtraction angiography<br>The blinding was not stated, but it is unlikely that an poorly known biomarker might have influenced the standard test assesment<br>Therefore the RoB remains high due to the improper standard test | **Answers to Signaling Questions (Reference Standard Domain):**<br>Is the reference standard likely to correctly classify the target condition? — **Yes**<br>Were the reference standard results interpreted without knowledge of the results of the index test? — **Unclear**<br>**Risk of Bias (Reference Standard Domain):** — **Unclear**<br>**Rationale:**<br>**Correct classification:** Carotid ultrasound with CIMT and plaque detection is a widely accepted and validated method for diagnosing subclinical atherosclerosis, especially in diabetes research. Hence, it is appropriate and likely to correctly classify the target condition. **Yes**.<br>**Blinded interpretation:** The article does not state whether sonographers or outcome assessors were blinded to the Metrnl levels when performing or interpreting the CIMT measurements. Therefore, the risk of bias from unblinded interpretation remains **Unclear**. | **Signaling Questions and Answers:**<br>**Is the reference standard likely to correctly classify the target condition?**<br>**Answer:** Yes<br>**Were the reference standard results interpreted without knowledge of the results of the index test?**<br>**Answer:** Unclear<br>**Risk of Bias Assessment:**<br>**Unclear**<br><br>**Rationale for Answers:**<br>**Is the reference standard likely to correctly classify the target condition? (Yes)**<br>The reference standard in this study is carotid intima-media thickness (CIMT) measured by color Doppler ultrasonography, used to classify carotid atherosclerosis. CIMT is a well-established and widely accepted method for assessing subclinical atherosclerosis, particularly in the context of cardiovascular risk assessment. The study defines carotid atherosclerosis as CIMT $\geq$ 1.0 mm or the presence of carotid plaque (focal wall thickening >50% of surrounding IMT or CIMT $\geq$ 1.5 mm), which aligns with guidelines from the American Society of Echocardiography and other consensus statements. The examinations were performed by trained and certified sonographers following a standardized protocol, which supports the reliability of the reference standard. Therefore, CIMT is likely to correctly classify the target condition (carotid atherosclerosis) in this study.<br>**Were the reference standard results interpreted without knowledge of the results of the index test? (Unclear)**<br>The article does not provide information on whether the sonographers or personnel interpreting the CIMT ultrasound results | Here are the answers to the signaling questions and the risk of bias assessment for the reference standard domain:<br>Is the reference standard likely to correctly classify the target condition? **Yes**<br>Were the reference standard results interpreted without knowledge of the results of the index test? **Yes**<br>Risk of bias assessment: **Low**<br>**Rationale:**<br>**Is the reference standard likely to correctly classify the target condition? Yes.** Carotid intima-media thickness (CIMT) measured by carotid artery ultrasound is a well-established and reliable method for assessing carotid atherosclerosis, as stated in the article: "Carotid artery ultrasound was a long-standing and reliable tool in the current armamentarium of diagnostic modalities used to assess vascular morbidity at an early stage". The guidelines used for the measurement are also from the American Society of Echocardiography, further supporting its validity.<br>**Were the reference standard results interpreted without knowledge of the results of the index test? Yes.** As mentioned in the index test rationale, the CIMT was measured using ultrasound, while the index test (Metrnl level) was measured using an ELISA | **Signaling Questions:**<br>Is the reference standard likely to correctly classify the target condition?<br>**Answer:** yes<br>**Risk of bias:** low<br>Were the reference standard results interpreted without knowledge of the index test?<br>**Answer:** unclear<br>**Risk of bias:** unclear<br>**Rationale:**<br>CIMT is a validated measure for atherosclerosis (low bias).<br>Blinding of sonographers to Metrnl levels was not reported (unclear bias). |

| | | | | |
|---|---|---|---|---|
| | | were blinded to the serum Metrnl levels (the index test). There is no mention of blinding procedures or whether the ultrasound assessments were conducted independently of the Metrnl measurements. Without explicit details on the independence of the reference standard interpretation, it is unclear whether knowledge of Metrnl levels could have influenced the CIMT results, introducing potential bias.<br>**Risk of Bias Assessment: Unclear**<br>The risk of bias is rated as unclear because, while the reference standard (CIMT) is reliable and likely to correctly classify carotid atherosclerosis, the lack of information on whether CIMT results were interpreted without knowledge of Metrnl levels introduces uncertainty. If the reference standard interpretation was not blinded, there could be a risk of bias, but the absence of explicit details prevents a definitive high or low risk assessment. | assay. It is reasonable to assume that these two measurements were conducted and interpreted independently.<br>**Risk of bias assessment: Low.** The reference standard used is appropriate and likely to correctly classify the condition, and it was interpreted independently of the index test. | |
| | **Error: The AI considered that if a method is frequently used it might be also a good gold standard, which is not the case.** | **Error: The AI considered that if a method is frequently used it might be also a good gold standard, which is not the case.** | **Error: The AI considered if the authors state "well-established and reliable method", and the use of guidelines of American Society of Echocardiography, as an argument for gold standard, but it is an argument of frequent use.**<br>**The AI asserted that it is reasonable to assume independence based on the fact the two methods are of different types, a reasoning flow.** | There is no argument |
| Flow and timing | | | | |
| **Signaling questions:**<br><br>Was there an appropriate interval between index test(s) and reference standard? **Yes** | **Answers to Signaling Questions (Flow and Timing Domain):**<br>Was there an appropriate interval between index test(s) and reference standard? — **Yes** | **Signaling Questions and Answers:**<br><br>**Was there an appropriate interval between index test(s) and reference standard?**<br>**Answer:** Unclear | Here are the answers to the signaling questions and the risk of bias assessment for the flow and timing domain:<br>Was there an appropriate interval between index test(s) | **Signaling Questions:**<br>Was there an appropriate interval between index test and reference standard?<br>**Answer:** yes<br>**Risk of bias:** low |

| | | | | |
|---|---|---|---|---|
| Did all patients receive a reference standard? **Yes**<br>Did patients receive the same reference standard? **Yes**<br>Were all patients included in the analysis? **No**<br>Risk of bias assessment **Low**<br><br>**Rationale:**<br><br>The authors did not mention the time between assessments, but it should have been within the same visit, a blood test and within a few days the echography exam. Due to the nature of the disease that evolves slowly the time is appropriate<br>There is no mention or data suggesting that not all patients received the standard test<br>They mention only one standard test<br>There is no mention or data suggesting that not all patients were analysed.<br>Thus, the RoB is low | Did all patients receive a reference standard? — **Yes**<br>Did patients receive the same reference standard? — **Yes**<br>Were all patients included in the analysis? — **Yes**<br>**Risk of Bias (Flow and Timing Domain):** — **Low**<br>**Rationale:**<br>**Interval:** Both the serum Metrnl measurement (index test) and carotid ultrasound (reference standard) were part of the same clinical visit/evaluation, as implied by the cross-sectional design. Therefore, the timing is appropriate.<br>**Complete reference testing:** All 83 participants underwent CIMT assessment.<br>**Same standard used:** CIMT via color Doppler ultrasound was used uniformly as the reference standard.<br>**Complete data inclusion:** The analysis includes all 83 participants, and no dropouts or exclusions from the final analysis are reported.<br>This domain shows a **Low** risk of bias. | **Did all patients receive a reference standard?**<br>**Answer:** Yes<br>**Did patients receive the same reference standard?**<br>**Answer:** Yes<br>**Were all patients included in the analysis?**<br>**Answer:** Yes<br><br>**Risk of Bias Assessment: Unclear**<br><br>Rationale for Answers:<br><br>**Was there an appropriate interval between index test(s) and reference standard? (Unclear)**<br>The article does not provide specific information about the timing between the index test (serum Metrnl level measurement via ELISA) and the reference standard (CIMT measurement via ultrasound). While it is implied that both tests were conducted as part of the cross-sectional study protocol, there is no explicit mention of the interval between blood sample collection for Metrnl measurement and the ultrasound examination. For a condition like carotid atherosclerosis, which is relatively stable over short periods, a short interval (e.g., same day or within a few days) would be appropriate. However, without details on the timing, it is unclear whether the interval was appropriate or if any delays could have affected the results.<br>**Did all patients receive a reference standard? (Yes)**<br>The article states that all 83 T2DM subjects underwent carotid ultrasound evaluation to assess CIMT status, which served as the reference standard for determining carotid atherosclerosis. There is no indication that any participants were excluded from | and reference standard? **Unclear**<br>Did all patients receive a reference standard? **Yes**<br>Did patients receive the same reference standard? **Yes**<br>Were all patients included in the analysis? **Yes**<br>Risk of bias assessment: **Unclear**<br>**Rationale:**<br>**Was there an appropriate interval between index test(s) and reference standard? Unclear.** The article mentions that "fasting venous blood samples for the laboratory measurements were collected using Vacutainer Ethylenediaminetetraacetic Acid (EDTA) tubes in the morning after 10-hour overnight fasting" and that "carotid sonography was measured using the same color ultrasonography with a 7.0~10 MHz transducer." However, the exact time interval between the blood draw for Metrnl level measurement and the carotid ultrasound is not specified. It is unclear if the tests were performed on the same day or if there was a delay.<br>**Did all patients receive a reference standard? Yes.** All participants underwent carotid ultrasound to determine CIMT status, which served as the reference standard.<br>**Did patients receive the same reference standard?** | Did all patients receive the same reference standard?<br>**Answer:** yes<br>**Risk of bias:** low<br>Were all patients included in the analysis?<br>**Answer:** yes<br>**Risk of bias:** low<br>**Rationale:**<br>Metrnl and CIMT measurements were performed contemporaneously (low bias).<br>All 83 subjects were analyzed without attrition (low bias). |

| | | | | |
|---|---|---|---|---|
| | | receiving the ultrasound examination, confirming that all enrolled patients received the reference standard.<br>**Did patients receive the same reference standard? (Yes)**<br>All patients underwent the same reference standard, which was CIMT measurement using color Doppler ultrasonography with a 7.0–10 MHz transducer, performed by trained and certified sonographers following a standardized protocol. The criteria for defining carotid atherosclerosis (CIMT ≥ 1.0 mm or presence of carotid plaque) were consistently applied across all participants, ensuring that the same reference standard was used for all.<br>**Were all patients included in the analysis? (Yes)**<br>The study reports that a total of 83 T2DM subjects were evaluated, and all were included in the analysis, as evidenced by the results comparing the DM-AS (n=34) and DM+AS (n=49) groups. There is no mention of dropouts, withdrawals, or exclusions from the analysis after enrollment, indicating that all patients were included in the final analysis.<br><br>**Risk of Bias Assessment: Unclear**<br>The risk of bias is rated as unclear due to the lack of information regarding the interval between the index test (Metrnl measurement) and the reference standard (CIMT ultrasound). While the study confirms that all patients received the same reference standard and were included in the analysis, the uncertainty about the timing interval introduces a potential risk of bias, as an inappropriate interval could affect the relationship between Metrnl levels and CIMT status. Without explicit details, a definitive low or high risk cannot be determined. | **Yes.** All patients received carotid ultrasound as the reference standard.<br>**Were all patients included in the analysis? Yes.** The study included all 83 subjects with T2DM in the final analysis.<br>**Risk of bias assessment: Unclear.** The lack of clarity regarding the time interval between the index test and the reference standard makes it difficult to assess the potential for bias due to differential timing. | |
| | | **Error: the AI did not kept into account the realities of assessing a participant,** | **Error: the AI did not kept into account the realities of** | |

| | | | | |
|---|---|---|---|---|
| | | **and that the time between the two assessments is not big enough for atherosclerosis to evolve** | **assessing a participant, and that the time between the two assessments is not big enough for atherosclerosis to evolve** | |

| Willems, 2025 | | | | |
|---|---|---|---|---|
| **Human** | **ChatGPT 4o** | **x. AI Grok 3** | **Gemini 2.0 flash** | **DeepSeek V3** |
| **Patient selection** | | | | |
| **Signaling questions:**<br>Was a consecutive or random sample of patients enrolled? **Unclear**<br>Was a case-control design avoided? **Yes**<br>Did the study avoid inappropriate exclusions? **No**<br>**Risk of bias: Low**<br>**Rationale:**<br><br>The authors did not mention whether consecutive or random sampling was used.<br>No indication of case-control design<br>The exclusions are appropriate with the exception of Patients where CTA could not reliably determine presence or absence of ≥50% stenosis due to extensive crural (distal leg) calcifications, that can induce a post hoc exclusion (for flow and timing), with a spectrum bias (unclear cases removed). Being for flow and timing domain, it is not problematic for patient selection<br>Thus the RoB is unclear | **Answers to Signaling Questions (Patient Selection):**<br>Was a consecutive or random sample of patients enrolled? **Yes**<br>Was a case-control design avoided? **Yes**<br>Did the study avoid inappropriate exclusions? **Yes**<br>**Risk of bias (Patient Selection domain): Low**<br>**Rationale:**<br>The study included a **consecutive series** of patients with suspected PAD undergoing both duplex ultrasound and CTA, minimizing selection bias.<br>It was **not a case-control study**, but rather a retrospective diagnostic cohort, which aligns with the intended use of the QUADAS-2 tool.<br>Exclusions were appropriate and clearly stated: patients were excluded if the time between tests was too long, if interventions occurred between tests, or if calcifications prevented reliable CTA interpretation. These criteria are justifiable to maintain the accuracy of the reference standard, and do not introduce substantial bias. | **Signaling Questions:**<br>**Was a consecutive or random sample of patients enrolled?**<br>**Answer:** Unclear<br>**Was a case-control design avoided?**<br>**Answer:** Yes<br>**Did the study avoid inappropriate exclusions?**<br>**Answer:** Yes<br>**Risk of Bias Assessment: Unclear**<br>**Rationale:**<br>**Was a consecutive or random sample of patients enrolled?**<br>The article does not explicitly state whether a consecutive or random sample of patients was enrolled. It mentions that patients aged 18 years and older with suspected peripheral arterial disease (PAD) who underwent point-of-care duplex ultrasound (DUS) measurements and computed tomography angiography (CTA) were eligible for inclusion. However, there is no clear description of whether the enrollment was consecutive (e.g., all eligible patients in a given time frame) or random (e.g., a random subset of eligible patients). The retrospective nature of the study and the lack of specific details about the sampling method lead to an "Unclear" rating.<br>**Was a case-control design avoided?**<br>The study is described as a retrospective diagnostic accuracy study, not a case-control study. It includes patients suspected of PAD who underwent both the index tests (ACC_max and AT) and the reference standard (CTA), with outcomes determined based on CTA findings (stenosis >50% defining PAD). There is no indication of selecting separate groups of confirmed PAD cases and non-PAD controls, which would characterize a case-control design. Thus, the | Here's the rationale for the patient selection domain:<br>**Was a consecutive or random sample of patients enrolled? No.** The study design was retrospective, and patients were included based on having suspected PAD and undergoing point-of-care DUS measurements before CTA. This selection process, based on suspicion of PAD, does not represent a consecutive or random sample of all patients.<br>**Was a case-control design avoided? Yes.** The study included patients suspected of having the condition of interest (PAD) and evaluated the diagnostic test's accuracy in this group, using CTA as the reference standard. It did not compare patients with PAD to a separate group of patients without PAD as a control group, which would be characteristic of a case-control study.<br>**Did the study avoid inappropriate exclusions? No.** Patients were excluded if there was a time gap exceeding 90 days between the point-of-care DUS and CTA, or if CTA could not reliably differentiate between the presence or absence of a 50% stenosis due to extensive crural calcifications. These exclusions, while potentially | |

| | | | | |
|---|---|---|---|---|
| | | answer is "Yes," as a case-control design was avoided.<br>**Did the study avoid inappropriate exclusions?**<br>The study outlines specific exclusion criteria: a time gap exceeding 90 days between point-of-care DUS and CTA, any vascular intervention between these tests, and cases where CTA could not reliably differentiate the presence or absence of a 50% stenosis due to extensive crural calcifications (unless a >50% stenosis was already detected proximally). These criteria are appropriate to ensure the reliability of the diagnostic comparison and to align the timing of the index and reference tests, minimizing bias from disease progression or interventions. Patients were not excluded based on characteristics that would inappropriately skew the sample (e.g., excluding all patients with diabetes or chronic kidney disease). Therefore, the answer is "Yes," as inappropriate exclusions were avoided.<br>**Risk of Bias Assessment: Unclear**<br>The overall risk of bias for patient selection is rated as "Unclear" primarily due to the lack of clarity regarding whether a consecutive or random sample was enrolled. While the study avoided a case-control design and inappropriate exclusions, the uncertainty about the sampling method introduces potential selection bias that cannot be fully evaluated based on the provided information. The high prevalence of PAD (87.6%) in the cohort and the retrospective design further suggest possible spectrum bias, but this is not sufficient to rate the risk as "High" without additional evidence of systematic bias in patient selection. | justifiable, could introduce bias by excluding certain patient subgroups.<br>**Risk of bias assessment: Unclear.** Due to the non-consecutive patient selection and the exclusions, there's a potential for selection bias. It is unclear if the included patients are representative of the population with suspected PAD. | |

| | | | | |
|---|---|---|---|---|
| | **Error: The AI mentioned consecutive, although the authors did not mention the word.** | | **Error: Although the authors are not mentioning anything about selection, the AI answered the question with No.**<br>**The AI selected as response inappropriate exclusions but gave no medical rationale for them, saying only that they might induce bias** | **Error: Although the authors are not mentioning anything about selection, the AI answered the question with No.**<br>**The AI selected as response inappropriate exclusions but gave no medical rationale for them, saying only that they might induce bias** |
| **Index test** | | | | |
| **Signaling questions:**<br><br>Were the index test results interpreted without knowledge of the results of the reference standard? **Yes**<br>If a threshold was used, was it pre-specified? **Yes**<br>Risk of bias assessment **Low**<br><br>**Rationale:**<br><br>The authors clearly stated that the index test was before the standard test, thus ensuring the blind assessment for it<br>The authors clearly prespecified the thresholds for the methods.<br>Thus the RoB is low | **Answers to Signaling Questions (Index Test):**<br>Were the index test results interpreted without knowledge of the results of the reference standard? **Yes**<br>If a threshold was used, was it pre-specified? **Yes**<br>**Risk of bias (Index Test domain): Low**<br>**Rationale:**<br>The study explicitly states that **only patients in whom point-of-care duplex ultrasound (index test) was performed before the CTA (reference standard)** were included, ensuring **blinded interpretation**.<br>For ACCmax, the threshold values were **pre-specified** based on prior research. For AT, both **pre-specified (121 ms)** and **calculated thresholds** were used. The QUADAS-2 guideline permits analysis using both pre-specified and data-derived thresholds, provided | **Signaling Questions:**<br>**Were the index test results interpreted without knowledge of the results of the reference standard?**<br>**Answer:** Yes<br>**If a threshold was used, was it pre-specified?**<br>**Answer:** Yes<br>**Risk of Bias Assessment:**<br>**Low**<br>**Rationale:**<br>**Were the index test results interpreted without knowledge of the results of the reference standard?**<br>The article states that "to ensure assessor blindness, only those patients in whom point-of-care DUS was performed before CTA were included." This indicates that the index tests (maximal systolic acceleration [ACC_max] and acceleration time [AT]) were conducted and interpreted prior to the reference standard (CTA), ensuring that the sonographers were blinded to the CTA results. This temporal separation and explicit mention of assessor blindness support a "Yes" answer, as it minimizes the risk of bias from knowledge of the reference standard influencing the index test interpretation.<br>**If a threshold was used, was it pre-specified?** | Okay, I will now answer the signaling questions for the index test domain.<br>Were the index test results interpreted without knowledge of the results of the reference standard? **Yes**<br>If a threshold was used, was it pre-specified? **No**<br>Risk of bias assessment: **Unclear**<br>Here's the rationale for the index test domain:<br>**Were the index test results interpreted without knowledge of the results of the reference standard? Yes.** The study clearly states that only patients in whom point-of-care DUS was performed *before* CTA were included. This temporal sequence ensures that the DUS results (index test) were interpreted without knowledge of the CTA findings (reference standard), maintaining assessor blinding.<br>**If a threshold was used, was it pre-specified? No.** For Acceleration Time (AT), the | |

| | | | | |
|---|---|---|---|---|
| | this is transparent, which was the case here. | The study used thresholds for both ACC_max and AT. For ACC_max, the article specifies that "previous research identified optimal cut-off values of ACC_max for PTA and ATA at 5.55 and 5.15 m/s² to diagnose PAD, respectively," and these prespecified cut-off values were used in the diagnostic comparison analysis. For AT, the study evaluated a prespecified cut-off value of 121 ms, as mentioned by Teso et al., and also determined optimal cut-off values (112.50 ms for PTA and 119.50 ms for ATA) based on the Youden index. The use of prespecified thresholds (from prior studies for ACC_max and Teso et al. for AT) for the primary comparison, alongside the calculated thresholds for additional analysis, supports a "Yes" answer. The prespecification of thresholds reduces the risk of data-driven threshold selection that could inflate diagnostic accuracy.<br>**Risk of Bias Assessment: Low**<br>The risk of bias for the index test domain is rated as "Low" because the index test results were interpreted without knowledge of the reference standard, as ensured by the study design (DUS performed before CTA and assessor blindness). Additionally, the use of prespecified thresholds for both ACC_max and AT minimizes the risk of bias from post-hoc threshold adjustments. There are no indications of methodological flaws in the conduct or interpretation of the index tests that would suggest a higher risk of bias. | study determined optimal cut-off values using the Youden Index. While the study also included a pre-specified value of 121 ms for AT, the primary analysis used cut-off values derived from the data. The thresholds were not strictly pre-specified in the study design.<br>**Risk of bias assessment: Unclear.** Although the index test was interpreted without knowledge of the reference standard, the absence of pre-specified thresholds for AT introduces a potential source of bias. Using a data-derived threshold can lead to overfitting and an overestimation of diagnostic accuracy. | |
| | | | Obs: Since there are multiple tests we chose one of the two, thus the assessment of the AI can be considered correct | |
| **Reference standard** | | | | |
| **Signaling questions:**<br><br>Is the reference standard likely to correctly classify the target condition? **Yes** | **Answers to Signaling Questions (Reference Standard):**<br>Is the reference standard likely to correctly classify the target condition? **Yes** | **Signaling Questions:**<br>**Is the reference standard likely to correctly classify the target condition?**<br>**Answer:** Yes<br>**Were the reference standard results interpreted without knowledge of the** | Okay, I will answer the signaling questions for the reference standard domain.<br>Is the reference standard likely to correctly classify the target condition? **Yes** | **Signaling Questions:**<br>Is the reference standard likely to correctly classify the target condition?<br>**Answer:** Yes |

| | | | | |
|---|---|---|---|---|
| Were the reference standard results interpreted without knowledge of the results of the index test? **Unclear**<br>Risk of bias assessment **Unclear**<br><br>**Rationale:**<br><br>The authors used computed tomography angiography, which is close to the best golden standard for diagnostic peripheral arterial disease, especially when not used in case with a lot of calcifications, which they avoided<br>Blinding is not clearly stated<br>Therefore the RoB remains unclear | Were the reference standard results interpreted without knowledge of the results of the index test? **Yes**<br>**Risk of bias (Reference Standard domain): Low**<br>**Rationale:**<br>The reference standard used was **computed tomography angiography (CTA)**, which is a widely accepted, high-accuracy method for diagnosing peripheral arterial disease (PAD), especially when evaluating stenosis >50%.<br>The study notes that **only patients who underwent the index test before the reference test** were included, which supports the assumption of **blinded interpretation** of CTA results with respect to the index test outcomes.<br>CTA assessments were performed by **vascular radiologists**, further supporting the credibility and objectivity of the reference standard. | **results of the index test?**<br>**Answer:** Unclear<br>**Risk of Bias Assessment: Unclear**<br>**Rationale:**<br>**Is the reference standard likely to correctly classify the target condition?**<br>The reference standard used in the study is computed tomography angiography (CTA), with peripheral arterial disease (PAD) defined as a stenosis >50% from the distal abdominal aorta to the distal tibial arteries. CTA is a widely accepted imaging modality for diagnosing PAD, capable of visualizing arterial stenosis with high accuracy. The article notes that all CTAs were assessed by vascular radiologists, which supports the reliability of the interpretation. While the study acknowledges a limitation that CTA may have challenges in evaluating calcified crural arteries, it mitigated this by excluding cases where CTA could not reliably differentiate the presence or absence of a 50% stenosis (unless a >50% stenosis was already detected proximally). This approach ensures that the reference standard is likely to correctly classify PAD, supporting a "Yes" answer.<br>**Were the reference standard results interpreted without knowledge of the results of the index test?**<br>The article does not explicitly state whether the vascular radiologists interpreting the CTA results were blinded to the point-of-care duplex ultrasound (DUS) results (ACC_max and AT). While it is mentioned that DUS was performed before CTA to ensure assessor blindness for the index test, there is no clear indication of whether the CTA interpreters were unaware of the DUS findings. Given the retrospective nature of the study and the lack of specific information about blinding procedures for the reference standard interpretation, it is not possible to confirm whether the CTA results | Were the reference standard results interpreted without knowledge of the results of the index test? **Yes**<br>Risk of bias assessment: **Low**<br>Here's the rationale for the reference standard domain:<br>**Is the reference standard likely to correctly classify the target condition? Yes.** The reference standard in this study is Computed Tomography Angiography (CTA), which is a well-established and reliable method for detecting and quantifying PAD. CTA is capable of visualizing the arterial anatomy and accurately identifying the presence and degree of stenosis, which aligns with the study's definition of PAD (stenosis >50%).<br>**Were the reference standard results interpreted without knowledge of the results of the index test? Yes.** The study design ensured that only patients in whom point-of-care DUS was performed *before* CTA were included. This temporal relationship ensures that the CTA results were interpreted without knowledge of the DUS findings.<br>**Risk of bias assessment: Low.** CTA is an appropriate reference standard, and the results were interpreted without knowledge of the index tests. | **Rationale:** CTA is a well-accepted reference standard for diagnosing PAD (>50% stenosis).<br>Were the reference standard results interpreted without knowledge of the index test?<br>**Answer:** Unclear<br>**Rationale:** The study mentions assessor blindness for point-of-care DUS before CTA, but it does not explicitly state whether CTA interpreters were blinded to DUS results.<br>**Risk of Bias:** Unclear |

| | | | | |
|---|---|---|---|---|
| | | were interpreted independently of the index test results. Therefore, the answer is "Unclear."<br>**Risk of Bias Assessment: Unclear**<br>The risk of bias for the reference standard domain is rated as "Unclear" primarily due to the lack of information about whether the CTA results were interpreted without knowledge of the index test results. While the reference standard (CTA) is likely to correctly classify PAD, and the study took steps to address potential misclassifications in calcified arteries, the uncertainty regarding blinding of the CTA interpreters introduces a potential risk of bias. Without explicit evidence of blinding, it cannot be ruled out that knowledge of the DUS results influenced the CTA interpretation, leading to an "Unclear" rating rather than "Low." | | |
| | **Error: The AI wrongly understands that if index was performed without knowing the result of standard test, then that will mean that the standard test will also be interpreted without knowing the result of the index test, which is a rationale error** | | **Error: The AI wrongly understands that if index was performed without knowing the result of standard test, then that will mean that the standard test will also be interpreted without knowing the result of the index test, which is a rationale error** | |
| Flow and timing | | | | |
| **Signaling questions:**<br><br>Was there an appropriate interval between index test(s) and reference standard? **yes**<br>Did all patients receive a reference standard? **Yes**<br>Did patients receive the same reference standard? **Yes**<br>Were all patients included in the analysis? **no** | **Answers to Signaling Questions (Flow and Timing):**<br>Was there an appropriate interval between index test(s) and reference standard? **Yes**<br>Did all patients receive a reference standard? **Yes**<br>Did patients receive the same reference standard? **Yes**<br>Were all patients included in the analysis? **Yes** | **Signaling Questions:**<br>**Was there an appropriate interval between index test(s) and reference standard?**<br>**Answer:** Yes<br>**Did all patients receive a reference standard?**<br>**Answer:** Yes<br>**Did patients receive the same reference standard?**<br>**Answer:** Yes<br>**Were all patients included in the analysis?**<br>**Answer:** Yes | | **Signaling Questions:**<br>Was there an appropriate interval between index test and reference standard?<br>**Answer:** Yes<br>**Rationale:** The mean time between point-of-care DUS and CTA was 18 days (range: 0–90 days), which is reasonable for diagnostic comparison.<br>Did all patients receive the same reference standard?<br>**Answer:** Yes<br>**Rationale:** All patients underwent CTA as the reference standard.<br>Were all patients included in the analysis? |

| | | | | |
|---|---|---|---|---|
| Risk of bias assessment **high**<br><br>**Rationale:**<br><br>The authors did mention the time between assessments was less than 90 days<br>They that all patients received the standard test, as inclusion criteria<br>They mention only one standard test<br>There is no mention or data suggesting that not all patients were analysed.<br>But they excluded patients where CTA could not reliably determine presence or absence of ≥50% stenosis due to extensive crural (distal leg) calcifications, that can induce a post hoc exclusion (for flow and timing), with a spectrum bias (unclear cases removed).<br>Thus, this exclusion makes the RoB is high | **Risk of bias (Flow and Timing domain): Low**<br>**Rationale:**<br>The **time interval** between the index test and CTA was on average **18 days** (range 0–90), and patients were **excluded if this interval exceeded 90 days** or if an intervention occurred between tests. This timing is reasonable to avoid disease progression bias.<br>**All included patients received CTA**, and **only CTA was used** as the reference standard across the study population.<br>The study **included all eligible patients (184)** who met the inclusion criteria and did not have uninterpretable CTA due to crural calcification. These exclusions were justified and described before analysis, ensuring **complete case inclusion** from the eligible group. | **Risk of Bias Assessment: Low**<br>**Rationale:**<br>**Was there an appropriate interval between index test(s) and reference standard?**<br>The article states that the mean time span between point-of-care duplex ultrasound (DUS) and computed tomography angiography (CTA) was 18 days, with a range of 0–90 days. It also specifies that patients were excluded if the time gap exceeded 90 days, ensuring that the interval between the index tests (ACC_max and AT) and the reference standard (CTA) was kept within a reasonable timeframe. Peripheral arterial disease (PAD) is generally a stable condition over short periods, and a maximum interval of 90 days is considered appropriate to minimize the risk of disease progression or changes that could affect the diagnostic comparison. Therefore, the answer is "Yes."<br>**Did all patients receive a reference standard?**<br>The study design required that all included patients underwent CTA, as it was the reference standard for defining PAD (stenosis >50%). The article confirms that patients eligible for inclusion were those who underwent both point-of-care DUS measurements and CTA. There is no indication that any included patients did not receive the reference standard. Thus, the answer is "Yes."<br>**Did patients receive the same reference standard?**<br>All patients in the study received CTA as the reference standard, specifically CTA scans of the abdominal aorta and lower extremities. The article does not mention any variation in the type or protocol of the reference standard used across patients. Therefore, the answer is "Yes," as all | | **Answer:** No<br>**Rationale:** Seventeen patients were excluded due to extensive crural calcifications, which may introduce bias.<br>**Risk of Bias:** High |

| | | | | |
|---|---|---|---|---|
| | | patients received the same reference standard.<br>**Were all patients included in the analysis?**<br>The study included 184 patients (267 legs) who met the eligibility criteria, and all were included in the analysis, as evidenced by the demographic characteristics and diagnostic accuracy results provided. The article does not report any patients being excluded from the analysis after meeting the inclusion criteria. Exclusion criteria (e.g., time gap >90 days, vascular interventions between tests, or unreliable CTA due to calcifications) were applied before inclusion, ensuring that all included patients were analyzed. Thus, the answer is "Yes."<br>**Risk of Bias Assessment: Low**<br>The risk of bias for the flow and timing domain is rated as "Low" because the study ensured an appropriate interval between the index tests and the reference standard (maximum 90 days), all patients received the same reference standard (CTA), and all included patients were accounted for in the analysis. There are no indications of differential timing, missing reference standard data, or exclusions from the analysis that would introduce bias in this domain. | | |
| | **Error: The AI did not understood that the exclusion of cases post-hoc for situations that are difficult to diagnose will cause bias.** | **Error: The AI did not understood that the exclusion of cases post-hoc for situations that are difficult to diagnose will cause bias, and that the authors mention the exclusion criteria as if they were used at the selection for the inclusion in the study time.** | | |

| **Grzybowski, 2024** | | | | |
|---|---|---|---|---|
| **Human** | **ChatGPT 4o** | **x. AI Grok 3** | **Gemini 2.0 flash** | **DeepSeek V3** |
| **Patient selection** | | | | |
| **Signaling questions:**<br>Was a consecutive or random sample of patients enrolled? **yes** | **Signaling Questions – Patient Selection Domain**<br>**Was a consecutive or random sample of patients** | **Signaling Questions for Patient Selection Domain:** | Was a consecutive or random sample of patients enrolled?<br>**Yes** | **Signaling Questions:**<br>Was a consecutive or random sample of patients enrolled?<br>**Answer:** Unclear |

| | | | | |
|---|---|---|---|---|
| Was a case-control design avoided? **Yes**<br>Did the study avoid inappropriate exclusions? **no**<br>**Risk of bias: high**<br>**Rationale:**<br><br>The authors do clearly specify consecutive patients were used<br>No case-control design. The disease was identified by 3 observers, thus no two distinct sources for cases and controls<br>The exclusion of low quality images by the AI systems can induce spectrum bias, since they are common in real life settings, and can induce spectrum bias.<br>Due to the last issue the RoB is high | **enrolled?**<br>**→ Yes**<br>**Was a case-control design avoided?**<br>**→ Yes**<br>**Did the study avoid inappropriate exclusions?**<br>**→ No**<br>**Risk of Bias (Patient Selection):**<br>**→ High**<br><br>**Rationale:**<br>The study enrolled **consecutive patients** from diabetic clinics in Poznan, Poland, which supports the answer **Yes** to the first question.<br>The design was **retrospective and comparative**, but it was **not a case-control** study; rather, it involved evaluating diagnostic tests in a screening cohort, thus **avoiding case-control design**.<br>However, **inappropriate exclusions** were present. Notably, **only patients who passed the IDx-DR quality check were included**, inherently biasing the population toward those with images of sufficient quality according to one of the index tests. Furthermore, **additional exclusions were made based on image quality from RetCAD and reference graders**, which introduces further selection bias and affects generalizability and | **Was a consecutive or random sample of patients enrolled?**<br>**Yes**<br>**Was a case-control design avoided?**<br>**Yes**<br>**Did the study avoid inappropriate exclusions?**<br>**No**<br>**Risk of Bias Assessment for Patient Selection Domain:**<br>**High**<br>**Applicability Concerns for Patient Selection Domain:**<br>**Low**<br><br>**Rationale for Answers:**<br>**Was a consecutive or random sample of patients enrolled? (Yes)**<br>The study explicitly states that retinal images were obtained from 779 consecutive patients with established diabetes mellitus at diabetic clinics in Poznan, Poland, between March 2020 and April 2021 (Page 3). The use of "consecutive patients" indicates a systematic enrollment process without selective sampling, fulfilling the criteria for a consecutive sample.<br>**Was a case-control design avoided? (Yes)**<br>The study is a retrospective comparative study that evaluates the diagnostic accuracy of two AI-based screening algorithms (IDx-DR and RetCAD) against a reference standard using a cohort of diabetic patients undergoing routine diabetic retinopathy (DR) screening. There is no indication of a case-control design where patients with known DR (cases) were compared to those without DR (controls). Instead, all patients were part of a screening population with varying DR severity, as determined by the reference standard (Page 3, Page 6).<br>**Did the study avoid inappropriate exclusions? (No)**<br>The study excluded 107 patients due to insufficient image quality, as determined by | Was a case-control design avoided? **Yes**<br>Did the study avoid inappropriate exclusions? **No**<br>Risk of bias assessment:<br>**Unclear**<br>Here's the rationale for my QUADAS-2 assessment of the patient selection domain:<br>**Was a consecutive or random sample of patients enrolled? Yes.** The study used retinal images of subjects with established diabetes mellitus that were captured at the time of DR screening. The study period was clearly defined (March 2020 and April 2021), and the images were obtained from diabetic clinics in Poznan, Poland. This suggests a consecutive enrollment of patients presenting for DR screening during that period.<br>**Was a case-control design avoided? Yes.** The study design was a retrospective comparative study, comparing the performance of two different ARIAs in screening for DR against a reference standard of human graders. It did not involve selecting patients based on the presence or absence of the outcome of interest (DR), which would be characteristic of a case-control study.<br>**Did the study avoid inappropriate exclusions? No.** The study excluded patients whose images did not have at least one disc and one macula-centered image of sufficient quality, as | **Rationale:** The study mentions retinal images were collected from diabetic clinics in Poland but does not explicitly state whether the sample was consecutive or random.<br>Was a case-control design avoided?<br>**Answer:** Yes<br>**Rationale:** The study compares two AI algorithms against a reference standard (human graders) without using a case-control design.<br>Did the study avoid inappropriate exclusions?<br>**Answer:** No<br>**Rationale:** Patients were excluded based on image quality (107 excluded by IDx-DR, 21 by RetCAD), which could introduce bias if exclusions were related to disease severity.<br>**Risk of Bias:** High |

| | | | | |
|---|---|---|---|---|
| | diagnostic accuracy estimates. | the IDx-DR quality check process, and an additional 21 patients were excluded due to insufficient quality by RetCAD, resulting in a final sample of 758 patients (Page 3, Page 6). Additionally, patients who had received prior treatment for DR (e.g., lasers or intraocular injections) were excluded (Page 3). While exclusions based on image quality are reasonable for ensuring valid AI analysis, the study notes that all images were pre-filtered by IDx-DR's quality check during enrollment, introducing a potential bias since images failing IDx-DR's quality check were not assessed by RetCAD or human graders (Page 9). This selective exclusion based on one AI system's criteria could skew the performance comparison, particularly for RetCAD, and is considered inappropriate as it may not reflect real-world scenarios where both systems would handle low-quality images independently. Furthermore, excluding treated patients may limit the generalizability to populations with a history of DR treatment, though this is less significant given the focus on screening.<br>**Risk of Bias Assessment (High)**<br>The high risk of bias is driven by the inappropriate exclusions, particularly the pre-filtering of images by IDx-DR's quality check, which introduces a selection bias affecting the fairness of the comparison between the two AI systems. The consecutive enrollment and avoidance of a case-control design are strengths, but the exclusion criteria, especially the dependency on IDx-DR's quality assessment, compromise the representativeness of the sample and the validity of the comparative analysis (Page 9). | determined by the IDx-DR quality check process. Additionally, subjects who had received treatment for DR were excluded. A total of 107 patients were excluded due to insufficient quality as determined by IDx-DR, and a further 21 were excluded due to insufficient quality by RetCAD. This exclusion based on image quality, particularly the initial exclusion based on IDx-DR, could introduce bias, as it might exclude certain types of patients or images that could affect the AI's performance.<br>**Risk of bias assessment: Unclear.** The risk of bias is rated as unclear because the exclusion of patients based on image quality, especially the initial exclusion by IDx-DR, raises concerns about whether the included sample is representative of the population for whom the test would be used. While the enrollment appears to be consecutive, the exclusions could lead to a biased selection of participants. | |
| | | | **Error: the AI missed the fact that the authors clearly specified consecutive patients, but selected consecutive nonetheless** | **Error: the AI missed the fact that the authors clearly specified consecutive patients** |
| **Index test** | | | | |

| | | | | |
|---|---|---|---|---|
| **Signaling questions:**<br><br>Were the index test results interpreted without knowledge of the results of the reference standard? **Yes**<br>If a threshold was used, was it pre-specified? **Yes**<br>Risk of bias assessment **Low**<br><br>**Rationale:**<br><br>The index test being a CNN was presented only images for assessment (non identifiable information), thus, haven't access to the standard test results (human gradings)<br>The CNNs have built in pre-specified thresholds for categorizations<br>Thus the RoB is low | **Signaling Questions – Index Test Domain**<br>**Were the index test results interpreted without knowledge of the results of the reference standard?**<br>**→ Yes**<br>**If a threshold was used, was it pre-specified?**<br>**→ Yes**<br>**Risk of Bias (Index Test):**<br>**→ Low**<br><br>**Rationale:**<br>The index test results (from both **IDx-DR** and **RetCAD**) were interpreted **automatically by the AI systems**, and the **graders were masked** to the AI outputs. This supports **blinded interpretation**.<br>Both AI systems had **pre-defined thresholds** for determining DR severity and referability. IDx-DR uses a commercial, fixed output model; RetCAD applies a continuous score with **pre-specified thresholds** for mapping to ICDR stages. | **Signaling Questions for Index Test Domain:**<br>**Were the index test results interpreted without knowledge of the results of the reference standard?**<br>**Yes**<br>**If a threshold was used, was it pre-specified?**<br>**Yes**<br>**Risk of Bias Assessment for Index Test Domain:**<br>**Low**<br>**Applicability Concerns for Index Test Domain:**<br>**Low**<br><br>**Rationale for Answers:**<br>**Were the index test results interpreted without knowledge of the results of the reference standard? (Yes)**<br>The study evaluates two automated diabetic retinopathy (DR) screening algorithms, IDx-DR and RetCAD, which are described as automated systems based on convolutional neural networks (CNNs) that generate outputs independently of human input (Page 4). The IDx-DR results were recorded during live screening, and RetCAD analysis was performed on anonymized images transferred to a cloud platform for automatic processing (Page 4). There is no indication that the AI algorithms had access to the reference standard (human grader consensus) results during their analysis. The study explicitly states that the images were evaluated independently by the IDx-DR and RetCAD algorithms (Page 1), ensuring that the index test results were generated without knowledge of the reference standard.<br>**If a threshold was used, was it pre-specified? (Yes)**<br>Both AI systems used pre-specified thresholds for classifying DR severity. For RetCAD, the study provides a clear mapping of the AI's continuous score (0 to 4.0) to | Here's the rationale for my QUADAS-2 assessment of the index test domain:<br>**Were the index test results interpreted without knowledge of the results of the reference standard? Yes.** The study clearly states that "All the graders were masked to the output of the Al and to each other's grading." This indicates that the AI's assessment of the retinal images was done independently of the human graders' assessment, which serves as the reference standard.<br>**If a threshold was used, was it pre-specified? Yes.** The thresholds for determining the severity of diabetic retinopathy (DR) using both the AI systems and the reference standard were defined based on the International Clinical Diabetic Retinopathy (ICDR) severity classification. For RetCAD, the mapping of the AI score to ICDR stages is provided in Table 2. This pre-specification of thresholds reduces the risk of bias in how the test results were interpreted.<br>**Risk of bias assessment:**<br>**Low.** Both signaling questions were answered "yes," indicating a low risk of bias. The masking of the reference standard results from the index test interpretation and the pre-specification of thresholds are strong indicators that the index | **Signaling Questions:**<br>Were the index test results interpreted without knowledge of the reference standard?<br>**Answer:** Yes<br>**Rationale:** The AI systems (index tests) analyzed images independently of human graders.<br>If a threshold was used, was it pre-specified?<br>**Answer:** Yes (for RetCAD), Unclear (for IDx-DR)<br>**Rationale:** RetCAD's thresholds (e.g., 0.00–0.49 for No DR) were predefined. IDx-DR's thresholds are not explicitly stated in the paper.<br>**Risk of Bias:** Low (RetCAD), Unclear (IDx-DR) |

| | | | | |
|---|---|---|---|---|
| | | International Clinical Diabetic Retinopathy (ICDR) stages, with specific cutoffs for no DR, mild DR, moderate DR, severe DR, and proliferative DR (Table 2, Page 5). For IDx-DR, the system outputs DR stages based on a pre-defined four-class scale, as described in the study (Page 5). The study defines referable diabetic retinopathy (RDR) as moderate non-proliferative diabetic retinopathy (NPDR) or more severe disease and/or clinically significant diabetic macular edema (CSDME), and sight-threatening diabetic retinopathy (STDR) as severe NPDR, proliferative DR, and/or CSDME (Page 4). These definitions and thresholds align with standard clinical classifications (ICDR) and were pre-specified, as evidenced by their consistent application in the analysis and reporting of sensitivity, specificity, and predictive values (Pages 6-7).<br>**Risk of Bias Assessment (Low)**<br>The index test domain has a low risk of bias because both AI systems operated independently of the reference standard, and their thresholds for DR classification were pre-specified and based on established clinical criteria (ICDR stages). There is no evidence of post-hoc threshold adjustments or influence from the reference standard results, ensuring an unbiased assessment of the index tests' performance (Pages 4-5). | tests were evaluated objectively and consistently. | |
| | | | Error: The AI inverted the blinding understanding | |
| **Reference standard** | | | | |
| **Signaling questions:**<br><br>Is the reference standard likely to correctly classify the target condition? **Yes**<br>Were the reference standard results interpreted without knowledge of the results of the index test? **Unclear** | **Signaling Questions – Reference Standard Domain**<br>**Is the reference standard likely to correctly classify the target condition?**<br>**→ Yes**<br>**Were the reference standard results interpreted without knowledge of the results of** | **Signaling Questions for Reference Standard Domain:**<br>**Is the reference standard likely to correctly classify the target condition?**<br>**Yes**<br>**Were the reference standard results interpreted without knowledge of the results of the index test?**<br>**Yes** | Is the reference standard likely to correctly classify the target condition? **Yes**<br>Were the reference standard results interpreted without knowledge of the results of the index test? **Yes**<br>Risk of bias assessment: **Low**<br>Surse și conținut conex<br>Please agrue | **Signaling Questions:**<br>Is the reference standard likely to correctly classify the target condition?<br>**Answer:** Yes<br>**Rationale:** The reference standard was consensus grading by three specialists, which is a robust method.<br>Were the reference standard results interpreted without knowledge of the index test?<br>**Answer:** Yes |

| | | | | |
|---|---|---|---|---|
| Risk of bias assessment<br>**Unclear**<br><br>**Rationale:**<br><br>The authors used the average of 3 specialists gradings with agreement coefficient, and did not use arbitration. Also the golden standard for diagnostic is biopsy, which is clearly not feasible. Furthermore the agreement coefficients are 0.54, 0,44 indicating moderate agreement, insufficient for a gold standard.<br>Blinding was clearly stated<br>Therefore the RoB remains high, due to low agreement between specialists | **the index test?**<br>**→ Yes**<br>**Risk of Bias (Reference Standard):**<br>**→ Low**<br><br>**Rationale:**<br>The **reference standard** was based on **consensus grading** by three trained human graders per image set (Polish or Indian), using the **ICDR classification system**, which is widely accepted and validated for diabetic retinopathy grading. This supports that the reference standard is likely to **correctly classify the target condition**.<br>All graders were explicitly **masked to the outputs of the AI systems** and to each other's grades, ensuring **blinded interpretation** of the reference standard. | **Risk of Bias Assessment for Reference Standard Domain:**<br>**Low**<br>**Applicability Concerns for Reference Standard Domain:**<br>**Low**<br><br>**Rationale for Answers:**<br>**Is the reference standard likely to correctly classify the target condition? (Yes)**<br>The reference standard in the study is the consensus grading by three human graders (either Polish or Indian) based on the International Clinical Diabetic Retinopathy (ICDR) severity classification, which is a widely accepted standard for diagnosing diabetic retinopathy (DR) (Page 4). The Polish graders were consultant ophthalmologists specializing in retinal diseases, and the Indian graders included two retina specialists and one certified optometrist (Page 4). The grading process involved classifying DR severity (no DR, mild NPDR, moderate NPDR, severe NPDR, proliferative DR) and identifying clinically significant diabetic macular edema (CSDME) based on surrogate markers like hard exudates (Page 4). The consensus decision, determined by the majority opinion among the three graders, enhances the reliability of the reference standard. Inter-rater agreement was assessed using Fleiss' kappa (0.44 for Polish graders, 0.54 for Indian graders) and Cohen's kappa for individual graders (ranging from 0.502 to 0.783), indicating moderate to substantial agreement (Page 4, Page 6). While the kappa values suggest some variability, the use of multiple graders and a consensus approach, combined with the standardized ICDR classification, makes the reference standard likely to correctly classify the target conditions (referable DR and sight-threatening DR). | Here's the rationale for my QUADAS-2 assessment of the reference standard domain:<br>**Is the reference standard likely to correctly classify the target condition? Yes.** The reference standard used in this study was the consensus grading by three retinal specialist graders. These graders included consultant ophthalmologists and retina specialists, who are qualified to accurately diagnose diabetic retinopathy. Using a consensus of multiple experts as the reference standard is a strong approach to minimize errors and ensure accurate classification of the presence and severity of diabetic retinopathy.<br>**Were the reference standard results interpreted without knowledge of the results of the index test? Yes.** The study explicitly states, "All the graders were masked to the output of the Al and to each other's grading." This masking ensures that the reference standard grading was conducted independently of the AI systems' results, reducing the risk of bias due to knowledge of the index test results.<br>**Risk of bias assessment: Low.** Both signaling questions received a "yes," indicating a low risk of bias in the reference standard. The use of expert graders and the masking of index test results contribute | **Rationale:** Graders were masked to AI results.<br>**Risk of Bias:** Low |

| | | | | |
|---|---|---|---|---|
| | | **Were the reference standard results interpreted without knowledge of the results of the index test? (Yes)**<br>The study explicitly states that all graders were masked to the output of the AI systems (IDx-DR and RetCAD) and to each other's grading (Page 4). The grading was performed independently, with images assessed for DR severity without access to the AI results. This blinding ensures that the reference standard results were not influenced by the index test outcomes, reducing the risk of bias in the interpretation of the reference standard.<br>**Risk of Bias Assessment (Low)**<br>The reference standard domain has a low risk of bias. The consensus grading by three experienced graders using the standardized ICDR classification is robust and likely to accurately classify DR, despite moderate inter-rater variability indicated by kappa values (Page 4). The blinding of graders to the AI results further minimizes bias, ensuring that the reference standard was applied independently of the index tests (Page 4). The combination of a reliable classification system and proper blinding supports a low risk of bias in this domain. | to the reliability and validity of the reference standard. | |
| | **Error: the AI did not consider that the human gradings had a moderate agreement coefficient, while for a gold standard higher agreement is advisable.** | **Error: the AI did not consider that the human gradings had a moderate agreement coefficient, while for a gold standard higher agreement is advisable.** | **Error: the AI did not consider that the human gradings had a moderate agreement coefficient, while for a gold standard higher agreement is advisable.** | **Error: the AI did not consider that the human gradings had a moderate agreement coefficient, while for a gold standard higher agreement is advisable.** |
| Flow and timing | | | | |
| **Signaling questions:**<br><br>Was there an appropriate interval between index test(s) and reference standard? **yes**<br>Did all patients receive a reference standard? **Yes** | **Signaling Questions – Flow and Timing Domain**<br>**Was there an appropriate interval between index test(s) and reference standard?**<br>**→ Yes** | **Signaling Questions for Flow and Timing Domain:**<br>**Was there an appropriate interval between index test(s) and reference standard?**<br>**Not Applicable**<br>**Did all patients receive a reference standard?**<br>**Yes** | Was there an appropriate interval between index test(s) and reference standard? **Not applicable**<br>Did all patients receive a reference standard? **Yes**<br>Did patients receive the same reference standard? **Yes** | **Signaling Questions:**<br>Was there an appropriate interval between index test and reference standard?<br>**Answer:** Yes<br>**Rationale:** The study was retrospective, and images were graded after AI analysis, but no significant delay is mentioned.<br>Did all patients receive the same reference standard? |

| | | | | |
|---|---|---|---|---|
| Did patients receive the same reference standard?<br>**No**<br>Were all patients included in the analysis? **no**<br>Risk of bias assessment<br>**high**<br><br>**Rationale:**<br><br>This is an assessment of the same image by AI and humans, thus no change in time can occur<br>All images were assessed by humans<br>All images were assessed by humans, but by polish and Indian<br>All images were assessed, after the initial exclusions<br>Thus, the RoB is high | **Did all patients receive a reference standard?**<br>**→ Yes**<br>**Did patients receive the same reference standard?**<br>**→ Yes**<br>**Were all patients included in the analysis?**<br>**→ No**<br>**Risk of Bias (Flow and Timing):**<br>**→ High**<br><br>**Rationale:**<br>All retinal images were obtained during a single screening visit, and both the **index tests and reference standard** were applied to these same images, meaning there was **no delay** that could affect disease status.<br>All patients **who passed image quality criteria** received the reference standard, and all were **graded using the same ICDR system**, so the **reference standard was consistently applied**.<br>However, a **substantial number of patients (107 total)** were **excluded due to image quality issues**—first by IDx-DR, then further by RetCAD and reference graders. This means **not all patients were included in the final analysis**, potentially introducing bias and affecting the applicability of the findings. | **Did patients receive the same reference standard?**<br>**No**<br>**Were all patients included in the analysis?**<br>**No**<br>**Risk of Bias Assessment for Flow and Timing Domain:**<br>**High**<br>**Applicability Concerns for Flow and Timing Domain:**<br>**Low**<br><br>**Rationale for Answers:**<br>**Was there an appropriate interval between index test(s) and reference standard? (Not Applicable)**<br>The study is a retrospective comparative analysis of retinal images collected during diabetic retinopathy (DR) screening, with the index tests (IDx-DR and RetCAD) and reference standard (human grader consensus) applied to the same set of images (Page 3). Since both the AI-based index tests and the human grading were performed on the same static dataset of fundus images, there is no temporal interval between the index tests and the reference standard. The question of an appropriate interval is therefore not applicable in this context, as the study design does not involve sequential testing over time.<br>**Did all patients receive a reference standard? (Yes)**<br>All 758 patients included in the final analysis received the reference standard, which was the consensus grading by three human graders (either Polish or Indian) based on the International Clinical Diabetic Retinopathy (ICDR) classification (Page 4, Page 6). The study confirms that the images from these patients were graded by the reference standard graders, with no patients excluded from grading after meeting the inclusion criteria and passing the image quality checks (Page 6). | Were all patients included in the analysis? **No**<br>Risk of bias assessment: **Unclear** | **Answer:** No<br>**Rationale:** Patients were split into two grading groups (Polish and Indian graders), which could introduce variability.<br>Were all patients included in the analysis?<br>**Answer:** No<br>**Rationale:** Exclusions due to image quality (128 patients total) may affect generalizability.<br>**Risk of Bias:** High |

| | | | | |
|---|---|---|---|---|
| | | **Did patients receive the same reference standard? (No)**<br>While all patients were graded using the same ICDR classification system, the reference standard was applied by two different groups of graders: 320 patients were graded by three Polish consultant ophthalmologists, and 438 patients were graded by three Indian graders (two retina specialists and one certified optometrist) (Page 4). The study acknowledges this split, noting that it was due to financial constraints, and reports different inter-rater agreement levels (Fleiss' kappa of 0.44 for Polish graders and 0.54 for Indian graders) (Page 4). The use of two different grading teams with varying expertise and agreement levels introduces variability in the reference standard application, meaning that not all patients received the *same* reference standard in terms of grader composition and consistency.<br>**Were all patients included in the analysis? (No)**<br>The study initially included 779 consecutive patients, but 107 were excluded due to insufficient image quality as determined by IDx-DR, 21 additional patients were excluded due to insufficient quality by RetCAD, and 2 more were excluded due to insufficient quality by the reference graders, resulting in a final sample of 758 patients (Page 3, Page 6). While the exclusions based on image quality are justified to ensure valid AI and human grading, the fact that not all enrolled patients were included in the final analysis (21 patients, or approximately 2.7% of the initial cohort, were excluded) means that this signaling question is answered as "No." Additionally, the pre-filtering by IDx-DR's quality check may have disproportionately affected the inclusion of patients for RetCAD analysis (Page 9).<br>**Risk of Bias Assessment (High)**<br>The flow and timing domain has a high risk | | |

| | | | | |
|---|---|---|---|---|
| | | of bias primarily due to the use of different reference standard grading teams (Polish vs. Indian graders), which introduces variability in the reference standard's application and could affect the consistency of the ground truth (Page 4). Additionally, the exclusion of some patients from the analysis (due to image quality issues, particularly influenced by IDx-DR's quality check) further contributes to the risk of bias, as it may skew the performance comparison between the two AI systems (Page 9). While the lack of a temporal interval is not a concern (as it is not applicable), the combination of differential reference standards and incomplete inclusion of all enrolled patients warrants a high risk of bias assessment. | | |
| | **Error: The AI did not spot that there were two teams of humans, with different agreement between them, that graded the images, thus two standard tests were used** | We considered that not applicable represents the same meaning as yes for the first question. | We considered that not applicable represents the same meaning as yes for the first question.<br>**Error: The AI did not spot that there were two teams of humans, with different agreement between them, that graded the images, thus two standard tests were used** | **Error: The AI states that no significant delay is mentioned in the text by the authors, but the images are assessed by an algorithm and by a human, thus a delay cannot exist.** |

| **dos Reis, 2024** | | | | |
|---|---|---|---|---|
| **Human** | **ChatGPT 4o** | **x.AI Grok 3** | **Gemini 2.0 flash** | **DeepSeek v3** |
| **Patient selection** | | | | |
| **Signaling questions:**<br>Was a consecutive or random sample of patients enrolled? **unclear**<br>Was a case-control design avoided? **Yes**<br>Did the study avoid inappropriate exclusions? **No**<br>**Risk of bias: High**<br>**Rationale:** | **Answers to Signaling Questions (Patient Selection domain):**<br>**Was a consecutive or random sample of patients enrolled?**<br>**Answer:** *No*<br>**Was a case-control design avoided?**<br>**Answer:** *Yes*<br>**Did the study avoid inappropriate exclusions?**<br>**Answer:** *Yes* | **Signaling Questions:**<br>**Was a consecutive or random sample of patients enrolled?**<br>**Answer:** Yes<br>**Was a case-control design avoided?**<br>**Answer:** Yes<br>**Did the study avoid inappropriate exclusions?**<br>**Answer:** Yes<br>**Risk of Bias: Low**<br>**Rationale:**<br>**Question 1:** The article specifies that a "convenience sample with consecutive | Okay, I will provide the answers to the signaling questions and the overall risk of bias for the "Patient Selection" domain.<br>Was a consecutive or random sample of patients enrolled? **No**<br>Was a case-control design avoided? **Yes**<br>Did the study avoid inappropriate exclusions? **No** | *Domain 1: Patient Selection*<br><br>**Signaling Questions:**<br>Was a consecutive or random sample of patients enrolled?<br>**Answer:** Yes<br>Was a case-control design avoided?<br>**Answer:** Yes<br>Did the study avoid inappropriate exclusions?<br>**Answer:** Yes<br>**Risk of Bias:** Low |

| | | | | |
|---|---|---|---|---|
| The authors do clearly specify that a convenience sample with consecutive patients which is not clear<br>No case-control design.<br>The disease was identified observers, thus no two distinct sources for cases and controls<br>The exclusion of low quality images can induce spectrum bias, since they are common in real life settings, and can induce spectrum bias.<br>Due to the last issue the RoB is high | **Risk of bias (Patient Selection domain):**<br>**Answer:** *High*<br>**Rationale:**<br>**Consecutive or random sampling:**<br>The study explicitly used a *convenience sample with consecutive patients*, which is not equivalent to true random or fully consecutive sampling across all datasets, and it may introduce selection bias.<br>**Case-control design avoidance:**<br>The study was not structured as a case-control design. It involved retrospective and prospective collection of real-world images across diverse healthcare settings.<br>**Avoidance of inappropriate exclusions:**<br>The study excluded images due to poor quality, which is justifiable and explicitly described using objective criteria and automated tools (e.g., EyeQ algorithm). These exclusions were appropriate for ensuring diagnostic performance reliability.<br>**Overall risk of bias:**<br>The use of a convenience sample, especially across multiple datasets without clear randomization or systematic enrollment procedures, increases the risk of bias in patient selection, leading to a **high** risk rating for this domain. | patients was used" across multiple datasets from three public and academic institutions (Page 2, Methods, Datasets). This indicates consecutive enrollment, satisfying the requirement for a non-random but systematic sampling approach.<br>**Question 2:** The study does not employ a case-control design. It includes a mixed population of patients with and without diabetic retinopathy (DR) from primary and specialized care settings, assessed prospectively and retrospectively, ensuring a cohort-based approach (Page 2, Methods, Datasets).<br>**Question 3:** Exclusions were restricted to uninterpretable images due to poor quality (e.g., overexposure, inadequate focus, insufficient lighting, excessive artifacts), which is appropriate for ensuring the reliability of diagnostic accuracy (Page 3, Grading). No exclusions based on disease severity, patient demographics, or other inappropriate criteria were reported, supporting the appropriateness of the exclusion criteria. | Risk of bias assessment: **High**<br><br>Here's the rationale for my answers to the "Patient Selection" domain questions:<br>**Was a consecutive or random sample of patients enrolled? No.** The study used a convenience sample of patients. Specifically, it states, "A convenience sample with consecutive patients was used". While patients were consecutively sampled, the overall sampling method was a convenience sample, which is not the same as a random sample.<br>**Was a case-control design avoided? Yes.** The study used a cross-sectional design, collecting data on patients undergoing color fundus photography (CFP). It did not compare patients with a known outcome (cases) to those without the outcome (controls) to determine exposure, which would be a case-control study.<br>**Did the study avoid inappropriate exclusions? No.** The study excluded a significant number of images based on quality. They state, "all images were assessed for quality by the ophthalmologists and the images with interference factors, such as overexposure, inadequate focus, insufficient lighting, and excessive artifacts, were discarded". Additionally, the EyeQ | |

| | | | | |
|---|---|---|---|---|
| | | | algorithm was used to exclude images of insufficient quality. This exclusion of a large number of images (38,175 excluded by EyeQ and 1,370 not gradable by ophthalmologist) could introduce bias if the image quality is related to the presence or severity of diabetic retinopathy.<br>**Risk of bias assessment:**<br>**High.** The use of a convenience sample and the exclusion of a large number of images due to quality concerns contribute to a high risk of bias. These factors may limit the generalizability of the study findings and could potentially distort the accuracy of the diagnostic test being evaluated. | |
| | **Error: the AI did not consider the exclusion of low quality images as an error for patient selection, inducing bias**<br>**The AI in a situation where the sampling is unclear (authors mentioned a convenience sample with consecutive patients), states one of the two.** | **Error: the AI did not consider the exclusion of low quality images as an error for patient selection, inducing bias**<br>**The AI in a situation where the sampling is unclear (authors mentioned a convenience sample with consecutive patients), states one of the two.** | **Error: The AI in a situation where the sampling is unclear (authors mentioned a convenience sample with consecutive patients), states one of the two.** | **Error: the AI did not consider the exclusion of low quality images as an error for patient selection, inducing bias**<br>**The AI in a situation where the sampling is unclear (authors mentioned a convenience sample with consecutive patients), states one of the two.** |
| **Index test** | | | | |
| **Signaling questions:**<br><br>Were the index test results interpreted without knowledge of the results of the reference standard?<br>**Yes** | **Answers to Signaling Questions (Index Test domain):**<br>**Were the index test results interpreted without knowledge of the results of the reference standard?**<br>**Answer:** *Yes* | **Signaling Questions:**<br>**Were the index test results interpreted without knowledge of the results of the reference standard?**<br>**Answer:** Yes<br>**If a threshold was used, was it pre-specified?**<br>**Answer:** Yes | Okay, here are the answers to the signaling questions and the risk of bias assessment for the "Index Test" domain:<br>Were the index test results interpreted without knowledge | **Signaling Questions:**<br>Were the index test results interpreted without knowledge of the reference standard?<br>**Answer:** Yes<br>If a threshold was used, was it pre-specified?<br>**Answer:** Yes<br>**Risk of Bias:** Low<br>**Rationale:** |

| | | | | |
|---|---|---|---|---|
| If a threshold was used, was it pre-specified? **No**<br>Risk of bias assessment **Unclear**<br><br>**Rationale:**<br><br>The authors clearly specified that the reference results were not available for the index test<br>No prespecified thresholds were used, post-hoc ones were tested<br>Thus an unclear RoB exists | **If a threshold was used, was it pre-specified?**<br>**Answer:** *No*<br>**Risk of bias (Index Test domain):**<br>**Answer:** *Unclear*<br>**Rationale:**<br>**Blinding to reference standard:**<br>The manuscript states that *clinical information and reference standard results were not available to the performers/readers of the index test*, indicating proper blinding and reducing potential for interpretive bias.<br>**Pre-specified threshold:**<br>The study reports evaluating three operational cut-off points (e.g., for high sensitivity, high specificity, and maximum gain), suggesting these were selected post hoc based on performance metrics rather than being pre-specified before model testing.<br>**Overall risk of bias:**<br>Despite proper blinding, the absence of a clearly pre-specified threshold and the use of multiple thresholds evaluated retrospectively leads to an **unclear** risk of bias for this domain. | **Risk of Bias: Low**<br>**Rationale:**<br>**Question 1:** The article clearly states that "clinical information and reference standard results were not available to the performers/readers of the index test" (Page 3, Neural network model). This confirms that the machine learning algorithm's results were interpreted independently of the reference standard, ensuring blinding.<br>**Question 2:** The study pre-specified three operational cutoff points for the algorithm's performance evaluation: high specificity, high sensitivity, and maximum gain point (Page 4, Statistical analysis). These thresholds were consistently applied in the results section (Page 5, Results), indicating they were established before analysis. | of the results of the reference standard? **Yes**<br>If a threshold was used, was it pre-specified? **No**<br>Risk of bias assessment: **Unclear**<br><br>Here's the rationale for my answers to the "Index Test" domain questions:<br>**Were the index test results interpreted without knowledge of the results of the reference standard? Yes.** The authors clearly state, "Clinical information and reference standard results were not available to the performers/readers of the index test". This indicates that the interpretation of the AI algorithm's results was done without knowledge of the ophthalmologists' diagnoses.<br>**If a threshold was used, was it pre-specified? No.** The study mentions using three operational cutoff points for the algorithm's output: "with high specificity, with greater sensitivity and maximum gain point". However, it does not explicitly state that these cutoff points were defined *before* the analysis. The absence of a clear statement about pre-specification makes it unclear whether these thresholds were chosen post hoc, based on the data.<br>**Risk of bias assessment: Unclear.** While the study adequately addressed blinding by ensuring that the index test | The algorithm (index test) was trained and evaluated independently of the ophthalmologists' grading (reference standard).<br>Thresholds (e.g., high sensitivity vs. high specificity) were pre-defined for ROC analysis. |

| | | | | |
|---|---|---|---|---|
| | | | was interpreted without knowledge of the reference standard, the lack of clarity regarding the pre-specification of the thresholds introduces some uncertainty. If the thresholds were chosen after seeing the data, it could lead to bias in favor of the AI algorithm's performance. Therefore, the risk of bias is assessed as "unclear." | |
| | | **Error: The IA considered the "three operational cutoff points for the algorithm's performance evaluation: high specificity, high sensitivity, and maximum gain point" as a prespecified thresholds, but they are in fact post-hoc decisions.** | | **Error: The IA considered the "three operational cutoff points for the algorithm's performance evaluation: high specificity, high sensitivity, and maximum gain point" as a prespecified thresholds, but they are in fact post-hoc decisions.** |
| **Reference standard** | | | | |
| **Signaling questions:**<br><br>Is the reference standard likely to correctly classify the target condition? **Yes**<br>Were the reference standard results interpreted without knowledge of the results of the index test? **yes**<br>Risk of bias assessment **low**<br><br>**Rationale:**<br><br>The authors used the average of 2 specialists gradings, with a third in case of disagreement, using international guidelines, with excellent agreement coefficient between raters. Also the golden standard for | **Answers to Signaling Questions (Reference Standard domain):**<br>**Is the reference standard likely to correctly classify the target condition?**<br>**Answer:** *Yes*<br>**Were the reference standard results interpreted without knowledge of the results of the index test?**<br>**Answer:** *Yes*<br>**Risk of bias (Reference Standard domain):**<br>**Answer:** *Low*<br><br>Rationale:<br><br>**Correct classification by reference standard:**<br>The reference standard was based on the *majority* | **Signaling Questions:**<br>**Is the reference standard likely to correctly classify the target condition?**<br>**Answer:** Yes<br>**Were the reference standard results interpreted without knowledge of the results of the index test?**<br>**Answer:** Yes<br>**Risk of Bias: Low**<br>**Rationale:**<br>**Question 1:** The reference standard involves classification by two ophthalmologists using the International Clinical Diabetic Retinopathy (ICDR) scale, with disagreements resolved by a third ophthalmologist (Page 3, Grading). The ICDR scale is a widely accepted standard for diabetic retinopathy (DR) classification, and the reported kappa value of 0.813 indicates near-perfect agreement, supporting the reference standard's ability to correctly classify DR.<br>**Question 2:** The article explicitly notes that "clinical information and reference standard | Okay, I will now answer the questions for the "Reference Standard" domain:<br>Is the reference standard likely to correctly classify the target condition? **Yes**<br>Were the reference standard results interpreted without knowledge of the results of the index test? **Yes**<br>Risk of bias assessment: **Low**<br>Surse și conținut conex<br>please argue<br>Here's the rationale for my answers to the "Reference Standard" domain questions:<br>**Is the reference standard likely to correctly classify the target condition? Yes.** The reference standard used in this study was the combined decision of three ophthalmologists, which is a strong and accepted method | **Signaling Questions:**<br>Is the reference standard likely to correctly classify the target condition?<br>**Answer:** Yes<br>Were the reference standard results interpreted without knowledge of the index test?<br>**Answer:** Yes<br>**Risk of Bias:** Low<br>**Rationale:**<br>The reference standard was the consensus of three ophthalmologists using the ICDR scale, a validated method for DR diagnosis. Ophthalmologists graded images blinded to the algorithm's results. |

| | | | | |
|---|---|---|---|---|
| diagnostic is biopsy, which is clearly not feasible.<br>Blinding was clearly stated<br>Therefore the RoB remains low | *decision of two independent ophthalmologists*, with adjudication by a third in cases of disagreement. The use of the ICDR (International Clinical Diabetic Retinopathy) scale is consistent with accepted clinical guidelines and has strong validity.<br>**Blinding to index test results:**<br>The study clearly states that *clinical information and reference standard results were not available to the assessors of the index test*, implying effective blinding of the reference standard assessment to index test results.<br>**Overall risk of bias:**<br>The combination of an established classification system, expert reviewers, and proper blinding indicates a **low** risk of bias in this domain. | results were not available to the assessors of the reference standard" (Page 3, Grading). This ensures that the ophthalmologists' classifications were made without knowledge of the index test (machine learning algorithm) results, confirming blinding. | for diagnosing diabetic retinopathy. Ophthalmologists are trained to identify and grade DR, making their consensus a reliable standard for determining the presence and severity of the condition.<br>**Were the reference standard results interpreted without knowledge of the results of the index test? Yes.** The study explicitly states, "Clinical information and reference standard results were not available to the assessors of the reference standard." This blinding is crucial to prevent bias in the evaluation of the reference standard.<br>**Risk of bias assessment: Low.** Since the reference standard is a reliable method (expert ophthalmologists' consensus) and was interpreted without knowledge of the index test results, the risk of bias for the reference standard is considered low. | |
| | **Error: the AI considered that if assessors of the index test are unaware of the results of the standard test, then this means the assessors of the standard test are unaware of the results of the index test, which is not the case.** | | | |
| Flow and timing | | | | |
| **Signaling questions:**<br><br>Was there an appropriate interval between index | **Answers to Signaling Questions (Flow and Timing domain):**<br>**Was there an appropriate interval between index** | **Signaling Questions:**<br>**Was there an appropriate interval between index test(s) and reference standard?**<br>**Answer:** Not Applicable | Okay, I will provide the answers to the signaling questions and the risk of bias assessment for the "Flow and Timing" domain. | **Signaling Questions:**<br>Was there an appropriate interval between index test and reference standard?<br>**Answer:** Unclear |

| | | | | |
|---|---|---|---|---|
| test(s) and reference standard? **yes**<br>Did all patients receive a reference standard? **Yes**<br>Did patients receive the same reference standard? **Yes**<br>Were all patients included in the analysis? **No**<br>Risk of bias assessment **High**<br><br>**Rationale:**<br><br>This is an assessment of the same image by AI and humans, thus no change in time can occur<br>All images were assessed by humans<br>All images were assessed by humans<br>All images were assessed, after the initial exclusions<br>Thus, the RoB is high due to exclusions | **test(s) and reference standard?**<br>**Answer:** *Yes*<br>**Did all patients receive a reference standard?**<br>**Answer:** *Yes*<br>**Did patients receive the same reference standard?**<br>**Answer:** *Yes*<br>**Were all patients included in the analysis?**<br>**Answer:** *Yes*<br>**Risk of bias (Flow and Timing domain):**<br>**Answer:** *Low*<br><br>Rationale:<br><br>**Timing between tests:**<br>The reference standard and index test were applied to the *same retinal images* rather than separate clinical encounters, eliminating concerns about time-based disease progression or regression.<br>**Use of reference standard across all patients:**<br>All included images were graded by ophthalmologists using the same reference standard protocol, indicating complete application.<br>**Uniformity of reference standard:**<br>The same ICDR-based classification was applied by trained ophthalmologists across the dataset, ensuring consistency.<br>**Inclusion in analysis:**<br>All patients with gradable | **Did all patients receive a reference standard?**<br>**Answer:** Yes<br>**Did patients receive the same reference standard?**<br>**Answer:** Yes<br>**Were all patients included in the analysis?**<br>**Answer:** No<br>**Risk of Bias: High**<br>**Rationale:**<br>**Question 1:** The interval between the index test and reference standard is not applicable because both the index test (machine learning algorithm) and the reference standard (ophthalmologist classification) were performed on the same set of color fundus photographs (CFPs) with no temporal delay (Page 3, Grading; Page 3, Neural network model).<br>**Question 2:** All patients included in the analysis received the reference standard, as the study used ophthalmologist classification of CFPs for all gradable images (Page 3, Grading).<br>**Question 3:** All patients received the same reference standard, which was the classification of CFPs by two ophthalmologists (with a third for adjudication) using the International Clinical Diabetic Retinopathy (ICDR) scale (Page 3, Grading).<br>**Question 4:** Not all patients were included in the analysis. The study started with 5308 patients and 55,371 CFPs, but after excluding uninterpretable images due to poor quality, only 4590 patients and 15,816 CFPs (28.5% of the original images) were analyzed (Page 4, Results). The substantial exclusion rate (71.5% of CFPs) raises concerns about potential bias, as these exclusions may not be random and could affect the representativeness of the results. | Was there an appropriate interval between index test(s) and reference standard? **Yes**<br>Did all patients receive a reference standard? **Yes**<br>Did patients receive the same reference standard? **Yes**<br>Were all patients included in the analysis? **No**<br>Risk of bias assessment: **Unclear**<br>Surse și conținut conex<br>Please argue<br>Here's the rationale for my answers to the "Flow and Timing" domain questions:<br>**Was there an appropriate interval between index test(s) and reference standard? Yes.** The study design involved using the AI algorithm on the same set of fundus photographs that were evaluated by the ophthalmologists. This implies that the index test (AI algorithm) and the reference standard (ophthalmologists' evaluation) were performed on the same images, effectively simultaneously. Thus, there is no concern about an inappropriate interval influencing the comparison.<br>**Did all patients receive a reference standard? Yes.** All images included in the analysis were assessed by both the index test (AI algorithm) and the reference standard (ophthalmologists). The reference standard was applied to all images that passed the | Did all patients receive the same reference standard?<br>**Answer:** Yes<br>Were all patients included in the analysis?<br>**Answer:** Yes<br>**Risk of Bias:** Low<br>**Rationale:**<br>The interval between image acquisition and grading was not specified, but the retrospective/prospective design suggests minimal delay.<br>All included images underwent the same reference standard (ophthalmologist grading).<br>Exclusions were clearly documented (uninterpretable images), and the analysis included all eligible cases. |

| | | | | |
|---|---|---|---|---|
| | images were analyzed. Although ungradable images were excluded, this was done based on predefined and appropriate quality criteria, and remaining patients/images were included.<br>**Overall risk of bias:** The consistent application of tests and inclusion of all eligible subjects/images in the final analysis leads to a **low** risk of bias. | | quality assessment and were included in the final analysis.<br>**Did patients receive the same reference standard? Yes.** The reference standard was the combined decision of three ophthalmologists, applied uniformly to all images. This ensured that all patients were evaluated using the same standard, reducing the risk of bias due to variations in the reference standard.<br>**Were all patients included in the analysis? No.** A significant number of images were excluded from the analysis due to poor quality. Specifically, 38,175 images were excluded by the EyeQ quality filter, and 1,370 were deemed ungradable by the ophthalmologists. This substantial exclusion means that not all initially included patients/images were part of the final analysis.<br>**Risk of bias assessment: Unclear.** While the index test and reference standard were applied appropriately with no time interval and the same standard used for all, the significant exclusion of images raises concerns. If the exclusion of images was related to the likelihood of detecting the target condition (diabetic retinopathy), it could introduce bias. Since the potential for bias is present but the degree to which it affects the results is unclear, the risk | |

| | | | | |
|---|---|---|---|---|
| | | | of bias for this domain is assessed as "unclear." | |
| | **Error: the AI did not took into account the high amount of exclusions due to apparently poor image quality** | Not applicable can be considered similar to yes in this case | | **Error: the AI did not took into account the high amount of exclusions due to apparently poor image quality** |

| **Muntean, 2025** | | | | |
|---|---|---|---|---|
| **Human** | **ChatGPT 4o** | **x. AI Grok 3** | **Gemini 2.0 flash** | **DeepSeek V3** |
| **Patient selection** | | | | |
| **Signaling questions:**<br>Was a consecutive or random sample of patients enrolled?<br>Was a case-control design avoided?<br>Did the study avoid inappropriate exclusions?<br>**Risk of bias:** | **Answers (QUADAS-2: Domain 1 – Patient Selection):**<br>**Was a consecutive or random sample of patients enrolled? → No**<br>**Was a case-control design avoided? → Yes**<br>**Did the study avoid inappropriate exclusions? → Unclear**<br>**Risk of bias: → High**<br>**Rationale:**<br>The study is a **narrative literature review**, not a primary diagnostic accuracy study, and therefore **did not enroll individual patients**, let alone through random or consecutive sampling.<br>A **case-control design was not employed**, as this was a synthesis of previously published research.<br>It is **unclear** whether inappropriate exclusions were avoided, as the study did not provide detailed justification for the exclusion of 28 articles (from 75 eligible to 47 included), nor did it specify | The QUADAS-2 tool is designed to assess the risk of bias and applicability in studies evaluating the diagnostic accuracy of tests. However, the provided article, "Impact of COVID-19 pandemic on physical activity levels and mental health in university students" by Muntean et al. (2025), is a literature review, not a diagnostic accuracy study. QUADAS-2 is not applicable to literature reviews, as it is tailored for primary studies assessing diagnostic tests against a reference standard.<br><br>QUADAS-2 Assessment<br><br>Since the article is a literature review, the QUADAS-2 domains (Patient Selection, Index Test, Reference Standard, Flow and Timing) cannot be applied. Below, I provide the responses as requested, noting that all domains are "not applicable" due to the study type.<br><br>*Domain 1: Patient Selection*<br><br>**Signaling Questions**: Not applicable.<br>**Risk of Bias**: Not applicable.<br>**Applicability Concerns**: Not applicable. | Here are the answers to the signaling questions and the risk of bias assessment for the "Patient Selection" domain:<br>Was a consecutive or random sample of patients enrolled? **No**<br>Was a case-control design avoided? **Yes**<br>Did the study avoid inappropriate exclusions? **Unclear**<br>Risk of bias: **Unclear**<br>**Rationale:**<br>**Was a consecutive or random sample of patients enrolled? No.** The study is a literature review. The authors selected studies based on specific criteria: publication date (2020-2024) and keywords ("COVID-19", "pandemic", "physical activity", and "university students"). They performed a manual search in electronic databases (PubMed, Scopus, and Web of Science). This selection process is not a consecutive or random sample of patients but a selection of studies, which is different from | **Signaling Question 1:** Was a consecutive or random sample of patients enrolled?<br>**Answer:** No<br>**Signaling Question 2:** Was a case-control design avoided?<br>**Answer:** Yes<br>**Signaling Question 3:** Did the study avoid inappropriate exclusions?<br>**Answer:** Unclear<br>**Risk of Bias:** High<br>**Rationale:**<br>The study is a literature review, not a primary study involving patient enrollment, so consecutive or random sampling does not apply (No).<br>It avoids a case-control design by synthesizing existing studies (Yes).<br>The exclusion criteria (e.g., non-English studies, high school students) may introduce bias, but details are insufficient to judge appropriateness (Unclear). |

| | | | | |
|---|---|---|---|---|
| | whether exclusions were based on risk of bias or relevance. | *Domain 2: Index Test*<br><br>**Signaling Questions**: Not applicable.<br>**Risk of Bias**: Not applicable.<br>**Applicability Concerns**: Not applicable.<br><br>*Domain 3: Reference Standard*<br><br>**Signaling Questions**: Not applicable.<br>**Risk of Bias**: Not applicable.<br>**Applicability Concerns**: Not applicable.<br><br>*Domain 4: Flow and Timing*<br><br>**Signaling Questions**: Not applicable.<br>**Risk of Bias**: Not applicable.<br>**Applicability Concerns**: Not applicable.<br><br>Rationale<br><br>The QUADAS-2 tool evaluates bias in studies where a diagnostic test (index test) is compared to a reference standard to determine accuracy (e.g., sensitivity, specificity). The article by Muntean et al. is a narrative literature review synthesizing findings from 47 studies on the impact of COVID-19 on university students' physical activity and mental health. It does not involve a diagnostic test, a reference standard, or patient selection for diagnostic purposes. Instead, it summarizes observational data, primarily from surveys, on lifestyle and health outcomes.<br><br>**Patient Selection**: The review focuses on university students aged 18–22 but does not involve selecting patients for diagnostic testing. The inclusion criteria (e.g., studies from 2020–2024, English language) relate to literature selection, not patient enrollment, making this domain irrelevant. | patient selection in a primary study.<br>**Was a case-control design avoided? Yes.** The study is a literature review that includes various study designs. It is not a primary study with a case-control design. The review analyzed 47 studies.<br>**Did the study avoid inappropriate exclusions? Unclear.** The authors mention exclusion criteria such as publication language (other than English), publication year (before 2020), study type (systematic reviews, meta-analyses), and the population studied (high school students). It is unclear if these exclusions were appropriate or if they introduced bias. The rationale for excluding non-English articles, for instance, might be a source of bias.<br>**Risk of bias: Unclear.** Because the selection of studies for the review was not random or consecutive and it is unclear if the exclusions were appropriate, the risk of bias for patient selection is rated as unclear. | |

| | | | | |
|---|---|---|---|---|
| | | **Index Test**: No diagnostic test is evaluated. The review discusses physical activity levels and mental health outcomes, often measured via surveys or fitness trackers, not as diagnostic tools.<br>**Reference Standard**: There is no reference standard for diagnosing a condition, as the review examines trends in behavior and health, not diagnostic accuracy.<br>**Flow and Timing**: This domain assesses the timing of tests and patient flow in diagnostic studies. The review does not involve patient follow-up or test administration, rendering this domain inapplicable.<br><br>For literature reviews, tools like AMSTAR-2 or ROBIS are more appropriate to assess methodological quality or bias. If you have specific signaling questions or prefer an alternative assessment (e.g., AMSTAR-2), please provide them, and I can adapt the evaluation accordingly.<br><br>Here are the answers to the signaling questions and the risk of bias assessment for the "Patient Selection" domain:<br>Was a consecutive or random sample of patients enrolled? **No**<br>Was a case-control design avoided? **Yes**<br>Did the study avoid inappropriate exclusions? **Unclear**<br>Risk of bias: **Unclear**<br>**Rationale:**<br>**Was a consecutive or random sample of patients enrolled? No.** The study is a literature review. The authors selected studies based on specific criteria: publication date (2020-2024) and keywords ("COVID-19", "pandemic", "physical activity", and "university students"). They performed a manual search in electronic databases (PubMed, Scopus, and Web of Science). This selection process is not a consecutive or random sample of patients but a selection of | | |

| | | | | |
|---|---|---|---|---|
| | | studies, which is different from patient selection in a primary study.<br>**Was a case-control design avoided? Yes.** The study is a literature review that includes various study designs. It is not a primary study with a case-control design. The review analyzed 47 studies.<br>**Did the study avoid inappropriate exclusions? Unclear.** The authors mention exclusion criteria such as publication language (other than English), publication year (before 2020), study type (systematic reviews, meta-analyses), and the population studied (high school students). It is unclear if these exclusions were appropriate or if they introduced bias. The rationale for excluding non-English articles, for instance, might be a source of bias.<br>**Risk of bias: Unclear.** Because the selection of studies for the review was not random or consecutive and it is unclear if the exclusions were appropriate, the risk of bias for patient selection is rated as unclear. | | |
| | | | | |
| **Index test** | | | | |
| **Signaling questions:**<br><br>Were the index test results interpreted without knowledge of the results of the reference standard?<br>If a threshold was used, was it pre-specified?<br>Risk of bias assessment | **Answers (QUADAS-2: Domain 2 – Index Test):**<br>**Were the index test results interpreted without knowledge of the results of the reference standard? → Not applicable**<br>**If a threshold was used, was it pre-specified? → Not applicable**<br>**Risk of bias: → Not applicable**<br>**Rationale:**<br>This study is a **literature review**, not a diagnostic accuracy study. Therefore, it does **not include an index test**, nor does it assess or compare test performance against a reference standard. | *Signaling Questions and Answers*<br><br>**Were the index test results interpreted without knowledge of the results of the reference standard?**<br>**Answer**: Not applicable.<br>**If a threshold was used, was it pre-specified?**<br>**Answer**: Not applicable.<br><br>*Risk of Bias for Index Test*<br><br>**Risk of Bias**: Not applicable.<br><br>*Applicability Concerns for Index Test*<br><br>**Applicability Concerns**: Not applicable. | Okay, I will now assess the "Index Test" domain. Since this is a literature review, the "index test" refers to the primary studies included in the review.<br>Were the index test results interpreted without knowledge of the results of the reference standard?<br>If a threshold was used, was it pre-specified?<br>Risk of bias assessment<br>Surse și conținut conex<br>Please argue<br>Okay, here's the assessment of the "Index Test" domain:<br>Were the index test results interpreted without knowledge | **Signaling Question 1:** Were the index test results interpreted without knowledge of the reference standard?<br>**Answer:** Not applicable<br>**Signaling Question 2:** If a threshold was used, was it pre-specified?<br>**Answer:** Not applicable<br>**Risk of Bias:** Unclear<br>**Rationale:**<br>QUADAS-2 is designed for diagnostic accuracy studies, whereas this article is a literature review. The "index test" domain is not applicable here. |

| | | | | |
|---|---|---|---|---|
| | Since no diagnostic test was used or evaluated, the signaling questions and risk of bias rating for this domain are **not applicable**. | Rationale<br><br>The article by Muntean et al. (2025), titled "Impact of COVID-19 pandemic on physical activity levels and mental health in university students," is a literature review synthesizing findings from 47 studies. The QUADAS-2 tool, specifically the Index Test domain, is designed to assess bias in primary diagnostic accuracy studies where an index test (the diagnostic test under evaluation) is compared to a reference standard. This domain is not applicable to a literature review for the following reasons:<br><br>**Were the index test results interpreted without knowledge of the results of the reference standard?**<br>This question evaluates whether the interpretation of a diagnostic test (index test) was blinded to the results of the reference standard to avoid bias. The article does not involve a diagnostic index test. Instead, it reviews studies that primarily used surveys, wearable fitness trackers, or self-reported measures to assess physical activity levels and mental health outcomes (e.g., anxiety, depression). These measures are not diagnostic tests, and there is no reference standard for comparison in the context of diagnostic accuracy. Thus, this question is irrelevant to the study design.<br>**If a threshold was used, was it pre-specified?**<br>This question applies to diagnostic studies where a threshold (e.g., a cutoff value for a test result) is used to classify outcomes (e.g., positive or negative for a condition). The reviewed studies did not employ diagnostic thresholds; they reported outcomes like changes in physical activity levels (e.g., steps per day, minutes of exercise) or mental health scores (e.g., anxiety or depression scales) without diagnostic cutoffs. The | of the results of the reference standard? **Not applicable**<br>If a threshold was used, was it pre-specified? **Not applicable**<br>Risk of bias assessment: **Not applicable**<br>**Rationale:**<br>**Were the index test results interpreted without knowledge of the results of the reference standard? Not applicable.** In a literature review, the authors are analyzing already published studies. They are not directly interpreting "index test" results (i.e., the measurements or assessments of physical activity and mental health) in individual patients and comparing them to a "reference standard." This concept doesn't apply in the same way as it does in a primary diagnostic accuracy study. The authors of the review extract and synthesize findings from the included studies.<br>**If a threshold was used, was it pre-specified? Not applicable.** Similarly, the concept of a "pre-specified threshold" for an index test is not applicable to a literature review. The review summarizes the findings of other studies, which may or may not have used thresholds. The review itself does not involve applying thresholds to index test results.<br>**Risk of bias assessment: Not applicable.** Since the signaling questions are not applicable, | |

| | | | | |
|---|---|---|---|---|
| | | literature review itself does not define or analyze thresholds, making this question inapplicable.<br><br>**Risk of Bias and Applicability**: The Index Test domain of QUADAS-2 is not relevant because the article is a literature review, not a primary study evaluating a diagnostic test. The review summarizes observational data on physical activity and mental health, not diagnostic accuracy. Bias in the context of a literature review (e.g., selection of studies, data synthesis) would be better assessed using tools like AMSTAR-2 or ROBIS, which are designed for systematic reviews or literature reviews. | the risk of bias assessment for the "Index Test" domain is also not applicable. QUADAS-2 is designed for diagnostic accuracy studies, and this literature review does not fit that format for this particular domain. | |
| | | | | |
| **Reference standard** | | | | |
| **Signaling questions:**<br><br>Is the reference standard likely to correctly classify the target condition?<br>Were the reference standard results interpreted without knowledge of the results of the index test?<br>Risk of bias assessment | **Answers (QUADAS-2: Domain 3 – Reference Standard):**<br>**Is the reference standard likely to correctly classify the target condition? → Not applicable**<br>**Were the reference standard results interpreted without knowledge of the results of the index test? → Not applicable**<br>**Risk of bias: → Not applicable**<br>**Rationale:**<br>As a **narrative review**, the article **does not employ or evaluate a reference standard** for diagnosing any specific target condition.<br>No diagnostic comparisons or reference classifications are made; instead, the paper discusses associations | *Signaling Questions and Answers*<br><br>**Is the reference standard likely to correctly classify the target condition?**<br>**Answer**: Not applicable.<br>**Were the reference standard results interpreted without knowledge of the results of the index test?**<br>**Answer**: Not applicable.<br><br>*Risk of Bias for Reference Standard*<br><br>**Risk of Bias**: Not applicable.<br><br>*Applicability Concerns for Reference Standard*<br><br>**Applicability Concerns**: Not applicable.<br><br>Rationale<br><br>The article by Muntean et al. (2025), titled "Impact of COVID-19 pandemic on physical activity levels and mental health in university students," is a literature review | Okay, here's the assessment of the "Reference Standard" domain:<br>Is the reference standard likely to correctly classify the target condition? **Unclear**<br>Were the reference standard results interpreted without knowledge of the results of the index test? **Not applicable**<br>Risk of bias assessment:<br>**Unclear**<br>**Rationale:**<br>**Is the reference standard likely to correctly classify the target condition? Unclear.** In this context, the "reference standard" refers to the methods used by the primary studies included in the review to measure physical activity and mental health. The review mentions that online surveys were the primary method of data collection. However, it doesn't provide a detailed evaluation of the validity and | **Signaling Question 1:** Is the reference standard likely to correctly classify the target condition?<br>**Answer:** Not applicable<br>**Signaling Question 2:** Were the reference standard results interpreted without knowledge of the index test?<br>**Answer:** Not applicable<br>**Risk of Bias:** Unclear<br>**Rationale:**<br>The concept of a "reference standard" does not apply to literature reviews assessing physical activity and mental health impacts. |

| | | | | |
|---|---|---|---|---|
| | between physical activity and mental health based on existing literature. Therefore, this domain is **not applicable** to the study design. | synthesizing findings from 47 studies. The QUADAS-2 tool, specifically the Reference Standard domain, is designed to assess bias in primary diagnostic accuracy studies where a reference standard (the gold standard test) is used to confirm the presence or absence of a target condition. This domain is not applicable to a literature review for the following reasons:<br><br>**Is the reference standard likely to correctly classify the target condition?** This question evaluates whether the reference standard in a diagnostic study accurately identifies the condition being tested (e.g., disease presence). The article does not involve diagnostic testing or a target condition requiring classification. Instead, it reviews studies assessing physical activity levels (e.g., via surveys or fitness trackers) and mental health outcomes (e.g., anxiety, depression) using tools like questionnaires or self-reports. These measures are not reference standards for diagnosing a condition but rather observational data collection methods. Thus, this question is irrelevant.<br>**Were the reference standard results interpreted without knowledge of the results of the index test?** This question assesses whether the reference standard's interpretation was blinded to the index test results to prevent bias. Since the reviewed studies do not use an index test or reference standard for diagnostic purposes, this question does not apply. The studies primarily involve cross-sectional surveys or self-reported outcomes, with no comparison between a diagnostic test and a gold standard.<br><br>**Risk of Bias and Applicability**: The Reference Standard domain of QUADAS-2 is not applicable because the article is a | reliability of these surveys or other methods used in the primary studies. The validity and reliability of the reference standards (e.g., surveys, fitness trackers, clinical assessments) in the primary studies determine how accurately physical activity and mental health were classified. Since this information is not thoroughly reported in the review, it's unclear whether the reference standards used in the primary studies are adequate.<br>**Were the reference standard results interpreted without knowledge of the results of the index test? Not applicable.** This question is not directly applicable to a literature review. The review authors are summarizing and analyzing already existing data; they are not involved in the original interpretation of reference standard results in individual participants.<br>**Risk of bias assessment: Unclear.** Because the review does not provide sufficient information to assess the validity and reliability of the reference standards used in the primary studies, the risk of bias for the "Reference Standard" domain is rated as unclear. | |

| | | | | |
|---|---|---|---|---|
| | | literature review, not a primary diagnostic accuracy study. The review synthesizes data on physical activity and mental health trends, not diagnostic test performance. Bias related to the review's methodology (e.g., study selection, data extraction) would be better evaluated using tools like AMSTAR-2 or ROBIS, which are suited for assessing literature reviews.<br>If you have further signaling questions for the remaining QUADAS-2 domain (Flow and Timing) or prefer an alternative quality assessment for the literature review, please let me know! | | |
| | | | | |
| Flow and timing | | | | |
| **Signaling questions:**<br><br>Was there an appropriate interval between index test(s) and reference standard?<br>Did all patients receive a reference standard?<br>Did patients receive the same reference standard?<br>Were all patients included in the analysis?<br>Risk of bias assessment | **Answers (QUADAS-2: Domain 4 – Flow and Timing):**<br>**Was there an appropriate interval between index test(s) and reference standard? → Not applicable**<br>**Did all patients receive a reference standard? → Not applicable**<br>**Did patients receive the same reference standard? → Not applicable**<br>**Were all patients included in the analysis? → Not applicable**<br>**Risk of bias: → Not applicable**<br>**Rationale:**<br>The study is a **narrative literature review** and does **not involve patient flow or application of diagnostic tests** over time.<br>There are **no individual participants**, no index or reference tests, and thus no | **Signaling Questions and Answers**<br>**Was there an appropriate interval between index test(s) and reference standard?**<br>**Answer**: Not applicable.<br>**Did all patients receive a reference standard?**<br>**Answer**: Not applicable.<br>**Did patients receive the same reference standard?**<br>**Answer**: Not applicable.<br>**Were all patients included in the analysis?**<br>**Answer**: Not applicable.<br>**Risk of Bias for Flow and Timing**<br>**Risk of Bias**: Not applicable.<br>**Applicability Concerns for Flow and Timing**<br>**Applicability Concerns**: Not applicable.<br>**Rationale**<br>The article by Muntean et al. (2025), titled "Impact of COVID-19 pandemic on physical activity levels and mental health in university students," is a literature review synthesizing findings from 47 studies. The QUADAS-2 tool, specifically the Flow and Timing domain, is designed to assess bias in primary diagnostic accuracy studies, focusing on the timing and consistency of index test and reference standard | Okay, here's the assessment of the "Flow and Timing" domain:<br>Was there an appropriate interval between index test(s) and reference standard? **Not applicable**<br>Did all patients receive a reference standard? **Not applicable**<br>Did patients receive the same reference standard? **Not applicable**<br>Were all patients included in the analysis? **Unclear**<br>Risk of bias assessment: **Unclear**<br>**Rationale:**<br>**Was there an appropriate interval between index test(s) and reference standard? Not applicable.** This question is relevant for primary diagnostic accuracy studies where there's a time interval between the index test and the reference standard. In a literature review, the authors are summarizing findings from various studies, | **Signaling Question 1:** Was there an appropriate interval between index test and reference standard?<br>**Answer:** Not applicable<br>**Signaling Question 2:** Did all patients receive the same reference standard?<br>**Answer:** Not applicable<br>**Signaling Question 3:** Were all patients included in the analysis?<br>**Answer:** Unclear<br>**Risk of Bias:** Unclear<br>**Rationale:**<br>The study synthesized 47 articles, but it is unclear if all eligible studies were included or if attrition bias exists in the original studies (Unclear).<br><br>**Note:** QUADAS-2 is tailored for diagnostic accuracy studies, so its application to this literature review is limited. A more appropriate tool (e.g., AMSTAR-2 for systematic reviews) might yield more relevant insights. |

| | | | | |
|---|---|---|---|---|
| | timing or inclusion concerns as relevant to a diagnostic accuracy study.<br>Consequently, this domain is **not applicable** to the study design. | administration, as well as patient inclusion in the analysis. This domain is not applicable to a literature review for the following reasons:<br>**Was there an appropriate interval between index test(s) and reference standard?**<br>This question evaluates whether the time gap between the index test and reference standard in a diagnostic study could affect results (e.g., due to disease progression). The article does not involve diagnostic tests or reference standards. The reviewed studies primarily used surveys, wearable fitness trackers, or self-reports to measure physical activity and mental health outcomes, with no temporal comparison between tests. Thus, this question is irrelevant.<br>**Did all patients receive a reference standard?**<br>This question assesses whether all participants in a diagnostic study underwent the reference standard to confirm the condition. The article reviews observational studies, not diagnostic ones, and there is no reference standard or patient cohort receiving diagnostic testing. The focus is on population-level trends, making this question inapplicable.<br>**Did patients receive the same reference standard?**<br>This question checks for consistency in the reference standard applied across patients in a diagnostic study. Since the reviewed studies do not involve diagnostic testing or a reference standard, and instead use varied methods (e.g., different surveys or fitness trackers), this question does not apply.<br>**Were all patients included in the analysis?**<br>This question evaluates whether all enrolled patients in a diagnostic study were included in the final analysis to avoid selection bias. The article is a literature review, not a primary study with patient enrollment. It synthesizes data from 47 studies, with study selection based on inclusion criteria (e.g., | and the concept of a specific interval between "tests" doesn't apply in the same way.<br>**Did all patients receive a reference standard? Not applicable.** This is also not applicable to a literature review. The review is analyzing already completed studies, not individual patients.<br>**Did patients receive the same reference standard? Not applicable.** Similar to the above, this question doesn't apply to a literature review. The review synthesizes findings from different studies that may have used various reference standards.<br>**Were all patients included in the analysis? Unclear.** In the context of a review, this refers to whether all studies identified by the authors were included in the final analysis. The authors initially identified 97 articles, screened them, removed duplicates, and assessed 75 for eligibility, finally including 47 studies in their analysis. While they explain their selection process, it's unclear if there was a risk of bias in excluding the other studies. The rationale for excluding 28 studies is not detailed enough to determine if it was appropriate and free from bias.<br>**Risk of bias assessment: Unclear.** The risk of bias for this domain is rated as unclear because, while the study provides a flow of included studies, the potential for bias | |

| | | | | |
|---|---|---|---|---|
| | | publication date, language), not patient-level analysis. Thus, this question is irrelevant.<br>**Risk of Bias and Applicability**: The Flow and Timing domain of QUADAS-2 is not applicable because the article is a literature review, not a primary diagnostic accuracy study. The review summarizes observational data on physical activity and mental health, with no patient-level diagnostic testing or follow-up. Bias related to the review's methodology (e.g., study selection, data synthesis) would be better assessed using tools like AMSTAR-2 or ROBIS, designed for literature reviews.<br>If you have additional questions or would like an alternative quality assessment (e.g., AMSTAR-2) for this literature review, please let me know! | in the study selection process is not fully clear. | |
| | | | | |

| **Popovici, 2025** | | | | |
|---|---|---|---|---|
| **Human** | **ChatGPT 4o** | **x. AI Grok 3** | **Gemini 2.0 flash** | **DeepSeek V3** |
| **Patient selection** | | | | |
| **Signaling questions:**<br>Was a consecutive or random sample of patients enrolled?<br>Was a case-control design avoided?<br>Did the study avoid inappropriate exclusions?<br>**Risk of bias:** | **Answers to Signaling Questions for the Patient Selection Domain:**<br>**Was a consecutive or random sample of patients enrolled?**<br>**Answer:** No<br>**Was a case-control design avoided?**<br>**Answer:** No<br>**Did the study avoid inappropriate exclusions?**<br>**Answer:** Unclear<br>**Risk of bias assessment:**<br>**Answer:** High<br>**Rationale:**<br>The study is a **narrative review**, not a primary diagnostic accuracy study. As such, it did not involve | The QUADAS-2 (Quality Assessment of Diagnostic Accuracy Studies) tool is designed to assess the risk of bias and applicability concerns in studies evaluating diagnostic accuracy. However, the provided article, "Dental Splints and Sport Performance: A Review of the Current Literature" by Popovici et al. (2025), is a systematic review, not a diagnostic accuracy study. QUADAS-2 is not applicable for assessing systematic reviews, as it is tailored for primary studies evaluating diagnostic tests against a reference standard.<br>Instead, systematic reviews are typically assessed using tools like AMSTAR-2 (A Measurement Tool to Assess Systematic Reviews) or ROBIS (Risk of Bias in Systematic Reviews). Since the user specifically requested the QUADAS-2 tool, I will clarify why it cannot be applied and provide a brief rationale. If the user prefers, I | Interogare reușită<br><br>Here are the answers to your signaling questions for the "Patient Selection" domain based on the provided article:<br>Was a consecutive or random sample of patients enrolled? **No**<br>Was a case-control design avoided? **Yes**<br>Did the study avoid inappropriate exclusions? **Unclear**<br>Risk of bias assessment: **Unclear**<br>**Rationale:**<br>**Was a consecutive or random sample of patients enrolled?** The study used a systematic review design, not | **Signaling Question 1:** Was a consecutive or random sample of patients enrolled?<br>**Answer:** Unclear<br>**Rationale:** The article is a review of existing literature, not a primary study with patient enrollment, so this question is not directly applicable.<br>**Signaling Question 2:** Was a case-control design avoided?<br>**Answer:** Yes<br>**Rationale:** The study is a systematic review, not a case-control study.<br>**Signaling Question 3:** Did the study avoid inappropriate exclusions?<br>**Answer:** Yes<br>**Rationale:** The review clearly outlines inclusion and exclusion criteria, and the selection process is described in detail.<br>**Risk of Bias:** Low |

| | | | | |
|---|---|---|---|---|
| | enrollment of patients, let alone a **consecutive or random sample**, and **used studies selected based on inclusion/exclusion criteria** for literature, not clinical enrollment.<br>A **case-control design was not applicable** in the traditional diagnostic test sense, but many included studies in the review compared athletes with and without splints (or interventions), **analogous to a case-control structure**, which is generally discouraged in diagnostic test accuracy studies due to high risk of bias.<br>The review **excluded non-English and non-human studies**, but it's unclear whether other relevant studies might have been inappropriately excluded without justification.<br>Given that this is not a primary diagnostic study and does not meet the structural expectations for a QUADAS-2 diagnostic review, **the risk of bias for the patient selection domain is high**. | can assess the article using an appropriate tool for systematic reviews or proceed with a modified QUADAS-2 application for illustrative purposes, but this would not be standard practice.<br><br>QUADAS-2 Assessment Attempt<br><br>**Domain 1: Patient Selection**<br><br>Signaling Questions: Not applicable<br>Risk of Bias: Not applicable<br><br>**Domain 2: Index Test**<br><br>Signaling Questions: Not applicable<br>Risk of Bias: Not applicable<br><br>**Domain 3: Reference Standard**<br><br>Signaling Questions: Not applicable<br>Risk of Bias: Not applicable<br><br>**Domain 4: Flow and Timing**<br><br>Signaling Questions: Not applicable<br>Risk of Bias: Not applicable<br><br>**Applicability Concerns**<br><br>Patient Selection: Not applicable<br>Index Test: Not applicable<br>Reference Standard: Not applicable<br><br>Rationale<br><br>The QUADAS-2 tool evaluates four domains (patient selection, index test, reference standard, and flow and timing) to assess bias and applicability in diagnostic accuracy studies. These studies typically | enrolling patients directly. It searched for articles in databases, so there was no patient enrollment.<br>**Was a case-control design avoided?** The study is a systematic review of literature, not a primary study design that would use a case-control.<br>**Did the study avoid inappropriate exclusions?** The exclusion criteria are listed, but it's unclear if they were appropriate or if there were other exclusions that should have been avoided.<br>**Risk of bias assessment:** Due to the unclear risk of inappropriate exclusions, the overall risk of bias for patient selection is unclear. | |

| | | | | |
|---|---|---|---|---|
| | | compare a diagnostic test (index test) against a gold standard (reference standard) in a defined population. The article by Popovici et al. is a systematic review synthesizing evidence on the effects of occlusal splints on sports performance, not a primary study assessing diagnostic accuracy. It does not involve a diagnostic test, reference standard, or patient selection for diagnostic purposes, rendering QUADAS-2 inapplicable.<br>The review focuses on outcomes like athletic performance (e.g., strength, balance, jump height) rather than diagnostic metrics (e.g., sensitivity, specificity). The methodology involves a literature search, study selection, and synthesis of results, which aligns with systematic review processes, not diagnostic study designs. Applying QUADAS-2 would be inappropriate, as its signaling questions (e.g., "Was a consecutive or random sample of patients enrolled?" or "Was the reference standard likely to correctly classify the target condition?") do not correspond to the review's structure or objectives.<br><br>Recommendation<br><br>If the user wishes to assess the quality of this systematic review, I recommend using AMSTAR-2 or ROBIS, which are designed for systematic reviews. I can perform such an assessment if requested. Alternatively, if the user insists on QUADAS-2, I can attempt a non-standard application by analogy, but this would require reinterpreting the domains in a way that deviates from QUADAS-2's intended use. Please clarify how to proceed.<br><br>Since the provided article, "Dental Splints and Sport Performance: A Review of the Current Literature" by Popovici et al. (2025), is a systematic review and not a primary diagnostic accuracy study, the QUADAS-2 tool is not directly applicable, as noted | | |

| | | | | |
|---|---|---|---|---|
| | | previously. The signaling questions for Domain 1 (Patient Selection) are designed for primary studies with patient cohorts, not for systematic reviews that synthesize existing studies. However, per your request to answer the specific signaling questions for Domain 1 and provide a risk of bias assessment, I will address each question as "not applicable" due to the study design and provide a rationale for why these questions do not fit. The risk of bias will also be marked as "not applicable" to reflect the mismatch between QUADAS-2 and the article's methodology.<br>**QUADAS-2 Domain 1: Patient Selection**<br>**Was a consecutive or random sample of patients enrolled?**<br>Answer: Not applicable<br>**Was a case-control design avoided?**<br>Answer: Not applicable<br>**Did the study avoid inappropriate exclusions?**<br>Answer: Not applicable<br>**Risk of Bias Assessment for Patient Selection**<br>Answer: Not applicable<br>**Rationale**<br>**Was a consecutive or random sample of patients enrolled?** This question is irrelevant because the article is a systematic review, not a primary study enrolling patients. Instead, it involves selecting studies from databases (PubMed, Scopus, Web of Science) based on inclusion criteria (e.g., open-access studies on dental occlusion and sports performance, published 2014–2024). The concept of patient sampling does not apply; the review focuses on study selection, not patient recruitment. The PRISMA flow diagram (Figure 1) details the study selection process, starting with 128 records and ending with 24 included studies, but this is not equivalent to patient enrollment.<br>**Was a case-control design avoided?** This question is also not applicable. Systematic | | |

| | | | | |
|---|---|---|---|---|
| | | reviews do not employ study designs like case-control; they aggregate data from primary studies, which may have various designs (e.g., randomized trials, observational studies). The review does not report conducting a primary case-control study. The included studies (listed in Table 2) involve interventions like occlusal splints and outcomes like jump performance or strength, but the review itself does not adopt a case-control framework. The PICO question (patients: athletes; intervention: dental occlusion; comparison: normal occlusion vs. anomalies; outcome: performance) guides study inclusion, not a case-control design.<br>**Did the study avoid inappropriate exclusions?** This question is inapplicable because the review does not exclude patients but rather studies based on predefined criteria. The inclusion criteria (open-access, English-language, full-text studies on dental occlusion and sports performance) and exclusion criteria (e.g., conference presentations, in vitro/animal studies, meta-analyses) are clearly stated. While inappropriate study exclusions could introduce bias in a systematic review, this is assessed using tools like AMSTAR-2 or ROBIS, not QUADAS-2, which focuses on patient exclusions in primary diagnostic studies. The review's selection process (two reviewers, manual screening, PRISMA compliance) suggests a systematic approach, but QUADAS-2's patient-focused lens does not align.<br>**Risk of Bias Assessment for Patient Selection** The risk of bias cannot be assessed using QUADAS-2's patient selection domain because the article does not involve patient selection. Systematic reviews are at risk of bias due to factors like incomplete literature searches, biased study selection, or inadequate quality assessment of included studies, which are not covered by | | |

| | | | | |
|---|---|---|---|---|
| | | QUADAS-2. The review's quality would be better evaluated with a tool like AMSTAR-2, which examines protocol registration, search comprehensiveness, and study quality assessment. Marking the risk as "not applicable" reflects the tool's incompatibility with the article's design.<br>**Additional Notes**<br>The systematic review's methodology (PRISMA-compliant, clear inclusion/exclusion criteria, two reviewers for study selection) suggests efforts to minimize bias in study selection, but these aspects fall outside QUADAS-2's scope. If you wish to proceed with the remaining QUADAS-2 domains (Index Test, Reference Standard, Flow and Timing) or prefer an assessment using a systematic review-specific tool (e.g., AMSTAR-2 or ROBIS), please let me know. Alternatively, I can reinterpret QUADAS-2 domains analogously for the review (e.g., treating "patient selection" as "study selection"), but this would deviate from standard QUADAS-2 use. Please clarify your preference. | | |
| | | | | |
| **Index test** | | | | |
| **Signaling questions:**<br><br>Were the index test results interpreted without knowledge of the results of the reference standard?<br>If a threshold was used, was it pre-specified?<br>Risk of bias assessment | **Answers to Signaling Questions for the Index Test Domain:**<br>**Were the index test results interpreted without knowledge of the results of the reference standard?**<br>**Answer:** Unclear<br>**If a threshold was used, was it pre-specified?**<br>**Answer:** Unclear<br>**Risk of bias assessment:**<br>**Answer:** Unclear<br><br>**Rationale:**<br>The article is a **review**, not a primary diagnostic study, and thus **does not directly** | The article, "Dental Splints and Sport Performance: A Review of the Current Literature" by Popovici et al. (2025), is a systematic review, not a primary diagnostic accuracy study. As previously noted, the QUADAS-2 tool is designed for diagnostic accuracy studies and is not directly applicable to systematic reviews, which do not involve an index test or reference standard. The signaling questions for Domain 2 (Index Test) of QUADAS-2 are tailored to studies evaluating a diagnostic test's performance against a gold standard, which does not align with the review's methodology of synthesizing studies on occlusal splints and sports performance. Per your request to answer the specific signaling questions for Domain 2 and provide a risk of | Here are the answers to your signaling questions for the "Index Test" domain:<br>Were the index test results interpreted without knowledge of the results of the reference standard? **Not applicable**<br>If a threshold was used, was it pre-specified? **Not applicable**<br>Risk of bias assessment: **Low**<br>**Rationale:**<br>Were the index test results interpreted without knowledge of the results of the reference standard? This question is not applicable because the study is a systematic review. It does not involve a specific index | **Signaling Question 1:** Were the index test results interpreted without knowledge of the reference standard?<br>**Answer:** Unclear<br>**Rationale:** The review synthesizes findings from multiple studies, and it is unclear whether the authors were blinded to the outcomes of the included studies during analysis.<br>**Signaling Question 2:** If a threshold was used, was it pre-specified?<br>**Answer:** Not applicable<br>**Rationale:** The review does not involve a diagnostic threshold.<br>**Risk of Bias:** Unclear |

| | | | | |
|---|---|---|---|---|
| | **report or interpret any index test results** itself. It synthesizes findings from various included studies evaluating the effects of occlusal splints, which are not used as diagnostic tools but rather as **interventions** or **performance modifiers**. Since **no formal diagnostic index test is evaluated**, there is **no direct reference standard comparison**, making it impossible to judge whether interpretations were blinded or thresholds were pre-specified across all included studies.<br>As such, due to the **lack of a clearly defined index test or diagnostic threshold**, both signaling questions are marked as **unclear**, and the **risk of bias for this domain is rated as unclear**. | bias assessment, I will mark each question as "not applicable" due to the study design and explain why these questions do not fit. The risk of bias will also be marked as "not applicable" to reflect the mismatch between QUADAS-2 and the article's structure.<br><br>QUADAS-2 Domain 2: Index Test<br><br>**Were the index test results interpreted without knowledge of the results of the reference standard?**<br>Answer: Not applicable<br>**If a threshold was used, was it pre-specified?**<br>Answer: Not applicable<br>**Risk of Bias Assessment for Index Test**<br>Answer: Not applicable<br><br>Rationale<br><br>**Were the index test results interpreted without knowledge of the results of the reference standard?** This question is not applicable because the article is a systematic review that does not involve an index test or reference standard. In QUADAS-2, the index test refers to the diagnostic test being evaluated (e.g., a biomarker or imaging technique), and the reference standard is the gold standard used to confirm the diagnosis. The review synthesizes evidence from studies examining the effects of occlusal splints on sports performance outcomes (e.g., strength, balance, jump height), not diagnostic test results. The included studies (listed in Table 2) report interventions like mouthguards or splints and outcomes like countermovement jump or electromyography data, none of which are diagnostic tests. The review's methodology focuses on study selection and data synthesis, not interpreting test results, rendering this question irrelevant. | test or reference standard in the way a primary diagnostic accuracy study would. The review analyzes previously published studies.<br>If a threshold was used, was it pre-specified? Not applicable for the same reason as above; a systematic review does not establish or use thresholds in the same way a primary study does.<br>Risk of bias assessment: Low. In a systematic review, the concept of "blinding" to a reference standard doesn't apply. The risk of bias related to the index test in a review is more about how the review authors selected and interpreted the included studies, which will be assessed in other domains. For this specific domain, the risk of bias is low because the questions are not applicable. | |

| | | | | |
|---|---|---|---|---|
| | | **If a threshold was used, was it pre-specified?** This question is also not applicable. In diagnostic accuracy studies, thresholds refer to cut-off values used to classify test results (e.g., positive or negative for a condition). The systematic review does not involve diagnostic thresholds, as it evaluates the impact of occlusal splints on performance metrics, not diagnostic classifications. The included studies report continuous outcomes (e.g., jump height, force production) or statistical significance (e.g., p-values), not binary diagnostic thresholds. The review's search and inclusion criteria (e.g., studies from 2014–2024, English-language, open-access) are pre-specified, but these are methodological criteria, not diagnostic thresholds, and are outside the scope of QUADAS-2's index test domain.<br>**Risk of Bias Assessment for Index Test**<br>The risk of bias cannot be assessed using QUADAS-2's index test domain because the article does not involve an index test. Systematic reviews are subject to biases related to literature search comprehensiveness, study selection, or data synthesis, which are not addressed by QUADAS-2. For example, the review's use of PRISMA guidelines, two reviewers for study screening, and clear inclusion/exclusion criteria suggests efforts to reduce bias, but these are evaluated using tools like AMSTAR-2 or ROBIS, not QUADAS-2. Marking the risk as "not applicable" reflects the tool's incompatibility with the review's design.<br><br>Additional Notes<br><br>The systematic review's methodology (e.g., PRISMA compliance, Boolean search strategy, two-reviewer screening) indicates a structured approach to minimize bias in | | |

| | | | | |
|---|---|---|---|---|
| | | study selection and data synthesis, but these aspects are not captured by QUADAS-2's index test domain. If you wish to proceed with the remaining QUADAS-2 domains (Reference Standard, Flow and Timing) or prefer an assessment using a tool designed for systematic reviews (e.g., AMSTAR-2 or ROBIS), please let me know. Alternatively, I can attempt a non-standard application of QUADAS-2 by analogizing "index test" to the review's intervention (occlusal splints), but this would deviate from the tool's intended use. Please clarify your preference. | | |
| | | | | |
| **Reference standard** | | | | |
| **Signaling questions:**<br><br>Is the reference standard likely to correctly classify the target condition?<br>Were the reference standard results interpreted without knowledge of the results of the index test?<br>Risk of bias assessment | **Answers to Signaling Questions for the Reference Standard Domain:**<br>**Is the reference standard likely to correctly classify the target condition?**<br>**Answer:** Not applicable<br>**Were the reference standard results interpreted without knowledge of the results of the index test?**<br>**Answer:** Not applicable<br>**Risk of bias assessment:**<br>**Answer:** Not applicable<br><br>**Rationale:**<br>The study **does not involve a diagnostic accuracy assessment**, and thus **no reference standard is defined or used** to classify a target condition.<br>It is a **narrative/systematic review of intervention effects** (occlusal splints and sports performance), rather than a study comparing an | The article, "Dental Splints and Sport Performance: A Review of the Current Literature" by Popovici et al. (2025), is a systematic review, not a primary diagnostic accuracy study. As previously stated, the QUADAS-2 tool is designed for diagnostic accuracy studies and is not directly applicable to systematic reviews, which do not involve an index test or reference standard. The signaling questions for Domain 3 (Reference Standard) of QUADAS-2 are intended for studies evaluating a diagnostic test against a gold standard, which does not align with the review's methodology of synthesizing evidence on the effects of occlusal splints on sports performance. Per your request to answer the specific signaling questions for Domain 3 and provide a risk of bias assessment, I will mark each question as "not applicable" due to the study design and explain why these questions are inappropriate. The risk of bias will also be marked as "not applicable" to reflect the incompatibility between QUADAS-2 and the article's structure. | Here are the answers to your signaling questions for the "Reference Standard" domain:<br>Is the reference standard likely to correctly classify the target condition? **Not applicable**<br>Were the reference standard results interpreted without knowledge of the results of the index test? **Not applicable**<br>Risk of bias assessment: **Not applicable**<br>**Rationale:**<br>Is the reference standard likely to correctly classify the target condition? This question is not applicable because the study is a systematic review. There isn't a single reference standard as in a primary diagnostic study. The "target condition" is the relationship between dental occlusion and sports performance, and the review synthesizes evidence from various studies.<br>Were the reference standard results interpreted without knowledge of the results of the | **Signaling Question 1:** Is the reference standard likely to correctly classify the target condition?<br>**Answer:** Yes<br>**Rationale:** The reference standard in the included studies (e.g., EMG, balance tests, strength measurements) are appropriate for assessing the effects of occlusal splints on performance.<br>**Signaling Question 2:** Were the reference standard results interpreted without knowledge of the index test?<br>**Answer:** Unclear<br>**Rationale:** The review does not provide details on whether the reference standard assessments in the included studies were blinded.<br>**Risk of Bias:** Unclear |

| | | | | |
|---|---|---|---|---|
| | index test against a gold standard diagnosis. Consequently, both signaling questions and the risk of bias domain are **not applicable** in this context. | QUADAS-2 Domain 3: Reference Standard<br><br>**Is the reference standard likely to correctly classify the target condition?**<br>Answer: Not applicable<br>**Were the reference standard results interpreted without knowledge of the results of the index test?**<br>Answer: Not applicable<br>**Risk of Bias Assessment for Reference Standard**<br>Answer: Not applicable<br><br>Rationale<br><br>**Is the reference standard likely to correctly classify the target condition?** This question is not applicable because the systematic review does not involve a reference standard or a target condition in the diagnostic sense. In QUADAS-2, the reference standard is the gold standard test used to confirm the presence or absence of a specific condition (e.g., a biopsy for cancer diagnosis). The review synthesizes studies examining the impact of occlusal splints on sports performance outcomes (e.g., strength, balance, jump height), not diagnostic classifications. The included studies (Table 2) report interventions like occlusal splints or mouthguards and measure performance metrics (e.g., countermovement jump, electromyography), none of which involve a diagnostic "target condition" or a reference standard to classify it. The review's focus is on aggregating evidence, not evaluating diagnostic accuracy, making this question irrelevant.<br>**Were the reference standard results interpreted without knowledge of the results of the index test?** This question is also not applicable. In diagnostic accuracy studies, this question assesses whether the reference standard's results are interpreted | index test? Not applicable, for the same reason. This review is analyzing already completed studies, not conducting new index tests or applying reference standards.<br>Risk of bias assessment: Not applicable. Since the questions themselves are not applicable, there is no risk of bias to assess in the context of a reference standard. | |

| | | | | |
|---|---|---|---|---|
| | | independently to avoid bias from knowledge of the index test (the test under evaluation). Since the review does not involve an index test or reference standard, there are no diagnostic test results to interpret. The review's methodology involves screening and synthesizing studies based on inclusion criteria (e.g., open-access studies from 2014–2024 on dental occlusion and sports performance), with two reviewers assessing study eligibility. This process does not involve blinding in the context of diagnostic test interpretation, as it is not a diagnostic study. The question does not apply to the review's design.<br>**Risk of Bias Assessment for Reference Standard** The risk of bias cannot be assessed using QUADAS-2's reference standard domain because the article does not involve a reference standard. Systematic reviews face biases related to study selection, data extraction, or quality assessment of included studies, which are not addressed by QUADAS-2. The review's adherence to PRISMA guidelines, use of two reviewers for study selection, and clear reporting of inclusion/exclusion criteria suggest efforts to minimize bias, but these are evaluated using tools like AMSTAR-2 or ROBIS, not QUADAS-2. Marking the risk as "not applicable" reflects the tool's mismatch with the review's methodology.<br><br>Additional Notes<br><br>The systematic review's structured approach (e.g., PRISMA compliance, clear search strategy, two-reviewer screening) indicates efforts to reduce bias in evidence synthesis, but these aspects fall outside QUADAS-2's reference standard domain. If you wish to proceed with the final QUADAS-2 domain (Flow and Timing) or prefer an assessment using a tool designed for systematic reviews | | |

| | | | | |
|---|---|---|---|---|
| | | (e.g., AMSTAR-2 or ROBIS), please let me know. Alternatively, I can attempt a non-standard application of QUADAS-2 by analogizing "reference standard" to a component of the review (e.g., outcome measures in included studies), but this would deviate from the tool's intended use. Please clarify your preference. | | |
| | | | | |
| Flow and timing | | | | |
| **Signaling questions:**<br><br>Was there an appropriate interval between index test(s) and reference standard?<br>Did all patients receive a reference standard?<br>Did patients receive the same reference standard?<br>Were all patients included in the analysis?<br>Risk of bias assessment | **Answers to Signaling Questions for the Flow and Timing Domain:**<br>**Was there an appropriate interval between index test(s) and reference standard?**<br>**Answer:** Not applicable<br>**Did all patients receive a reference standard?**<br>**Answer:** Not applicable<br>**Did patients receive the same reference standard?**<br>**Answer:** Not applicable<br>**Were all patients included in the analysis?**<br>**Answer:** Not applicable<br>**Risk of bias assessment:**<br>**Answer:** Not applicable<br><br>**Rationale:**<br>The article is a **review** and does **not involve any direct testing or patient flow**, nor does it evaluate the diagnostic accuracy of any test. Therefore, **no index test or reference standard is administered**, and **no patient inclusion or exclusion in a diagnostic process is applicable**.<br>All aspects of the Flow and Timing domain are **not applicable** because the | The article, "Dental Splints and Sport Performance: A Review of the Current Literature" by Popovici et al. (2025), is a systematic review, not a primary diagnostic accuracy study. As previously noted, the QUADAS-2 tool is designed for diagnostic accuracy studies and is not directly applicable to systematic reviews, which do not involve an index test, reference standard, or patient-level diagnostic assessments. The signaling questions for Domain 4 (Flow and Timing) of QUADAS-2 are intended for studies evaluating the timing and application of diagnostic tests in a patient cohort, which does not align with the review's methodology of synthesizing evidence on occlusal splints and sports performance. Per your request to answer the specific signaling questions for Domain 4 and provide a risk of bias assessment, I will mark each question as "not applicable" due to the study design and explain why these questions are inappropriate. The risk of bias will also be marked as "not applicable" to reflect the incompatibility between QUADAS-2 and the article's structure.<br><br>QUADAS-2 Domain 4: Flow and Timing<br><br>**Was there an appropriate interval between index test(s) and reference standard?**<br>Answer: Not applicable | Here are the answers to your signaling questions for the "Flow and Timing" domain:<br>Was there an appropriate interval between index test(s) and reference standard? **Not applicable**<br>Did all patients receive a reference standard? **Not applicable**<br>Did patients receive the same reference standard? **Not applicable**<br>Were all patients included in the analysis? **Unclear**<br>Risk of bias assessment: **Unclear**<br>**Rationale:**<br>Was there an appropriate interval between index test(s) and reference standard? This is not applicable because the study is a systematic review. The concepts of "index test" and "reference standard" and the interval between them do not apply in the same way as in a primary diagnostic accuracy study.<br>Did all patients receive a reference standard? Not applicable, for the same reason as above. | **Signaling Question 1:** Was there an appropriate interval between index test and reference standard?<br>**Answer:** Not applicable<br>**Rationale:** The review does not involve a specific interval between tests.<br>**Signaling Question 2:** Did all patients receive the same reference standard?<br>**Answer:** No<br>**Rationale:** The included studies used different reference standards (e.g., EMG, balance tests, strength measurements).<br>**Signaling Question 3:** Were all patients included in the analysis?<br>**Answer:** Yes<br>**Rationale:** The review appears to include all relevant studies meeting the inclusion criteria.<br>**Risk of Bias:** Low |

| | | | | |
|---|---|---|---|---|
| | study design **does not match the context for which the QUADAS-2 tool was developed** (i.e., diagnostic test accuracy studies with a reference standard). | **Did all patients receive a reference standard?**<br>Answer: Not applicable<br>**Did patients receive the same reference standard?**<br>Answer: Not applicable<br>**Were all patients included in the analysis?**<br>Answer: Not applicable<br>**Risk of Bias Assessment for Flow and Timing**<br>Answer: Not applicable<br><br>Rationale<br><br>**Was there an appropriate interval between index test(s) and reference standard?** This question is not applicable because the systematic review does not involve an index test or reference standard. In QUADAS-2, this question assesses whether the timing between the diagnostic test (index test) and the gold standard (reference standard) is appropriate to ensure accurate results (e.g., avoiding changes in disease status). The review synthesizes studies examining the effects of occlusal splints on sports performance outcomes (e.g., jump height, strength, balance), not diagnostic tests. The included studies (Table 2) report interventions like occlusal splints and performance metrics, with no diagnostic testing or timing intervals relevant to a reference standard. The review's methodology focuses on study selection and data synthesis, making this question irrelevant.<br>**Did all patients receive a reference standard?** This question is not applicable because the review does not involve patients receiving diagnostic tests or a reference standard. QUADAS-2 uses this question to check if all patients in a diagnostic study are verified with the gold standard to avoid bias from incomplete testing. The review | Did patients receive the same reference standard? Not applicable, for the same reason as above.<br>Were all patients included in the analysis? It is unclear whether all studies identified in the search were included in the final synthesis. The flow diagram shows 128 records identified, and after screening, 24 studies were included, but the reasons for excluding some studies are broad (e.g., "outside the subject"). It's unclear if this process introduced bias.<br>Risk of bias assessment: Unclear. The major concern is whether all relevant studies were included in the review. Since the exclusion process is not fully transparent, the risk of bias for flow and timing is unclear. | |

| | | | | |
|---|---|---|---|---|
| | | aggregates data from 24 studies (selected from 128 initial records) involving athletes and occlusal splints, not patient-level diagnostic assessments. The concept of patients receiving a reference standard does not apply to the review's design, which involves study-level inclusion based on criteria (e.g., open-access, English-language studies from 2014–2024).<br>**Did patients receive the same reference standard?** This question is also not applicable. In diagnostic accuracy studies, this question ensures consistency in the gold standard applied across patients to avoid bias from varying reference tests. Since the review does not involve a reference standard or patient-level diagnostics, this question is irrelevant. The included studies vary in design, interventions (e.g., custom splints, mouthguards), and outcomes (e.g., electromyography, jump performance), but this heterogeneity is a feature of the review's scope, not a diagnostic testing issue. The review's PRISMA flow diagram (Figure 1) details study selection, not patient testing.<br>**Were all patients included in the analysis?** This question is not applicable because the review does not involve a patient cohort or diagnostic analysis. QUADAS-2 uses this question to check if all enrolled patients are included in the final analysis to avoid bias from selective reporting. The review synthesizes data from 24 included studies, with exclusions based on predefined criteria (e.g., non-human studies, lack of original data). The concept of patient inclusion in analysis does not apply; instead, the review focuses on study inclusion, with two reviewers screening and selecting studies to minimize bias. This process is reported transparently but is outside QUADAS-2's patient-focused framework.<br>**Risk of Bias Assessment for Flow and Timing** The risk of bias cannot be assessed using QUADAS-2's flow and timing domain | | |

| | | | | |
|---|---|---|---|---|
| | | because the article does not involve diagnostic tests, reference standards, or patient-level assessments. Systematic reviews face biases related to study selection, data extraction, or incomplete reporting, which are not covered by QUADAS-2. The review's adherence to PRISMA guidelines, use of two reviewers, and clear reporting of the selection process (128 records to 24 studies) suggest efforts to reduce bias, but these are evaluated using tools like AMSTAR-2 or ROBIS, not QUADAS-2. Marking the risk as "not applicable" reflects the tool's incompatibility with the review's methodology.<br><br>Additional Notes<br><br>The systematic review's structured methodology (e.g., PRISMA compliance, Boolean search strategy, two-reviewer screening) indicates efforts to minimize bias in evidence synthesis, but these aspects are not captured by QUADAS-2's flow and timing domain. Since all QUADAS-2 domains (Patient Selection, Index Test, Reference Standard, Flow and Timing) have been addressed, the assessment confirms that QUADAS-2 is not suitable for this systematic review. If you wish to assess the review's quality using a tool designed for systematic reviews (e.g., AMSTAR-2 or ROBIS), I can perform such an analysis. Alternatively, I can summarize the QUADAS-2 findings or provide a non-standard interpretation of the domains tailored to the review, though this would deviate from the tool's intended use. Please clarify your preference for next steps. | | |
| | | | | |
